# CDK4-E2F3 signals enhance oxidative skeletal muscle fiber numbers and function to affect myogenesis and metabolism

**DOI:** 10.1172/JCI162479

**Published:** 2023-07-03

**Authors:** Young Jae Bahn, Hariom Yadav, Paolo Piaggi, Brent S. Abel, Oksana Gavrilova, Danielle A. Springer, Ioannis Papazoglou, Patricia M. Zerfas, Monica C. Skarulis, Alexandra C. McPherron, Sushil G. Rane

**Affiliations:** 1 Diabetes, Endocrinology and Obesity Branch, National Institute of Diabetes and Digestive and Kidney Diseases, NIH, Bethesda, Maryland, USA.; 2 Phoenix Epidemiology and Clinical Research Branch, National Institute of Diabetes and Digestive and Kidney Diseases, NIH, Phoenix, Arizona.; 3 Mouse Metabolism Core Facility, National Institute of Diabetes and Digestive and Kidney Diseases,; 4 Mouse Phenotyping Core, National Heart, Lung and Blood Institute,; 5 Office of Research Services, and; 6 Genetics of Development and Disease Branch, National Institute of Diabetes and Digestive and Kidney Diseases, NIH, Bethesda, Maryland, USA.

**Keywords:** Metabolism, Muscle Biology, Mitochondria, Skeletal muscle

## Abstract

Understanding how skeletal muscle fiber proportions are regulated is vital to understanding muscle function. Oxidative and glycolytic skeletal muscle fibers differ in their contractile ability, mitochondrial activity, and metabolic properties. Fiber-type proportions vary in normal physiology and disease states, although the underlying mechanisms are unclear. In human skeletal muscle, we observed that markers of oxidative fibers and mitochondria correlated positively with expression levels of *PPARGC1A* and *CDK4* and negatively with expression levels of *CDKN2A*, a locus significantly associated with type 2 diabetes. Mice expressing a constitutively active Cdk4 that cannot bind its inhibitor p16^INK4a^, a product of the *CDKN2A* locus, were protected from obesity and diabetes. Their muscles exhibited increased oxidative fibers, improved mitochondrial properties, and enhanced glucose uptake. In contrast, loss of Cdk4 or skeletal muscle–specific deletion of Cdk4’s target, E2F3, depleted oxidative myofibers, deteriorated mitochondrial function, and reduced exercise capacity, while increasing diabetes susceptibility. E2F3 activated the mitochondrial sensor PPARGC1A in a Cdk4-dependent manner. CDK4, E2F3, and PPARGC1A levels correlated positively with exercise and fitness and negatively with adiposity, insulin resistance, and lipid accumulation in human and rodent muscle. All together, these findings provide mechanistic insight into regulation of skeletal muscle fiber–specification that is of relevance to metabolic and muscular diseases.

## Graphical abstract

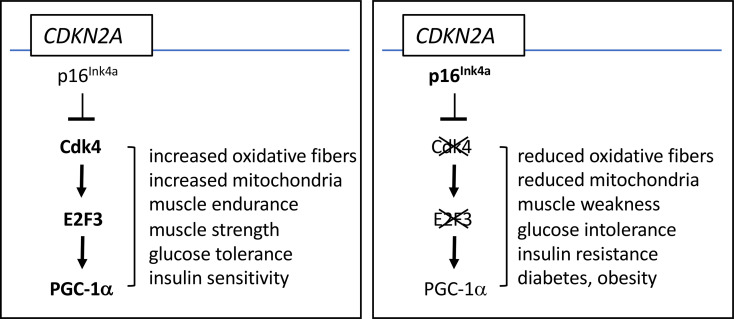

## Introduction

The variable temporal advancement of type 2 diabetes (T2D) characterized by progressive dysfunction in multiple targeted organs renders ineffective most therapeutic modalities (1–3). Inefficient glucose uptake by insulin-responsive tissues, chiefly skeletal muscle, is a hallmark of T2D pathogenesis (4, 5). This is hardly a surprise given that skeletal muscle accounts for approximately 40% of the body weight in nonobese individuals and about 20%–30% of total resting oxygen uptake (6, 7). Strikingly, 85% of the total amount of metabolized glucose is taken up by muscle, approximately 60% of which is stored as glycogen and approximately 20% of which is cleared via glucose oxidation (8, 9).

Skeletal muscle’s ability to perform diverse functions, including movement, thermogenesis, and metabolism, is largely attributed to the plasticity of muscle fibers that inherently differ in their contractile ability, mitochondrial content, and metabolic properties (10). Skeletal muscle fibers are broadly classified as slow-twitch oxidative type I, fast-twitch oxidative type IIA, and fast-twitch glycolytic type IIX and IIB fibers (11, 12). Type I and IIA fibers contain abundant mitochondria and primarily use oxidative metabolism, while type IIX and IIB fibers harbor limited mitochondria and rely on glycolytic metabolism (13, 14). Type I fibers are also more insulin sensitive compared with type IIB fibers (15–17). Individuals with T2D and obesity exhibit progressive muscle insulin resistance that is accompanied by changes in fiber-type proportions, specifically, reduced oxidative type I fibers and increased glycolytic type II fibers (15, 18, 19). Exercise training also involves muscle fiber–type switching along with increased mitochondrial biogenesis within muscle progenitors and/or muscle fibers (20, 21). These physiological and pathological examples of muscle fiber remodeling support the notion that appropriate muscle fiber distribution is of importance to optimal skeletal muscle function. However, the molecular signals that regulate muscle fiber proportions are not fully understood (21).

Unbiased T2D GWAS have unraveled important associations, including the surprising enrichment of loci that represent cell cycle genes (22, 23). The cell cycle pathway integrates diverse inputs from extracellular signaling networks that affect cell growth, proliferation, differentiation, and apoptosis (24). The cyclin-dependent kinases (Cdks), when associated with their regulatory cyclin proteins, phosphorylate downstream substrates, such as the retinoblastoma (RB) family of proteins, on distinct serine/threonine residues (24). In their underphosphorylated active state, the RB proteins associate with and inhibit a group of E2F transcription factors (25). The E2Fs, in addition to regulating S phase progression through the cell cycle, also control cell differentiation and cell specification. Phosphorylation by Cdks inactivates RB proteins and releases the associated E2Fs that, in turn, either activate or repress downstream target genes (25). Cdk activity is negatively regulated by Cdk inhibitors (CKIs) such as p16^Ink4a^ (26). Identified as a Cdk4-specific inhibitor, p16^Ink4a^ promotes cellular senescence, cell growth arrest, and tumor suppression (27).

Interestingly, the *CDKN2A* gene locus, which codes for p16^Ink4a^, is prominently associated with T2D in GWAS (22, 23, 28). Prior to these T2D GWAS linkages, our group demonstrated that Cdk4 deficiency led to hypoplasia of insulin-producing pancreatic islet β cells (β cells), whereas expression of constitutively active Cdk4^R24C^ kinase led to early commitment to the β cell lineage (29–31), β cell hyperplasia, and enhanced β cell regeneration (32). Studies have expanded to characterize the role of cell cycle molecules in other cells involved in glucose homeostasis (33). However, the larger relevance of the association of cell cycle loci, specifically the *CDKN2A* locus, in T2D GWAS has remained obscure (34).

Here, we report that p16^Ink4a^-Cdk4-E2F3 signals regulate muscle metabolic function via targeting the mitochondrial biosensor PPARγ coactivator 1-α (PPARGC1A, which encodes to PGC-1α) that is also linked to muscle fiber–type specification and metabolic disease (35–38). Considering the larger physiological role of skeletal muscle in glucose disposal, this study underscores the relevance of p16^Ink4a^-Cdk4 signals in T2D evolution and may help better understanding of the functional significance of GWAS findings associating the *CDKN2A* locus with T2D.

## Results

### Enhanced muscle metabolic function in Cdk4^R/R^ mice.

**Figure 1:**
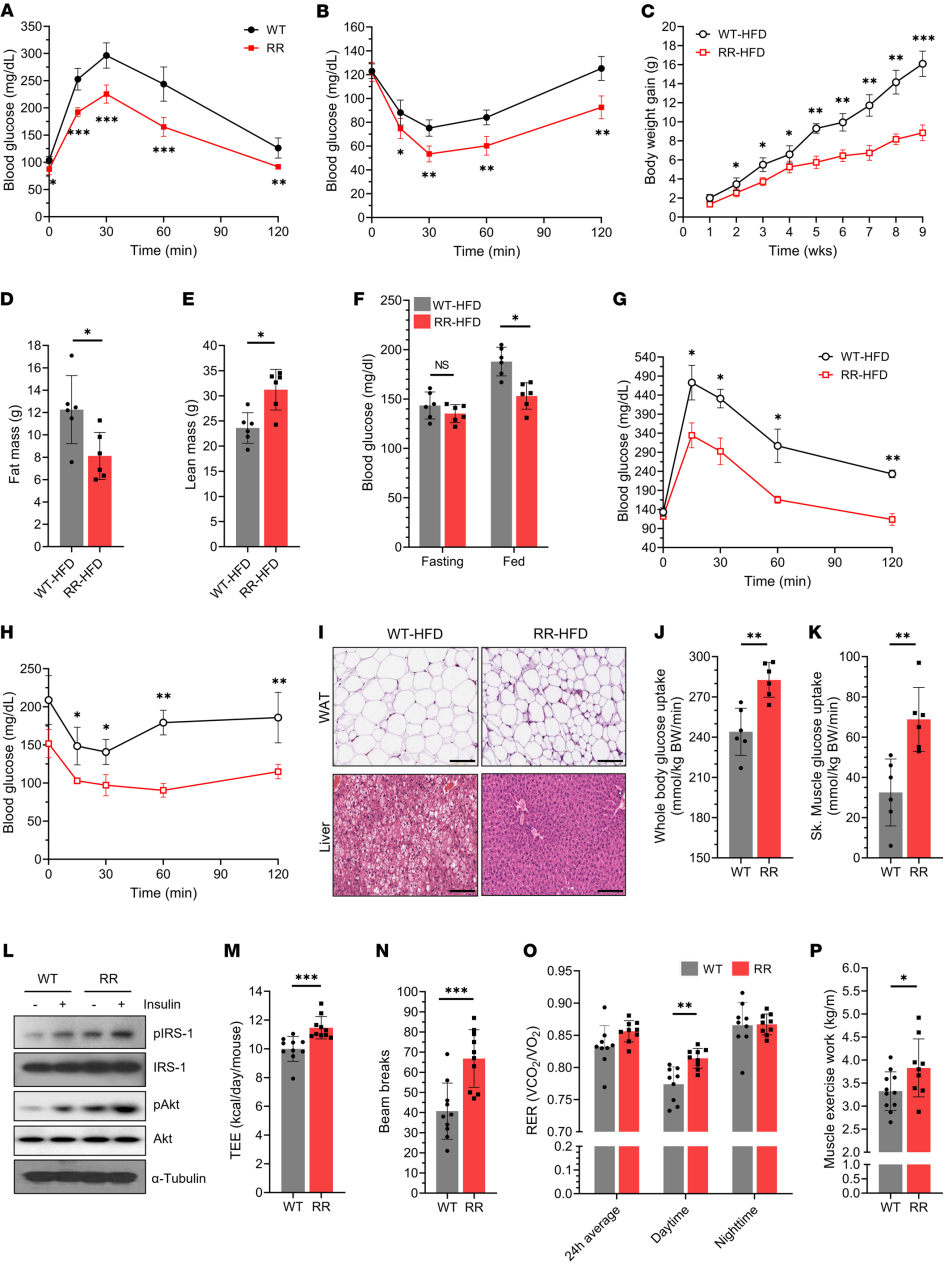
Improved whole-body metabolism in *Cdk4*^R/R^ mice. (**A**) Glucose tolerance and (**B**) insulin sensitivity in *Cdk4*^R/R^ mice compared with those in *Cdk4*^WT^ mice. (**C**) Body weight, (**D**) fat mass, (**E**) lean mass, (**F**) fasting and fed blood glucose levels, (**G**) glucose tolerance, and (**H**) insulin sensitivity in *Cdk4*^R/R^ mice and *Cdk4*^WT^ mice in response to 60% HFD. (**I**) Histology of white adipose (WAT) (top) and liver tissues (bottom) from HFD-fed *Cdk4*^R/R^ mice and similarly fed *Cdk4*^WT^ mice. Scale bars: 100 μm. (**J**) Whole-body and (**K**) skeletal muscle glucose uptake during hyperinsulinemic-euglycemic clamp assay in regular chow–fed *Cdk4*^R/R^ mice compared with *Cdk4*^WT^ mice. (**L**) Phosphorylation levels of insulin signaling pathway intermediatory proteins, IRS-1 and Akt, in *Cdk4*^R/R^ muscle in comparison to *Cdk4*^WT^ muscle. Total IRS-1,Akt, and α-tubulin proteins are shown as controls. (**M**) Energy expenditure, (**N**) ambulatory activity, (**O**) RER, and (**P**) muscle exercise work in *Cdk4*^R/R^ mice and *Cdk4*^WT^ mice (*n* = 9–11 mice each group). Between 6 and 8 mice per group fed regular chow were used in each experiment, unless mentioned otherwise. Data are shown as the mean ± SEM. *P < 0.05, **P < 0.01, ***P < 0.001 by 2-tailed Student’s *t* test.

We used Cdk4-deficient (*Cdk4*^neo/neo^, referred here as *Cdk4*^KO^) mice and constitutively active *Cdk4*^R24C/R24C^ (*Cdk4*^R/R^) mice (31). *Cdk4*^R/R^ mice harbor the arginine to cysteine point mutation at the 24th amino acid position on the Cdk4 protein (hence the term Cdk4^R24C^). This mutation renders the Cdk4^R24C^ kinase insensitive to inhibition by p16^Ink4a^ — a product of the *CDKN2A* locus (39, 40). *Cdk4*^R/R^ mice were longer and heavier (Supplemental Figure 1, A and B; supplemental material available online with this article; https://doi.org/10.1172/JCI162479DS1), with a significant increase in skeletal muscle mass. When fed regular chow diet (RD), the mice exhibited lower fasting glucose levels (Supplemental Figure 1C), with no significant differences in fed glucose and insulin levels (Supplemental Figure 1, D and E). Food intake normalized to body weight was comparable to that seen in controls (Supplemental Figure 1F). In response to RD feeding, *Cdk4*^R/R^ mice exhibited improved glucose tolerance (Figure 1A and Supplemental Figure 1G), enhanced insulin sensitivity (Figure 1B and Supplemental Figure 1H), reduced serum triglycerides and fatty acid levels, and increased adiponectin levels (Supplemental Figure 1, I–K).

High-fat diet–fed (HFD-fed) *Cdk4*^R/R^ mice maintained lower body weight and reduced fat mass (Figure 1, C and D). Food intake normalized to body weight was significantly lower in *Cdk*4^R/R^ mice during the first week of exposure to HFD compared with that of WT Cdk4 mice fed HFD (Supplemental Figure 2A). HFD-fed *Cdk4*^R/R^ mice demonstrated increased ambulatory activity during weeks 5 and 6 of HFD feeding (Supplemental Figure 2B). These data suggest that lower food intake and increased ambulatory activity may, at least partially, explain the reduced weight gain in HFD-fed *Cdk4*^R/R^ mice. HFD-fed *Cdk4*^R/R^ mice exhibited significantly increased lean mass (Figure 1E), reduced fed blood glucose levels (Figure 1F), and decreased serum insulin levels (Supplemental Figure 2C). Moreover, these mice showed enhanced glucose tolerance (Figure 1G and Supplemental Figure 2D) and improved insulin sensitivity (Figure 1H and Supplemental Figure 2E). In addition, decreased ectopic fat accumulation was seen in the liver, muscle, and white adipose tissues (Figure 1I and Supplemental Figure 2F), suggesting that *Cdk4*^R/R^ mice were protected from HFD-induced steatosis. Consistent with these results, skeletal muscle of HFD-fed *Cdk4*^R/R^ mice showed reduced expression of lipogenic genes with concomitant elevation of fatty acid oxidation genes and mitochondrial markers (Supplemental Figure 2G). Reduced levels of serum triglyceride and free fatty acids were also observed (Supplemental Figure 3, A and B). Furthermore, serum levels of detrimental adipocytokines resistin and IL-6 were reduced, while the level of the beneficial adipokine adiponectin was increased (Supplemental Figure 3, C and E). No significant changes were detected in the circulating levels of leptin or the inflammatory cytokines TNF-α, PAI-1, and MCP-1 (Supplemental Figure 3, F–I). Taken together, these results are consistent with improved glucose homeostasis in *Cdk4*^R/R^ mice.

RD-fed *Cdk4*^R/R^ mice exhibited significantly higher whole-body glucose uptake, specifically in skeletal muscle (Figure 1, J and K), but not in white (WAT) or brown (BAT) adipose tissues (Supplemental Figure 3, J and K) compared with RD-fed *Cdk4*^WT^ mice. Enhanced phosphorylation insulin receptor signaling intermediatories, IRS-1 and Akt, were further suggestive of increased insulin sensitivity in *Cdk4*^R/R^ muscles (Figure 1L). Additionally, we observed significantly reduced triglyceride accumulation in *Cdk4*^R/R^ muscles (Supplemental Figure 3L). These findings are evidence of improved glucose uptake by *Cdk4*^R/R^ skeletal muscle. Furthermore, indirect calorimetry revealed that RD-fed *Cdk4*^R/R^ mice exhibit enhanced total energy expenditure (Figure 1M) and oxygen consumption (Supplemental Figure 4A), suggesting that *Cdk4*^R/R^ mice have a higher metabolic rate. *Cdk4*^R/R^ mice also displayed increased ambulatory activity (Figure 1N), although we did not observe significant changes in total physical activity (which measures ambulatory plus nonambulatory activity) or food intake (Supplemental Figure 4, B–F). We also examined the respiratory exchange ratio (RER), which reflects whole-body fuel oxidation. Chow-fed *Cdk4*^R/R^ mice showed significantly increased RER during the daytime (Figure 1O), suggesting relatively higher utilization of carbohydrates. In vivo ^14^C-oleic oxidation was comparable in *Cdk4*^WT^ and *Cdk4*^R/R^ mice (Supplemental Figure 4G). Furthermore, *Cdk4*^R/R^ mice exhibited increased muscle exercise capacity (Figure 1P) and greater grip strength (Supplemental Figure 4H). Expression levels of metabolic genes (*Pgc-1*α*, AMPKs*) and mitochondrial genes (*Ndusf2, Uqcrc2, Tfam* and *Atp5a1*) were elevated in muscles, but not liver, WAT and BAT from *Cdk4*^R/R^ mice (Supplemental Figure 5, A–G). Taken together, these data show that *Cdk4*^R/R^ mice have improved muscle function with respect to glucose metabolism, physical strength, and work capacity.

### Cdk4 drives expansion of slow/oxidative muscle fibers.

**Figure 2:**
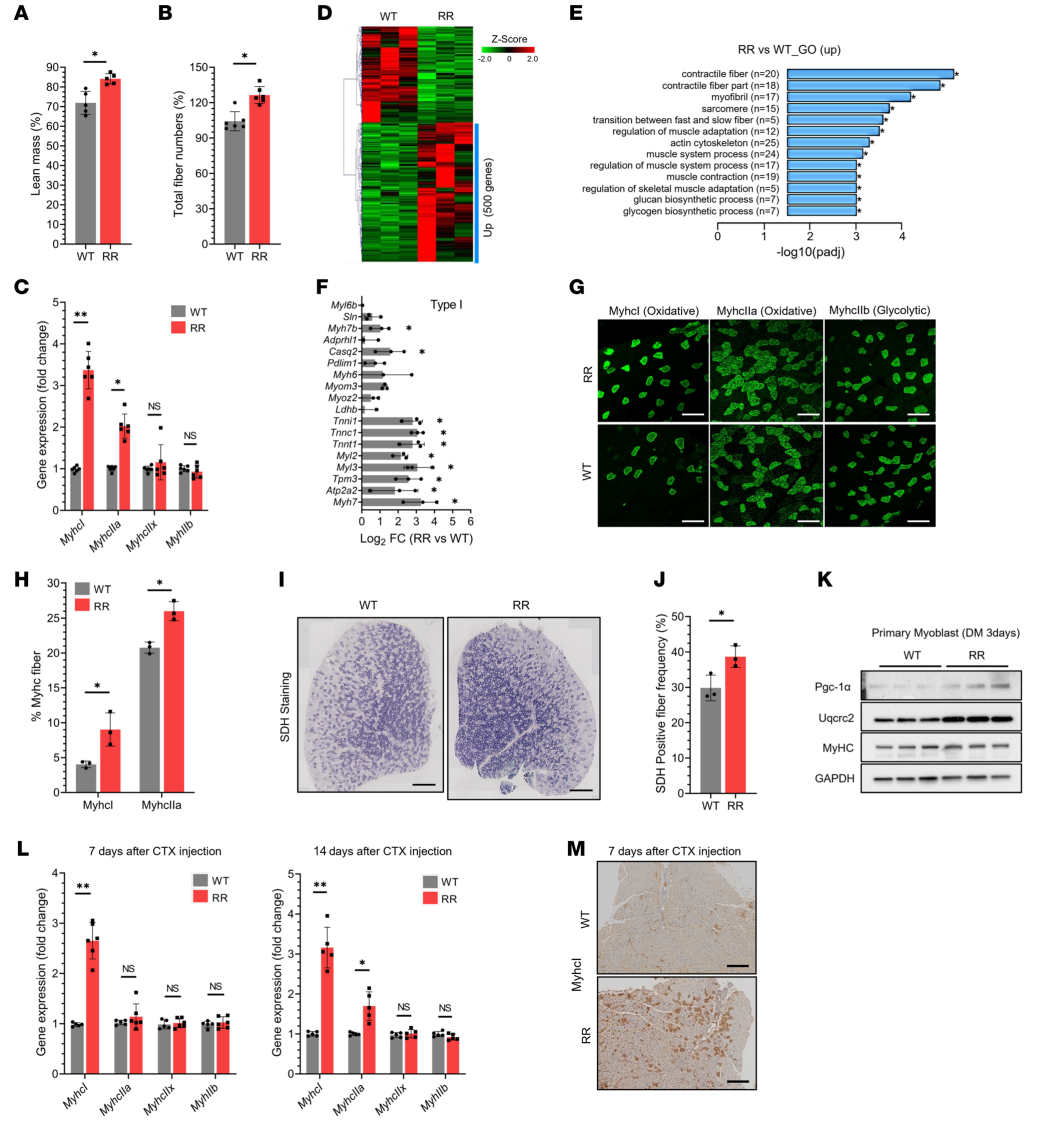
*Cdk4*^R/R^ muscles display increased oxidative fibers without change in glycolytic fiber composition. (**A**) Lean mass and (**B**) total muscle fiber numbers in *Cdk4*^R/R^ mice compared with those in *Cdk4*^WT^ mice. (**C**) mRNA levels of myosin isoforms in quadriceps (QA) muscles of *Cdk4*^WT^ and *Cdk4*^R/R^ mice (*n* = 6 mice each group). (**D**) Heatmap of RNA-Seq analysis of QA muscle transcripts in *Cdk4*^WT^ and *Cdk4*^R/R^ mice (*n* = 3 mice each group). Upregulated (Up) mRNAs are indicated with a blue bar. (**E**) Gene ontology (GO) analysis of upregulated genes in QA muscles of *Cdk4*^WT^ and *Cdk4*^R/R^ mice. (**F**) Log_2_ fold change RNA-Seq values of type I fiber markers in QA muscles of *Cdk4*^WT^ and *Cdk4*^R/R^ mice. (**G**) Immunofluorescence and (**H**) quantification of slow/oxidative fibers (MyhcI and MyhcIIa) and fast/glycolytic fibers (MyhcIIx and MyhcIIb) in QA muscles of *Cdk4*^WT^ and *Cdk4*^R/R^ mice. Scale bars: 200 μm. (**I**) Histochemistry staining and (**J**) quantification of metabolically active SDH-positive fibers in TA muscles of *Cdk4*^WT^ and *Cdk4*^R/R^ mice (*n* = 5 mice each group). Scale bars: 500 μm. (**K**) Expression of MyHC, Pgc-1α and Uqcrc2 proteins in differentiated primary myoblasts from TA muscles of *Cdk4*^WT^ and *Cdk4*^R/R^ mice (*n* = 3 mice each group). GAPDH protein is shown as loading control. (**L**) mRNA expression levels of slow/oxidative muscle-specific transcripts (*MyhcI* and *MyhcIIa*) and fast/glycolytic muscle transcripts (*MyhcIIx* and *MyhcIIb*) in TA muscles of *Cdk4*^WT^ and *Cdk4*^R/R^ mice at 7 and 14 days after CTX injection (*n* = 5–6 mice each group). (**M**) Immunostaining of slow/oxidative muscle fibers (MyhcI positive) 7 days after CTX injection in TA muscles of *Cdk4*^WT^ and *Cdk4*^R/R^ mice (*n* = 5 mice each group). Scale bars: 500 μm. Between 5 and 6 mice per group were used in each experiment, unless mentioned otherwise. Data are shown as the mean ± SEM. *P < 0.05, *P < 0.01 by 2-tailed Student’s *t* test.

*Cdk4*^R/R^ mice were leaner (Figure 2A) with increased muscle fiber numbers (Figure 2B) compared with *Cdk*^WT^ mice. Differentiated muscle fibers are categorized as slow-twitch oxidative, fast-twitch oxidative-glycolytic, or fast-twitch glycolytic fibers (13). These myofibers are categorized based on differential gene expression of *Myhc* isoforms, and most mammalian muscles contain a mixture of fiber types. For example, the soleus muscles have a higher proportion of mitochondria-rich type I (MyhcI) and IIA (MyhcIIa) fibers; in contrast, muscles like the extensor digitorum longus (EDL) and quadriceps contain more of the faster type IIB (MyhcIIb) fibers that have lower mitochondrial numbers (41). mRNA expression of *MyhcI* and *MyhcIIa* was significantly increased in *Cdk4*^R/R^ quadriceps muscles, with no change in *MyhcIIb* mRNA expression (Figure 2C), compared with *Cdk4*^WT^ quadriceps muscles. RNA-Seq analyses further demonstrated a preferential increase in markers associated with type 1 and IIA fibers, along with a modest reduction in type IIB fiber markers (Figure 2, D–F, and Supplemental Figure 6, A–C). In agreement, immunofluorescence assays revealed increased proportions of MyhcI- and MyhcIIa-positive oxidative/slow myofibers in *Cdk4*^R/R^ muscles, with no change in MyhcIIb-positive glycolytic/fast fiber content (Figure 2, G and H). Furthermore, significant enrichment of metabolically active succinate dehydrogenase–positive (SDH-positive) fibers was observed in the tibialis anterior (TA) muscles of *Cdk4*^R/R^ mice (Figure 2, I and J).

Levels of myogenic differentiation were similar in primary myoblasts from *Cdk4*^R/R^ and *Cdk4*^WT^ mice (Supplemental Figure 7, A and B); however, levels of mitochondrial markers Pgc-1α and Uqcrc2 were increased in differentiated primary myoblasts from *Cdk4*^R/R^ mice (Figure 2K and Supplemental Figure 7, C and D). Furthermore, pharmacological inhibition of Cdk4 in differentiated C2C12 myotubes suppressed expression of *Pgc-1*α and *Tfam* and levels of slow/oxidative muscle fiber markers (*MyhcI, MyhcIIa*), with no change in fast/glycolytic specific markers (*MyhcIIx, MyhcIIb*) (Supplemental Figure 8, A–D).

### Cdk4 promotes regeneration of oxidative muscle fibers.

The above observations are consistent with the notion that Cdk4 preferentially promotes the expansion of oxidative muscle fibers. To test this hypothesis, we utilized a cardiotoxin-induced (CTX-induced) muscle injury model (42). CTX was injected into TA muscle after which mice were allowed unrestricted access to drinking water containing bromodeoxyuridine analogs for 2 weeks. Increased Cdk4 expression was observed in CTX-injected muscle of WT mice 7 days after injury (Supplemental Figure 9A). Cdk4 protein was present in either the perinuclear or intranuclear regions of most central nuclei of regenerating fibers (Supplemental Figure 9, B–J). *Cdk4*^R/R^ muscle exhibited higher cell proliferation (Supplemental Figure 9K). Consistent with the primary myogenic differentiation data (Supplemental Figure 7, A and B), the extent of muscle regeneration was similar in *Cdk4*^R/R^ and *Cdk4*^WT^ mice (Supplemental Figure 9, L and M). Notably, gene expression levels of slow/oxidative type Myhc genes (*MyhcI* and *MyhcIIa*) were significantly increased at 7 and 14 days after CTX injection in *Cdk4*^R/R^ mice, without any change in fast/glycolytic marker gene expression (Figure 2L). In agreement, immunostaining showed that MyhcI-positive slow/oxidative myofibers were increased in regenerating *Cdk4*^R/R^ muscles 7 days after CTX injection (Figure 2M). These observations demonstrate that Cdk4 preferentially promotes regeneration of slow/oxidative skeletal muscle fibers.

### Cdk4 enhances muscle mitochondrial biogenesis and bioenergetics.

**Figure 3:**
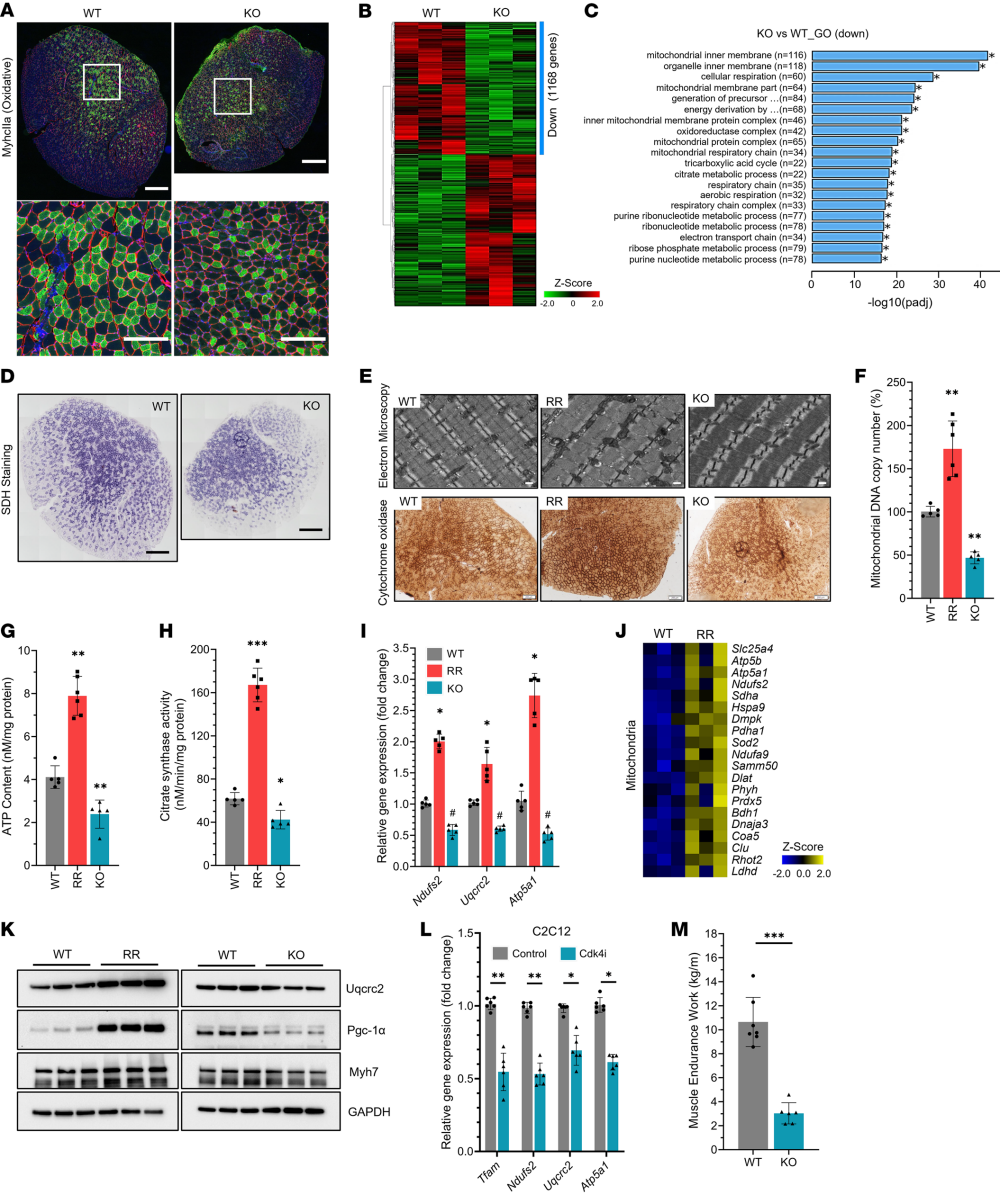
Mitochondrial phenotype in *Cdk4*^R/R^ and *Cdk4*^KO^ muscle. (**A**) Immunofluorescence showing MyhcIIa expression in TA muscles of *Cdk4*^WT^ and *Cdk4*^KO^ mice (*n* = 5 mice each group). MyhcIIa (green), laminin (red), DAPI (blue). Enlarged images of the areas in the white squares are shown below. Scale bars: 500 μm (top); 200 μm (bottom). (**B**) RNA-Seq heatmap of soleus muscle transcripts in *Cdk4*^WT^ and *Cdk4*^KO^ mice (*n* = 3 mice each group). Downregulated (Down) mRNAs are indicated with a blue bar. (**C**) Gene ontology (GO) analysis of downregulated genes in *Cdk4*^WT^ and *Cdk4*^KO^ muscle. (**D**) Histochemical analyses of metabolically active SDH-positive fibers in TA muscles of *Cdk4*^WT^ and *Cdk4*^KO^ mice (*n* = 5 mice each group). Scale bars: 500 μm. (**E**) Representative electron microscopy images showing mitochondria in *Cdk4*^WT^, *Cdk4*^R/R^, and *Cdk4*^KO^ muscle (top). Scale bars: 500 nm. Immunostaining showing cytochrome oxidase–positive fibers in *Cdk4*^WT^, *Cdk4*^R/R^, and *Cdk4*^KO^ muscle (bottom) (*n* = 4–5 mice each group). Scale bars: 200 μm. (**F**) Mitochondrial DNA copy numbers, (**G**) ATP content, and (**H**) citrate synthase activity in QA muscles of *Cdk4*^WT^, *Cdk4*^R/R^ and *Cdk4*^KO^ mice. (**I**) mRNA levels of mitochondria markers (*Ndufs2*,*Uqcrc2* and *Atp5a1*) in QA muscles from *Cdk4*^WT^, *Cdk4*^R/R^, and *Cdk4*^KO^ mice (*n* = 5–6 mice each group). (**J**) Heatmap of mitochondrial transcripts in *Cdk4*^WT^ and *Cdk4*^R/R^ muscle (*n* = 3 mice each group). (**K**) Protein expression of Pgc-1α, Uqcrc2, and Myh7 in TA muscles of *Cdk4*^WT^, *Cdk4*^R/R^, and *Cdk4*^KO^ mice (*n* = 3 mice each group). (**L**) Effects of Cdk4 inhibitor (IDCX) on of mitochondrial genes *Tfam, Ndufs2*, *Atp5a1*, and *Uqcrc2* in C2C12 myotubes. (**M**) Muscle endurance in *Cdk4*^WT^ and *Cdk4*^KO^ mice. Between 6 and 8 mice per group were used in each experiment, unless mentioned otherwise. Data are shown as the mean ± SEM. *P < 0.05, **P < 0.01, ***P < 0.001, ^#^*P* < 0.05 vs WT, by 2-tailed Student’s *t* test.

*Cdk4*^KO^ mice exhibited severe reductions in MyhcIIa-positive oxidative myofibers and markers of type 1 fibers (Figure 3A and Supplemental Figure 10A). Muscle function is largely dependent on mitochondrial reserves and the mitochondrial activity resident in muscle cells (43, 44). RNA-Seq analyses demonstrated significant reductions in *Cdk4*^KO^ muscle mitochondrial gene expression (Figure 3, B and C), suggestive of defects in mitochondrial biogenesis and/or function. In agreement with this, *Cdk4*^KO^ mice showed reduced SDH-positive fibers in their TA muscles (Figure 3D). Electron microscopy revealed increased mitochondria in *Cdk4*^R/R^ muscle, whereas *Cdk4*^KO^ muscle exhibited depleted mitochondria (Figure 3E, top). Furthermore, robust cytochrome oxidase staining was observed in the *Cdk4*^R/R^ muscle, in contrast to severely reduced cytochrome oxidase staining in the *Cdk4*^KO^ muscle tissue (Figure 3E, bottom). Consistent with enhanced mitochondrial biogenesis and function in *Cdk4*^R/R^ muscle, significant increases in mitochondrial DNA copy number and mitochondrial area were observed in the quadriceps muscle and the EDL muscle (Figure 3F and Supplemental Figure 10, B–E). In agreement with this, increased ATP content (Figure 3G) and citrate synthase activity (Figure 3H) were detected in *Cdk4*^R/R^ muscle along with elevated expression of mitochondrial genes (Figure 3, I and J). In contrast, the above features were significantly impaired in muscle tissue from *Cdk4*^KO^ mice, suggesting a defective mitochondrial phenotype in these mice (Figure 3, F–I, and Supplemental Figure 10, B–E). Furthermore, levels of mitochondrial proteins Uqcrc2 and Pgc-1α were higher in *Cdk4*^R/R^ muscle and decreased in *Cdk4*^KO^ muscle (Figure 3K). In addition, pharmacological inhibition of Cdk4 in differentiating C2C12 cells significantly reduced expression levels of mitochondrial markers (Figure 3L) and levels of oxygen consumption (Supplemental Figure 10F), suggesting functional impairment of these cells upon inhibition of Cdk4 activity. *Cdk4*^KO^ mice also exhibited significantly lower muscle endurance capacity (Figure 3M) and were limited in their ability to run for an extended time and distance compared with *Cdk4*^WT^ mice (Supplemental Figure 10, G and H). Taken together, these findings show that Cdk4 is essential for optimal numbers of slow/oxidative muscle fibers and normal muscle mitochondrial biogenesis and function.

### Cdk4-E2F3–dependent increase in PGC-1α expression.

**Figure 4:**
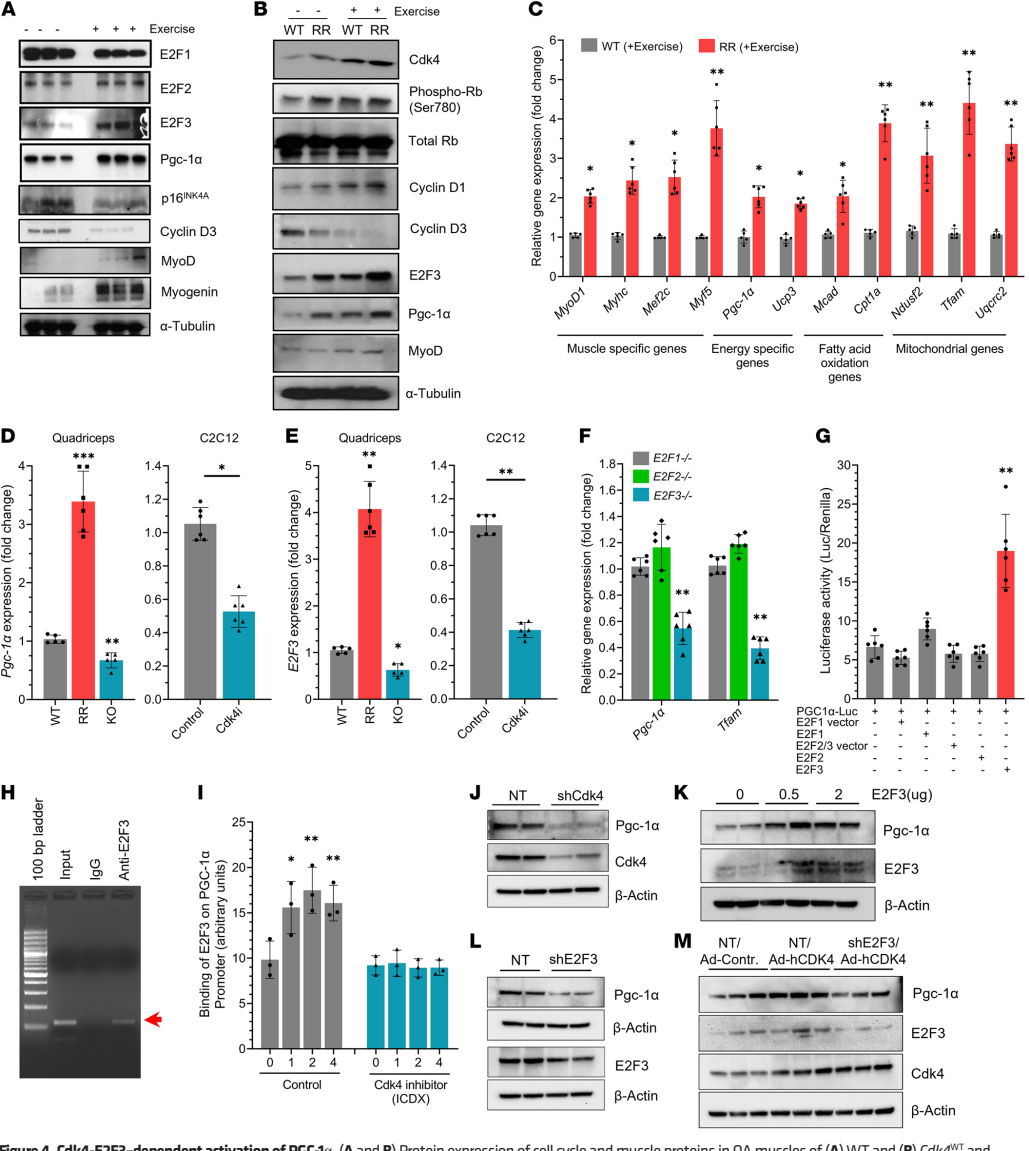
Cdk4-E2F3–dependent activation of PGC-1α. (**A** and **B**) Protein expression of cell cycle and muscle proteins in QA muscles of (**A**) WT and (**B**) *Cdk4*^WT^ and *Cdk4*^R/R^ mice with (+) or without (–) exercise (*n* = 3 mice each group). (**C**) Gene expression of indicated genes in QA muscles from *Cdk4*^WT^ and *Cdk4*^R/R^ mice after exercise (*n* = 5–6 mice each group). (**D**) *Pgc-1*α and (**E**) *E2F3* mRNA levels in QA muscles of *Cdk4*^WT^, *Cdk4*^R/R^, and *Cdk4*^KO^ mice and upon Cdk4i inhibition in differentiating C2C12 myotubes (*n* = 6 each group). (**F**) mRNA transcripts of *Pgc-1*α and *Tfam* in *E2F1*^–/–^, *E2F2*^–/–^, and *E2F3*^–/–^ MEFs (*n* = 6 each group). (**G**) PGC-1α reporter (PGC-1α-Luc) activity upon overexpression of E2F1, E2F2, E2F3, and vector control (*n* = 6 each group). (**H**) ChIP-assay showing E2F3 binding to the PGC-1α promoter compared with control IgG antibody. Red arrow points to the amplified PGC-1α promoter region. (**I**) Real-time qPCR data shows binding of E2F3 to the PGC-1α promoter during the 4-day muscle differentiation program of C2C12 cells with and without (control) the presence of a Cdk4i inhibitor (*n* = 3 each group). (**J** and **K**) Pgc-1α protein expression in differentiated C2C12 myotubes upon shRNA knockdown of (**J**) Cdk4 or (**K**) E2F3 as compared with nontarget shRNA control (NT). (**L**) Levels of E2F3 and Pgc-1α in differentiating C2C12 cells without (0 μg) or with (0.5 or 2 μg) pCMV-E2F3–HA–induced expression of exogenous E2F3. (**M**) Western blot analysis of Pgc-1α protein upon shRNA knockdown of E2F3 (shE2F3), compared with nontarget shRNA control (NT) treatment, with or without adenovirally overexpressed human Cdk4 (Ad-hCDK4) in differentiated C2C12 myotubes. E2F3, Cdk4, and β-actin protein levels are shown as controls. Data are shown as the mean ± SEM. *P < 0.05, **P < 0.01, ***P < 0.001 by 2-tailed Student’s *t* test.

As expected, levels of *MyoD1* and *Pgc-1*α were increased in exercised WT muscle. Furthermore, levels of energy sensing, fatty acid oxidation, and mitochondrial genes increased with exercise in WT mice (Supplemental Figure 11). Cdk4 targets the E2F family of transcription factors that in turn regulate cell-type-specific gene expression (25, 45). E2F3 levels were increased, whereas p16^Ink4a^ levels were reduced in skeletal muscle from exercised WT mice (Figure 4A). p16^Ink4a^ protein levels were reduced in *Cdk4*^R/R^ myoblasts (Supplemental Figure 12, A and B). Furthermore, increased phosphorylated forms of RB were detected in *Cdk4*^R/R^ myoblasts (Supplemental Figure 12, A and B). Consistent with these findings, phosphorylation levels of RB^serine^ ^780^ were increased in *Cdk4*^R/R^ muscle (Figure 4B). Taken together, these observations are indicative of overall increased Cdk4^R24C^ activity in *Cdk4*^R/R^ skeletal muscle.

Cyclin D3 is implicated in muscle development (46–49), and its levels were reduced in response to exercise (Figure 4A). Levels of cyclin D1 were increased and those of cyclin D3 reduced in *Cdk4*^R/R^ muscle (Figure 4B). Levels of E2F3 and Pgc-1α were also increased in sedentary *Cdk4*^R/R^ muscle, and those levels were further elevated upon exercise (Figure 4B), along with a coordinate increase in markers associated with improved muscle function, enhanced energy sensing, fatty acid oxidation, and mitochondrial function (Figure 4C). In addition, *Pgc-1*α and *E2F3* transcripts were increased in *Cdk4*^R/R^ quadriceps muscle but were suppressed in *Cdk4*^KO^ muscles and 2-Bromo-12,13-dihydro-5H-indolo(2,3-a) pyrrolo (3,4)carbazole–treated **(PRODUCTION EDITOR: The “(3,4” here isn’t a reference.)** (IDCX-treated) C2C12 cells during muscle differentiation (Figure 4, D and E). Taken together, these results suggest a positive association of Cdk4-E2F3 signals with that of PGC-1α expression, which we examined next.

A selective and marked increase in *E2F3* transcripts, but not that of *E2F1* and *E2F2,* was seen during differentiation of C2C12 cells (Supplemental Figure 13A). In agreement, E2F3 protein levels were also increased along with those of Myhc and Pgc-1α (Supplemental Figure 13B). Expression of *Pgc-1*α and *Tfam* transcripts was significantly suppressed in *E2F3*^–/–^ MEFs but not *E2F1*^–/–^ or *E2F2*^–/–^ MEFs (Figure 4F). Importantly, E2F3, but not E2F1 or E2F2, activated the PGC-1α-luciferase reporter (Figure 4G). Moreover, ChIP assays using C2C12 cells undergoing muscle differentiation revealed a robust binding of E2F3 to the PGC-1α promoter (Figure 4H and Supplemental Figure 13C) where we observed a time-dependent increase in E2F3 binding to the PGC-1α promoter (Figure 4I). Furthermore, addition of Cdk4 inhibitor, IDCX, eliminated E2F3’s association with the PGC-1α promoter (Figure 4I and Supplemental Figure 13, D and E), suggesting that this binding is Cdk4 dependent.

Knockdown of Cdk4 or E2F3 or addition of IDCX reduced the levels of Pgc-1α in differentiated C2C12 myoblasts (Figure 4, J and K, and Supplemental Figure 13F). Overexpression of Cdk4 in differentiated C2C12 myoblasts resulted in an E2F3-dependent increase in levels of Pgc-1α protein (Figure 4, L and M). In contrast, E2F3 knockdown suppressed the levels of Pgc-1α in Cdk4-overexpressing C2C12 myoblasts (Figure 4M). All together, these results suggest that Cdk4-E2F3 promotes activation of PGC-1α, a master regulator of mitochondrial biogenesis and energy homeostasis.

### Muscle-specific E2F3-deficient mice are susceptible to HFD-induced obesity and diabetes.

**Figure 5:**
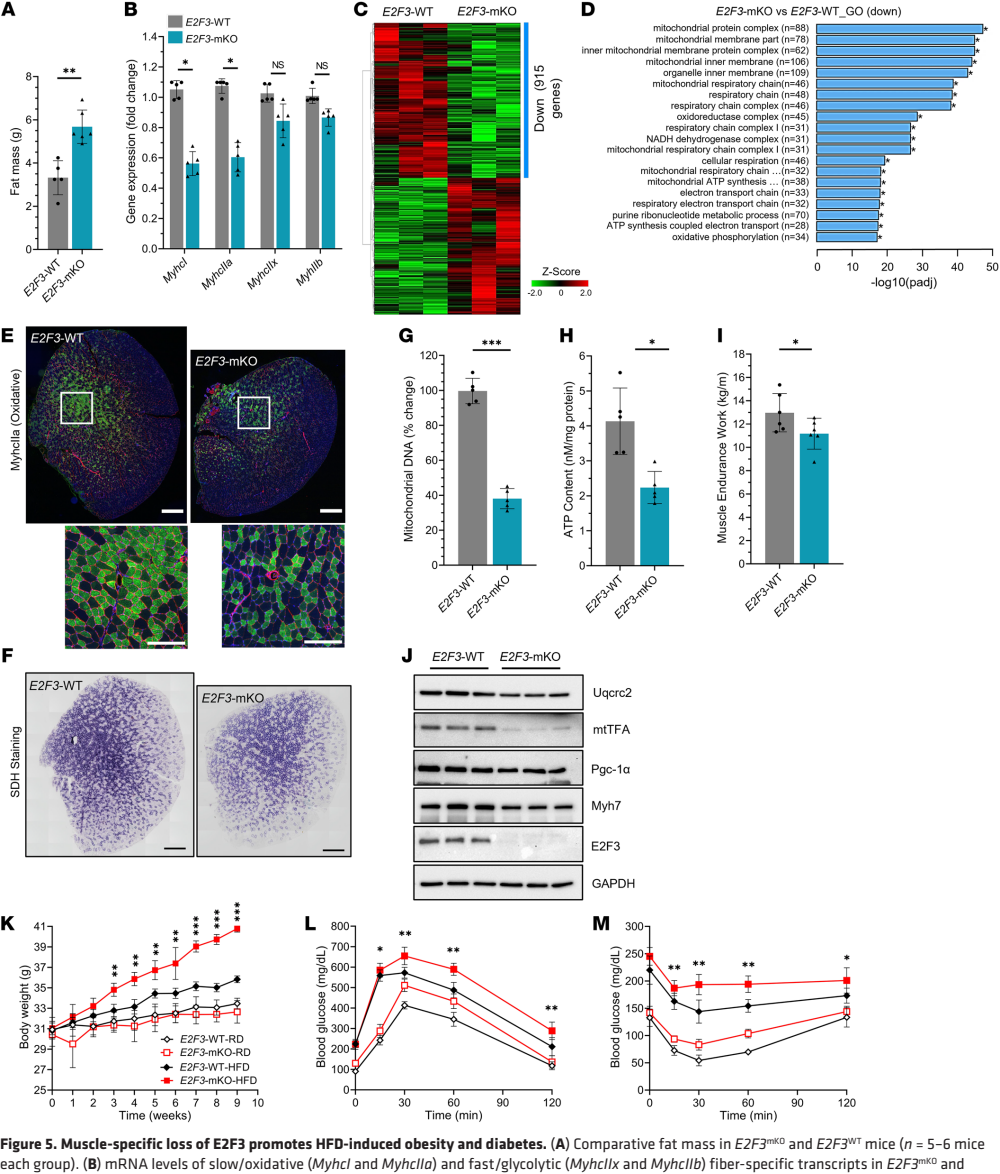
Muscle-specific loss of E2F3 promotes HFD-induced obesity and diabetes. (**A**) Comparative fat mass in *E2F3*^mKO^ and *E2F3*^WT^ mice (*n* = 5–6 mice each group). (**B**) mRNA levels of slow/oxidative (*MyhcI* and *MyhcIIa*) and fast/glycolytic (*MyhcIIx* and *MyhcIIb*) fiber-specific transcripts in *E2F3*^mKO^ and *E2F3*^WT^ QA muscles (*n* = 5 mice each group). (**C**) Heatmap of RNA-Seq analysis comparing soleus muscle transcripts (*P* < 0.05) between *E2F3*^mKO^ and *E2F3*^WT^ mice (*n* = 3 mice per group). Downregulated (Down) mRNAs are indicated with a blue bar. (**D**) Gene ontology (GO) analysis of downregulated genes between *E2F3*^mKO^ and *E2F3*^WT^ muscles. (**E**) Representative immunofluorescence of MyhcIIa protein in *E2F3*^mKO^ and *E2F3*^WT^ TA muscles (*n* = 5 mice each group). MyhcIIa (green), laminin (red),DAPI (blue). Enlarged images of the areas in the white squares are shown below. Scale bars: 500 μm (top); 200 μm (bottom). (**F**) Representative histochemical staining of active SDH-positive fibers in TA muscles of *E2F3*^mKO^ and *E2F3*^WT^ mice (*n* = 5 mice each group). Scale bars: 500 μm. (**G**) Mitochondrial DNA content and (**H**) ATP content in *E2F3*^mKO^ and *E2F3*^WT^ muscle. (**I**) Muscle endurance capacity in *E2F3*^mKO^ and *E2F3*^WT^ mice. (**J**) Protein expression in *E2F3*^mKO^ and *E2F3*^WT^ TA muscles (*n* = 3 mice each group). (**K**) Body weight, (**L**) glucose tolerance, and (**M**) insulin sensitivity in *E2F3*^mKO^ and *E2F3*^WT^ mice fed regular diet (RD; open symbols) and high-fat diet (HFD; closed symbols). Between 5 and 8 mice per group were used in each experiment, unless mentioned otherwise. *P < 0.05, **P < 0.01, ***P < 0.001 by 2-tailed Student’s *t* test.

To investigate E2F3’s role in muscle, we generated muscle-specific E2F3 conditional knockout (*E2F3*^mKO^) mice. *E2F3*^fl/fl^ mice (50, 51) were bred with mice expressing Cre recombinase under a myosin light chain 1/3 promoter/enhancer (MLC-Cre mice), wherein the Cre expression is restricted to skeletal muscle (52). *E2F3*^mKO^ mice were born at the expected Mendelian ratio without significant changes in their body weight and lean mass (Supplemental Figure 14, A and B). However, by 3–4 months of age *E2F3*^mKO^ mice displayed significantly increased fat mass (Figure 5A). *E2F3*^mKO^ muscle exhibited decreased expression of slow/oxidative muscle fiber–specific genes (*MyhcI* and *MyhcIIa*), *Myf5, Mef2c, MyoD1*, *Pgc-1*α, and muscle/mitochondrial markers (Figure 5B and Supplemental Figure 14, C and D). Similar to *Cdk4*^KO^ muscle (Figure 3, B and C), RNA-Seq analyses revealed significant reductions in *E2F3*^mKO^ muscle mitochondrial gene expression (Figure 5, C and D, and Supplemental Figure 14D), suggestive of defects in mitochondrial metabolism. *E2F3*^mKO^ mice exhibited markedly decreased muscle size, MyhcIIa-positive oxidative myofibers, and SDH-positive fibers in TA muscle (Figure 5, E and F). Furthermore, *E2F3*^mKO^ muscle exhibited significantly reduced mitochondrial DNA copy numbers (Figure 5G) and ATP content (Figure 5H). Levels of mitochondrial proteins Uqcrc2, mtTFA, and Pgc-1α decreased in *E2F3*^mKO^ muscle (Figure 5J).

Consistent with diminished muscle function, *E2F3*^mKO^ mice exhibited significantly lower muscle endurance capacity (Figure 5I) and were limited in their ability to run for an extended time and distance (Supplemental Figure 14, E and F). In addition, we observed elevated lipogenic gene expression in *E2F3*^mKO^ muscles (Supplemental Figure 14G). Furthermore, HFD feeding increased the susceptibility of *E2F3*^mKO^ mice to body weight gain, fat mass expansion, glucose intolerance, and insulin resistance (Figure 5, K–M, and Supplemental Figure 14, H–J). Overall, these results demonstrate that skeletal muscle–specific loss of *E2F3* results in mitochondrial defects that contribute to metabolic disease.

### Cdk4-E2F3 signals regulate oxidative muscle fibers and mitochondrial function.

**Figure 6:**
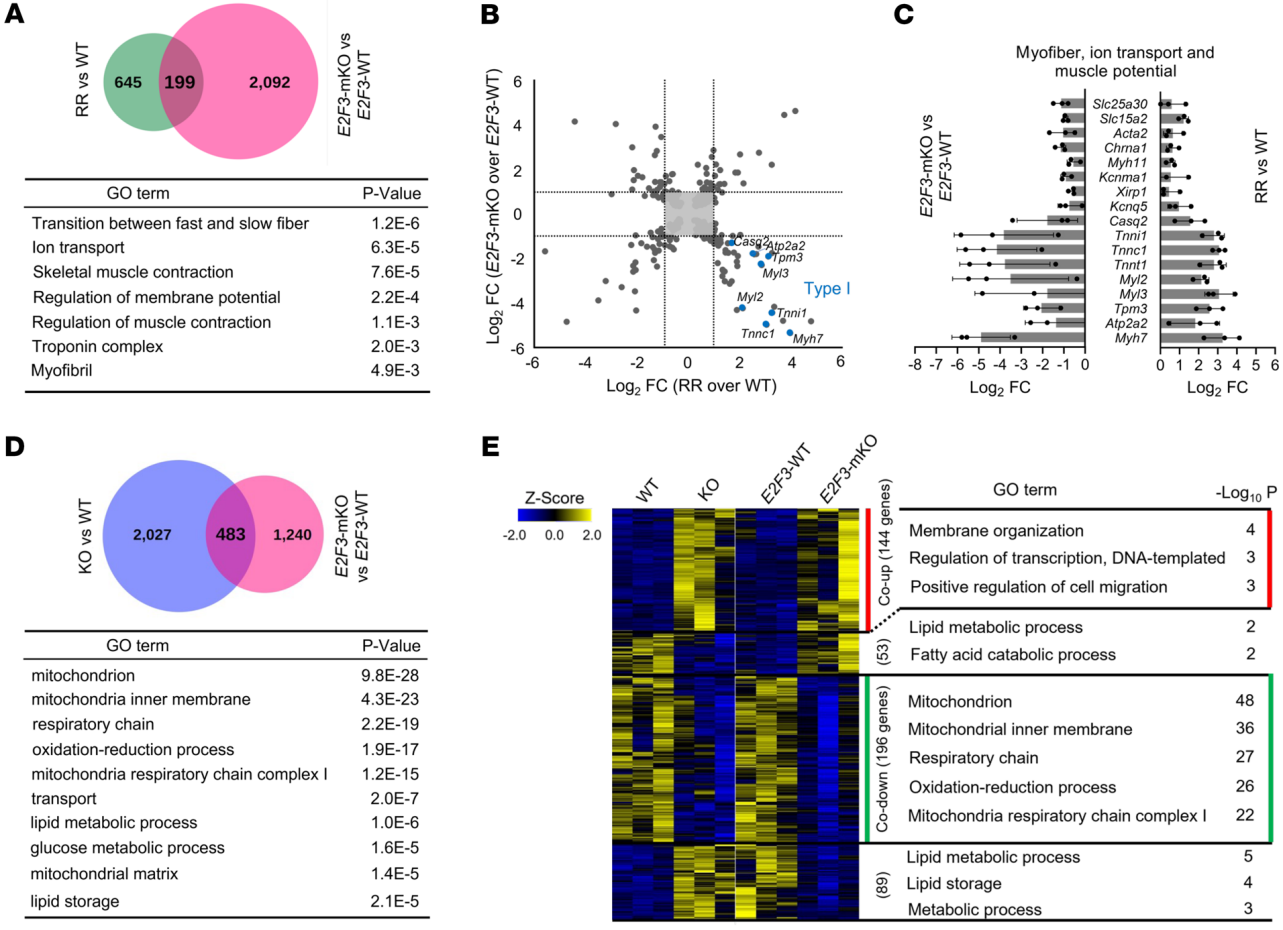
Correlation of myofiber and mitochondrial gene expression with Cdk4 and E2F3 status. (**A**) Venn diagram showing the overlap of differentially expressed genes (DEGs; *P* < 0.05) comparing both Cdk4^WT^ (WT) and Cdk4^R/R^ (RR) mice as well as E2F3^WT^ and E2F3^mKO^ mice (top), and GO analysis of 199 overlapping genes (bottom). (**B**) Scatterplot of DEGs comparing Cdk4^WT^ and Cdk4^R/R^ mice as well as E2F3^WT^ and E2F3^mKO^ mice. Blue dots represent type I fiber markers that are upregulated in Cdk4^R/R^ muscle and downregulated in *E2F3*^mKO^ muscle. (**C**) Log_2_ fold change values determined by RNA-Seq from groups of upregulated genes in Cdk4^R/R^ muscle and downregulated genes in *E2F3*^mKO^ muscle representing myofiber, ion transport, and muscle potential genes, including type I fiber markers. (**D**) Venn diagram showing the overlap of DEGs (*P* < 0.05) in Cdk4^WT^ and Cdk4^mKO^ (KO) muscle as well as E2F3^WT^ and E2F3^mKO^ muscle (top), and GO analysis of 483 overlapping genes (bottom). (**E**) Heatmap of the overlapped 483 genes defined in **D** and gene ontology (GO) analysis of co-upregulated (red) or codownregulated (green) genes with color intensities indicating *Z* score (*n* = 3 mice each group).

A comparison of RNA-Seq data revealed a set of 199 genes that overlapped in muscle from *Cdk4*^R/R^ and *E2F3*^mKO^ mice, with genes representing transition between fast and slow fibers the most significant (Figure 6A). Cluster analyses revealed that slow type 1 muscle fiber genes were increased in *Cdk4*^R/R^ muscle, whereas those same genes were suppressed in *E2F3*^mKO^ muscle (Figure 6, B and C). We also found an overlap of 483 genes, comprising primarily of markers of mitochondrial bioenergetics and lipid handling and storage, between *E2F3*^mKO^ and *Cdk4*^KO^ muscles compared with WT muscles (Figure 6, D and E). Of the 483 genes, expression of 58 genes representing lipid-related pathways correlated with the status of Cdk4 or E2F3 (Supplemental Figure 15). Expression of these lipid-pathway genes was either coordinately increased or decreased (30 of 58 genes) or was regulated in the opposite direction (28 of 58 genes) in *Cdk4*^KO^ and *E2F3*^mKO^ muscle. Furthermore, we observed that 196 mitochondrial-specific genes were codownregulated in *Cdk4*^KO^ and *E2F3*^mKO^ muscle, suggesting common Cdk4-E2F3–dependent regulation (Figure 6E).

To obtain further molecular insight into how p16^Ink4a^-Cdk4-E2F3 regulates oxidative fiber development and mitochondrial function, we studied primary myoblasts from *E2F3*^mKO^, *Cdk4*^R/R^, and *Cdk4*^KO^ mice as well as the effects of knocking down p16^Ink4a^ in WT myoblasts (Supplemental Figure 16, A and B). Proliferation was slightly increased in *Cdk4*^R/R^ myoblasts but reduced in *Cdk4*^KO^ myoblasts. Knockdown of p16^Ink4a^ during differentiation resulted in upregulation of markers representing both oxidative and glycolytic muscle fibers in addition to increases in the expression of muscle mitochondrial markers (Supplemental Figure 16C). Differentiation of *Cdk4*^R/R^ myoblasts led to upregulation of markers representing type 1 oxidative muscle fibers and mitochondria, without a change in expression levels of markers representing glycolytic fibers (Supplemental Figure 16D). In contrast, knockdown of E2F3 during myoblast differentiation suppressed most muscle mitochondrial markers, while having no observable effect on the expression of muscle fiber–type markers (Supplemental Figure 16E). Collectively, based on the analyses of data obtained using cell culture and in vivo mouse modeling under different conditions, we infer that the p16^Ink4a^-Cdk4-E2F3 signals (a) differentially affect myoblast proliferation and its regeneration potential (based on the CTX injury model) and, (b) upon differentiation, these signals selectively regulate the expression of specific muscle fiber–type markers and muscle mitochondrial genes.

### E2F3–PGC-1α levels in mouse models of diabetes and obesity.

**Figure 7:**
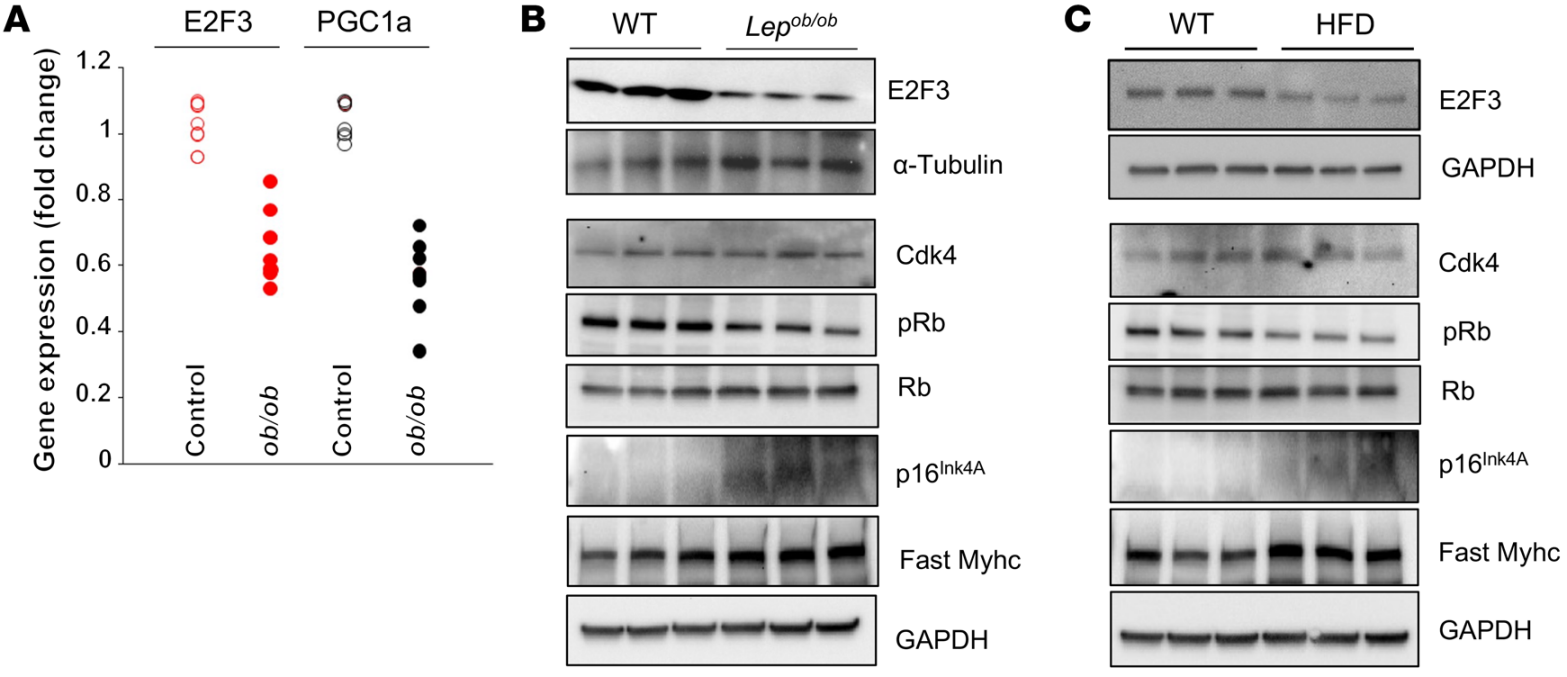
Correlation of E2F3–PGC-1α levels in mouse models of diabetes and obesity. (**A**) *E2F3* and *Pgc-1*α mRNA expression in QA muscle from *Lep*^ob/ob^ mice and age- and sex-matched control mice. (**B** and **C**) Protein expression in skeletal muscle of (**B**) *Lep*^ob/ob^ mice and (**C**) HFD-fed mice compared with RD-fed age and sex-matched control mice (*n* = 3–5 mice each group). *P < 0.05, **P < 0.01, ***P < 0.001 by 2-tailed Student’s *t* test.

Next, we investigated the association of E2F3 and PGC-1α using the leptin-deficient obese (*Lep*^ob/ob^) mouse and the HFD-induced obese/diabetic mouse models. Significantly reduced expression of muscle-specific, energy sensing, fatty acid oxidation, and mitochondrial genes along with reduced levels of *Cdk4*-*E2F3*-*PGC-1*α transcripts were seen in skeletal muscles of *Lep*^ob/ob^ mice (Supplemental Figure 17). Muscle from *Lep*^ob/ob^ mice showed reduced *E2F3* and *Pgc-1*α gene expression (Figure 7A). Protein levels of p16^Ink4a^ were elevated and those of phospho-RB and E2F3 were reduced in muscle tissue from *Lep*^ob/ob^ and HFD-fed mice (Figure 7, B and C). Importantly, the levels of Cdk4 protein were unchanged in either *Lep*^ob/ob^ or HFD mice. which is consistent with the notion that the overexpressed p16^Ink4a^ inhibitor binds to Cdk4 and suppresses its activity without alteration in Cdk4 protein level. Thus, progression of obesity and diabetes in these two well-characterized mouse models involves dampening of Cdk4-E2F3 activity in skeletal muscle via upregulation of the p16^Ink4a^ inhibitor.

### Correlation of CDK4, CDKN2A, and E2F3 expression with markers of fiber type and mitochondria in human skeletal muscle.

**Figure 8:**
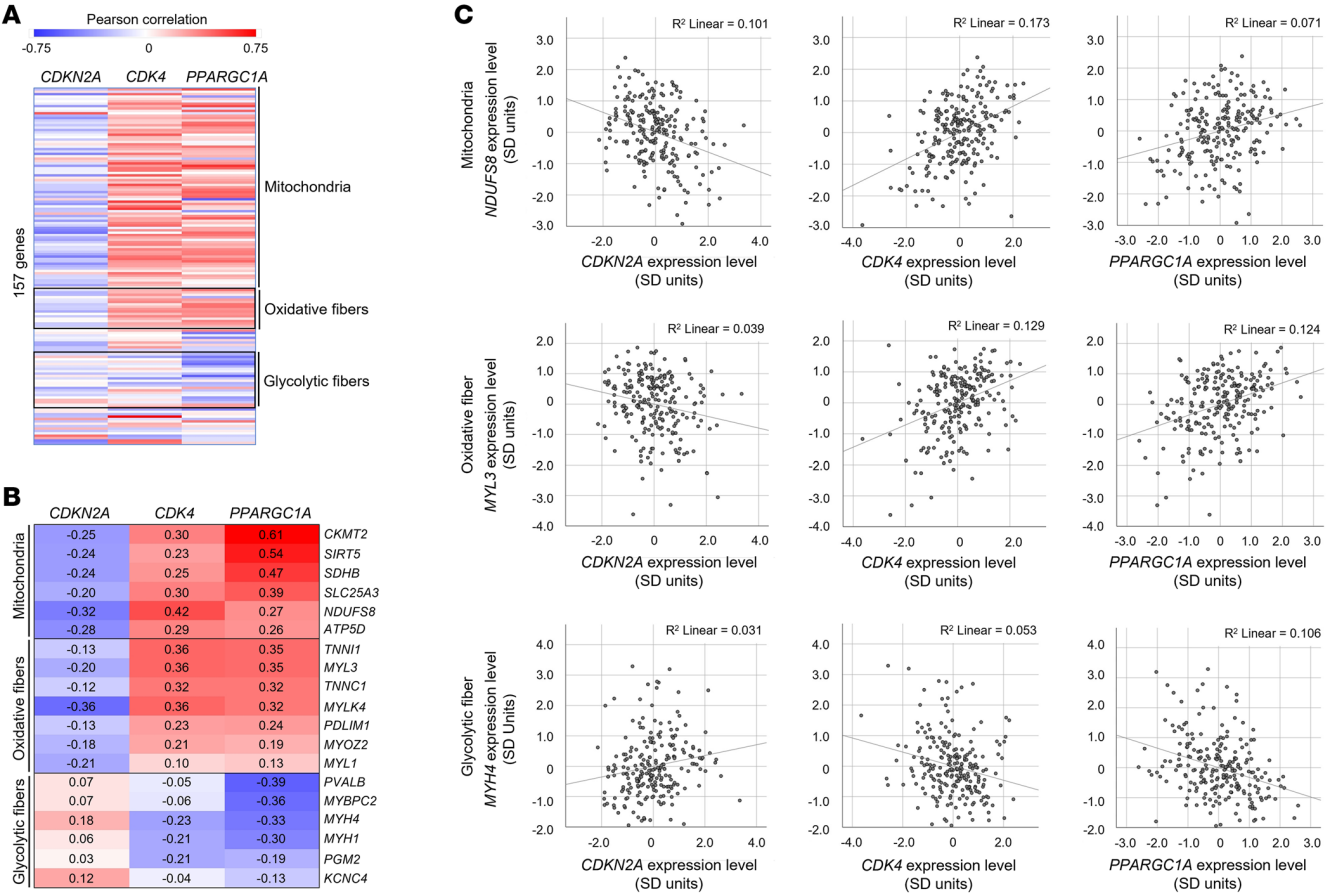
Opposite correlation of *CDK4* and *CDKN2A* expression with markers of fiber type and mitochondria in nondiabetic human skeletal muscle. (**A**) Heatmap showing Pearson’s partial correlations (adjusted for age and genetic admixture) of mRNA levels (standardized across batches and sexes) from markers representing mitochondria, oxidative fibers, and glycolytic fibers, with levels of expression of *CDKN2A*, *CDK4*, and *PPARGC1A* in skeletal muscle biopsies from healthy individuals (*n* = 221). (**B**) Heatmap and (**C**) scatterplots depicting correlation of mRNA levels of select markers representing mitochondria, oxidative fibers, and glycolytic fibers in skeletal muscle biopsies from individuals (*n* = 221) with levels of expression of *CDKN2A*, *CDK4*, and *PPARGC1A*. Each value in the heatmap represents the Pearson’s partial correlation coefficient adjusted for age and genetic admixture. Red indicates high positive correlation, and blue indicates high negative correlation. Smaller circles in scatterplots denote gene expression levels of all individuals and each regression line is shown along with the goodness of fit (*r*^2^) by linear regression analysis.

Using 221 nondiabetic human muscle biopsies, expression levels of *CDK4* and *CDKN2A* were correlated with levels of markers representing oxidative and glycolytic muscle fibers and muscle mitochondria (Figure 8 and Supplemental Figure 18). Expression levels of *CDK4* and *PPARGC1A* overall correlated positively with expression of markers of oxidative muscle fibers and muscle mitochondria and negatively with expression of glycolytic muscle fiber markers. In contrast to that observed with *CDK4* and *PPARGC1A*, expression levels of *CDKN2A* overall correlated positively with expression of glycolytic muscle fiber markers and negatively with expression of markers of oxidative muscle fibers and mitochondria.

**Figure 9:**
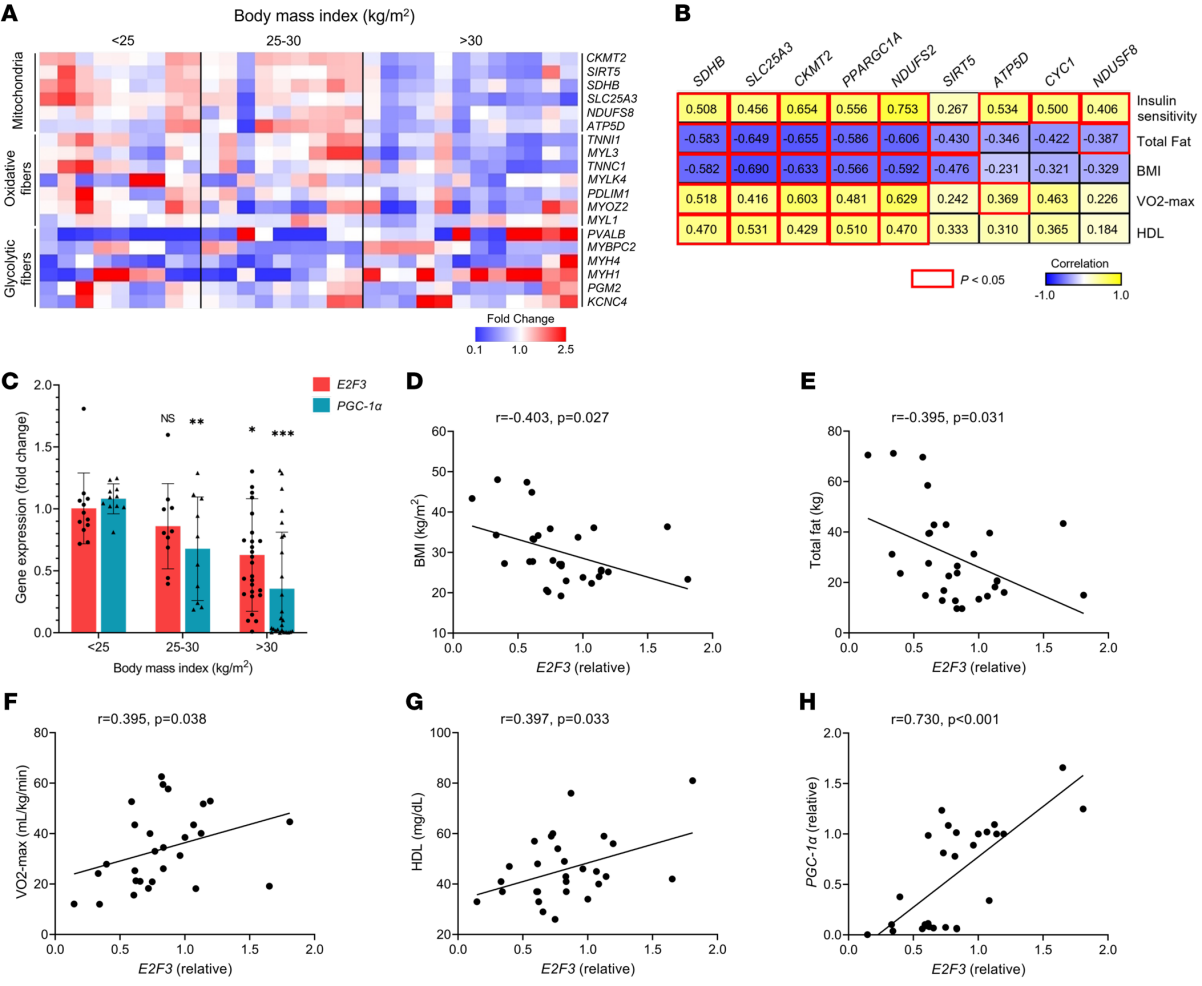
Association of metabolic measures with markers of muscle fiber type and mitochondria in human vastus lateralis muscle biopsies. (**A**) Heatmap correlating mRNA levels of markers representing mitochondria, oxidative fibers, and glycolytic fibers in skeletal muscle biopsies from individuals (*n* = 30) with BMIs of less than 25, between 25 and 30, and more than 30 kg/m^2^. The fold change scale is provided. (**B**) Correlation of indicated metabolic parameters with mitochondrial markers. Positive and negative correlation scores are shown inside boxes; values in boxes with red outline are significant (*P* < 0.05). (**C**) Transcript levels of *E2F3* and *PGC-1*α correlated with increasing BMI in individuals (*n* = 49). (**D**–**H**) Scatterplots of *E2F3* expression correlated with BMI, total fat, VO2 max, HDL, and *PGC-1*α. The strength of association was quantified by the Pearson’s correlation index. Data are shown as the mean ± SEM. **P < 0.01, ***P < 0.001 by 2-tailed Student’s *t* test.

To investigate if muscle fiber–type and mitochondrial markers were associated with metabolic parameters, we performed gene expression analyses using a subset of 30 vastus lateralis muscle biopsies obtained from a separate 49 individual cohort (Supplemental Table 1). Markers of slow oxidative muscle fibers and mitochondria were elevated in biopsies from lean individuals, while those same markers were reduced in biopsies from obese individuals (Figure 9A). In contrast, markers of glycolytic muscle fibers were elevated in biopsies from obese individuals, and, in general, those markers were suppressed in biopsies from leaner individuals. Moreover, expression levels of mitochondria-specific genes correlated positively with insulin sensitivity from frequently sampled intravenous glucose tolerance test, exercise capacity based on VO2 max test, and HDL cholesterol levels (Figure 9B). In contrast, the mitochondrial markers correlated negatively with total fat and BMI.

Using the vastus lateralis muscle biopsies from the 49 individual cohort, we next examined the correlation of *E2F3* and *PGC-1*α mRNA expression levels with markers of adiposity, fitness level, and diabetes. There was no relationship between *E2F3* or *PGC-1*α levels and age or sex. We found a significant reduction in *E2F3* and *PGC-1*α levels as a function of increasing BMI, with progressively lower levels in muscle derived from overweight (BMI, 25–30 kg/m^2^) and obese (BMI, over 30 kg/m^2^) individuals compared with individuals with healthy BMI (less than 25 kg/m^2^) (Figure 9C). Expression of E2F3 was associated negatively with BMI and total fat (Figure 9, D and E) and positively with VO2 max, HDL levels, and *PGC-1*α expression (Figure 9, F–H). Multiple linear regression analyses showed that the correlation between VO2 max and skeletal muscle E2F3 expression trended toward an independent association (*P* = 0.062) but that the correlation was significantly driven by BMI (*P* < 0.001) and sex (*P* = 0.008), with no effect of age (*P* = 0.479). We observed statistically significant negative associations of *E2F3* and *PGC-1*α levels with BMI, percentage body fat, total body fat mass, total lipid content of the vastus lateralis, and intramyocellular lipid content of the vastus lateralis muscle prior to biopsy (Supplemental Table 2). We further observed significant positive associations of *E2F3* and *PGC-1*α levels with insulin sensitivity index from frequently sampled intravenous glucose tolerance test (FSIVGTT), suggesting a correlation of both proteins with improved insulin sensitivity (Supplemental Table 2).

## Discussion

In an effort to better understand the genetic landscape, bioinformatics approaches identified candidate loci associated with T2D (22, 23, 53–55). The *CDKN2A* gene, which codes for the Cdk4 inhibitor p16^Ink4a^, was prominently associated with T2D, raising the possibility that Cdk4 — the primary substrate for p16^Ink4a^ — may be involved in glucose disposal and insulin sensitivity. Here, we report that Cdk4 regulates skeletal muscle fiber–type proportions and muscle mitochondrial function. Specifically, we showed that Cdk4-E2F3-PGC-1α signals preferentially increase oxidative myofibers in skeletal muscle and stimulate mitochondrial biogenesis and bioenergetics, enabling greater exercise capacity, improved glucose tolerance, and protection from diabetes and obesity. It has long been considered that skeletal muscle fiber composition is genetically determined (56). Moreover, skeletal muscle fiber type substantially influences muscle’s exercise capacity and glucose clearance ability (10). In agreement with this, leanness and insulin sensitivity are associated with increased oxidative capacity in skeletal muscle (57). Furthermore, slow type 1 oxidative fiber numbers are inversely correlated with obesity (58).

The PGC-1α coactivator regulates transcriptional programs that coordinate specific adaptive responses in skeletal muscle, fasting responses in the liver, and nonshivering thermogenesis in brown adipose tissue (59, 60). Thus, PGC-1α targets genes involved in mitochondrial biogenesis, oxidative phosphorylation, the TCA cycle, fatty acid oxidation, and gluconeogenesis (61). Specifically, in skeletal muscle, PGC-1α is a key modifier of muscle’s endurance capacity and is implicated in regulation of baseline muscle function, mitochondrial energetics, development of specific fiber types, fiber-type switching during exercise, and whole-body glucose homeostasis (62). Via promoting mitochondrial function, PGC-1α regulates muscle’s exercise capacity and its glucose clearing ability (60, 63). PGC-1α is expressed in oxidative myofibers (38) and is rapidly induced after endurance exercise (64). Its overexpression promotes oxidative fiber–type formation and increased endurance exercise capacity (38, 65). In contrast, muscle-specific ablation of PGC-1α results in increased glycolytic muscle fibers, fiber damage, and a lower capacity for endurance exercise (66). Muscle-specific loss of PGC-1α also perturbs glucose homeostasis, due, at least in part, to an associated β cell dysfunction (67). Although PGC-1α is a key modifier of muscle glucose metabolism and muscle’s endurance capacity, there is ongoing debate regarding its precise role in (a) baseline muscle function, (b) mitochondrial energetics, (c) development of specific fiber types, (d) fiber-type switching during exercise, and (e) whole-body glucose homeostasis (38, 66, 68–71).

In contrast to our results, muscle-specific overexpression of PGC-1α using the MCK promoter causes insulin resistance in mice fed a HFD (72). It is likely that the levels of PGC-1α in muscle are considerably higher in MCK-PGC-1α–transgenic mice compared with those in *Cdk4*^R/R^ mice. Moreover, MCK-PGC-1α mice have increased mitochondria in the fast type II skeletal muscle fibers but not slow type I fibers (38). One explanation for the difference is that *Cdk4*^R/R^ mice have increased energy expenditure (Figure 1M), unlike MCK-PGC-1α mice (72). Increased energy expenditure has been hypothesized to prevent overloading of the β oxidation machinery of mitochondria in the presence of excess fatty acids, thereby preventing insulin resistance (71). Regardless, these differences between MCK-PGC-1α mice and *Cdk4*^R/R^ mice fed a HFD show that there must be mechanisms in addition to increased PGC-1α expression or the greater numbers of muscle mitochondria in *Cdk4*^R/R^ mice that account for the improved metabolic phenotype.

Using *Cdk4*^KO^ mice and derivative cells, along with pharmacological inhibition, we showed that Cdk4 loss is detrimental to muscle mitochondrial function. Of note, the effects of a Cdk4 inhibitor on oxygen consumption in C2C12 cells (Supplemental Figure 10F) suggest more rapid effects on energy expenditure than expected if Cdk4 solely regulated fiber-type differentiation. Our observations demonstrating substantial and overlapping changes in muscle mitochondrial markers from *Cdk4*^KO^ and *E2F3*^mKO^ mice are consistent with this. The findings that p16^INK4a^-Cdk4-E2F3 signals modify mitochondrial numbers and activity are especially relevant considering the decline in muscle mitochondrial function during aging when metabolic diseases like T2D and mobility impairments are typically observed (73). Indeed, an age-associated decline in mitochondrial function contributes to insulin resistance in elderly individuals (74). Moreover, skeletal muscle insulin resistance is associated with defects in mitochondrial oxidative phosphorylation (75). How p16^Ink4a^-Cdk4-E2F3 signals affect mitochondrial numbers and function is not entirely clear, and whether targets in addition to PGC-1α are involved remains to be determined.

We observed nonoverlapping gene expression patterns of molecules representing lipid storage and metabolism in the *Cdk*4^KO^ compared with *E2F3*^mKO^ muscle (Figure 6E). Further analyses showed evidence of lipid mobilization effects that were dependent on the status of Cdk4-E2F3 signaling. Thus, we observed a set of genes that appeared to be coordinately regulated by Cdk4-E2F3, whereas there was another set of genes that was unique to the status of Cdk4 or E2F3 (Supplemental Figure 15). These observations imply that while the muscle mitochondrial gene expression programs might be conserved, Cdk4 and E2F3 might serve unique roles in mobilization and storage of lipid within muscle tissue. Cdk4 and E2F3 have roles within the traditional adipose tissue, and whether similar mechanisms are involved in skeletal muscle is unclear (76–78).

Much of the data we report using the various in vivo and cell culture models are consistent with a direct effect of the CDK4-E2F3 pathway on the oxidative phenotype of skeletal muscle. However, we did not find evidence of increased lipid oxidation in *Cdk4*^R/R^ mice. Increased muscle mass, increased muscle fatty acid oxidation, enhanced daytime RER, and elevated ambulatory activity might contribute to the overall higher metabolic rate in *Cdk4*^R/R^ mice, leading to less fuel available for storage in adipose and other tissues and, consequently, resulting in improved glucose metabolism. Finally, primary myoblasts from *Cdk4*^R/R^ mice had increased expression of *Pgc-1*α and *Uqcrc2* while pharmacological inhibition of Cdk4 in C2C12 myotubes reduced the expression *Pgc-1*α, *Tfam,* and slow oxidative fiber genes. These in vitro results suggest that the oxidative phenotype of *Cdk4*^R/R^ muscle may be due to direct effects of the Cdk4 pathway and not only due to increased ambulating activity.

To our knowledge, this is the first report linking E2F3 to changes in glucose homeostasis, mitochondrial energetics, and muscle function. Interestingly, the related E2F1 transcription factor represses genes that regulate mitochondrial function in BAT and muscle (79). It is noteworthy that the two E2F3 isoforms — E2f3a and E2f3b — target genes that are involved in lipid metabolism and myogenic differentiation in an isoform-specific manner (78). E2F3 and PGC-1α levels are coordinately reduced in states of obesity — HFD-fed mice, *Lep*^ob/ob^ mice, and overweight and obese humans — consistent with a conserved underlying mechanism. Along the same lines, the observations that *Cdk4*^R/R^ mice are stronger, exhibit greater muscle exercise capacity, and enhanced VO2 max, whereas *E2F3*^mKO^ mice display reduced exercise capacity, support the notion that Cdk4-E2F3 signals regulate muscle exercise function.

Interestingly, Puigserver and colleagues showed that insulin activates Cdk4, which, in turn, suppresses hepatic glucose production, independently of cell cycle progression, by modulating levels of PGC-1α acetylation (80). With our prior work demonstrating the importance of Cdk4 to the pancreatic β cell (29–32), these findings support the concept that Cdk4 is a key regulator of glucose homeostasis via action on multiple metabolic organs. While it is plausible that the skeletal muscle–specific effects reported here are influenced by Cdk4’s role in other cell types involved in glucose homeostasis, the data using primary myoblasts and C2C12 cells supports a cell autonomous role for Cdk4 in muscle fiber–type determination and muscle mitochondrial function. Taken together, these observations suggest that disruption of Cdk4 activity, regulation of its downstream effectors like E2F3, or aberrant activation of its inhibitor p16^Ink4a^ could simultaneously disable glucose-stimulated insulin secretion and insulin action — two prominent facilitators of diabetes progression. Consistent with this concept, elevated p16^Ink4a^ levels, despite the equivalent Cdk4 expression in the WT and mutant genotypes, are suggestive of reduced Cdk4 activity in muscle tissue from *Lep*^ob/ob^ and HFD obese/diabetic mice. In agreement with this, we detected reduced levels of phospho-RB and E2F3 proteins in muscle tissue from *Lep*^ob/ob^ and HFD-fed mice. Likewise, we found E2F3 transcript levels negatively associating with unfavorable metabolic parameters in muscle biopsies of patients with T2D or high BMI.

Our results using human skeletal muscle biopsies supports a conserved role for the CDK4 pathway in skeletal muscle fiber–type determination across species. Observations that substantial correlations exists between the levels of *CDK4*, *CDKN2A*, and *PPARGC1A* and expression levels of markers representing oxidative and glycolytic muscle fiber and muscle mitochondria in nondiabetic individuals suggest the potential relevance of these molecules in regulation of muscle fiber type and muscle mitochondria. Finally, it is noteworthy that we observed minimal variation in the marker gene correlation, given that these data were derived from muscle biopsies from nondiabetic human donors and a large variability is typically anticipated. Subsequent studies can inquire whether these molecules play a causal role in the pathogenesis of metabolic diseases. Given that PGC-1α is highly conserved between mice and humans, with 94% identity, we believe that knowledge about mechanisms that regulate its expression and, in turn, modify its function, is relevant to understanding normal and pathologic muscle physiology and metabolism.

The association of lower BMI with markers of oxidative fibers and mitochondria and of higher BMI with markers of glycolytic fibers support the notion that muscle fiber type influences BMI. In addition, the positive association of mitochondrial markers with insulin sensitivity, VO2 max, and HDL levels and negative association with total fat and BMI further supports the existence of a conserved mechanism regulating muscle metabolic function. Finally, the observations that *E2F3* and *PPARGC1A* levels associate with lower BMI, low total fat, low percentage body fat, decreased lipid content in muscle, and with higher VO2 max, higher HDL levels, and insulin sensitivity are consistent with a conserved molecular mechanism.

The findings suggest that mutations at the *CDKN2A* locus may limit skeletal muscle PGC-1α induction and contribute to insulin resistance, diminished physical fitness, and increased adiposity. Because the human studies reported here were all conducted in adults using noninterventional retrospective study designs, confounding factors related to the pleiotropic effects of CDK4 signaling limit the interpretation of our findings. For instance, patients with low CDK4 signaling in skeletal muscle may have reduced physical activity levels, reinforcing the associations we found in our studies. The consistency of the relationships we observe supports the role of skeletal muscle CDK4 signaling in chronic metabolic disease. Particularly, findings related to VO2 max generally track with those found for HDL cholesterol, as do findings for total body fat percentage and skeletal muscle lipid content. In summary, these findings strongly support a role for the CDKN2A-CDK4-E2F3 axis in regulation of skeletal muscle fiber–type proportion and function, and, thus, they are of clinical significance to muscle disorders and chronic diseases, like insulin resistance and diabetes, that involve muscle dysfunction.

## Methods

### Mice.

The generation of *Cdk4*^KO^ and *Cdk4*^R/R^ mice has been described previously (31). Mice were fed ad libitum with either a standard mouse chow (NIH07 diet) or with HFD (55% [w/w] fat content; 5.45 kcal/g; TD 97075, Harlan Teklad, or 60% kcal fat, 5.24 metabolizable kcal/g; D12492, Research Diets). To generate muscle-specific E2F3-knockout (*E2F3*^mKO^) mice, *E2F3*^fl/fl^ mice (50, 51) were crossed with MLC-Cre mice (52). In vivo experiments used male mice (*n* = 5–8 of each genotype) at 6–8 weeks of age.

### Glucose and insulin tolerance tests, hyperinsulinemic-euglycemic clamp assay, indirect calorimetry and body composition analyses, immunohistochemistry, electron microscopy and Western blotting, real-time RT-PCR, and mitochondrial DNA.

These analyses were done as described previously (81) using male mice (*n* = 5–8 of each genotype at 6–8 weeks of age). Supplemental Tables 3 and 4 provide antibody and primer information.

### Grip strength, endurance, and exercise capacity.

Grip strength was measured using the inverted screen test as described previously (82). Exercise capacity was tested using a Columbus Instruments rodent treadmill (Model Eco-6M) set at a 10-degree incline. Total time, distance, maximum speed, and work were recorded at the time of exhaustion. The testing protocol was as follows: 10 minutes with belt speed at 10 m/min, followed by 12 m/min for 5 minutes, and 15 m/min for 3 minutes; then, the belt speed was incrementally increased by 1.8 m/min every 3 minutes until the mouse became exhausted. Endurance capacity test was also conducted on the Columbus Instruments treadmill set at a 10-degree incline. Ramp speed was increased more slowly over time as follows: 8/min for 30 minutes, 9 m/min for 15 minutes, and then 1 m/min increase every 10 minutes until exhaustion. Male mice (*n* = 9–11 of each genotype) were used at 8–10 weeks of age.

### Muscle fiber analyses.

Quadriceps, TA, and EDL muscle types were used. 10 μm–thick sections were stained with antibodies according to standard protocols. Details of the antibodies and primers used in the characterization studies are provide in Supplemental Tables 3 and 4, respectively. Measurement of cross-section areas was performed using ImageJ software (NIH). Histochemical SDH staining and enzymatic activity detection were assayed as described elsewhere (83).

### Muscle regeneration analyses.

Muscle injury was induced using a protocol described elsewhere (42). Eight- to 10-week-old male mice (*n* = 6 of each genotype) were anesthetized with 1%–3% isoflurane/O_2_ and injected with 50 μL of 20 μM CTX (Sigma-Aldrich) solution into TA muscle to induce muscle injury. Simultaneously, mice were provided water supplemented with IdU/CldU (0.1% solution) ad libitum and euthanized 7 or 14 days after CTX injection for analysis.

### Cell culture and myogenic differentiation.

MEFs and C2C12 cells were grown in DMEM media supplemented with 10% FBS and 1% antibiotics. Primary myoblasts isolated from mouse hind limb muscles were grown in DMEM/F10 media supplemented with 20% FBS, 1% antibiotics, and 10 ng/mL bFGF. Cdk4, E2F3, or p16 knockdown in C2C12 cells or primary myoblasts was accomplished using shRNA in pLKO.1-puro lentiviral vectors (Sigma-Aldrich). pCMV-E2F3 plasmid or adenovirus Ad-human CDK4 (Vector Biolabs) was used for ectopic overexpression in C2C12 cells. Myogenic differentiation of C2C12 or primary myoblasts was initiated by switching the cells to medium containing 2% or 5% horse serum and continued for 4–6 days, until myotubes were visible. Cdk4 inhibitor (100 nM, IDCX), was included in myocyte differentiation media during the entire differentiation period.

### Oxygen consumption.

C2C12 cells were grown in normal DMEM medium containing 10% FBS and 1% antibiotics to achieve 70% confluency. Cells were trypsinized and incubated with Cdk4 inhibitor (100 nM, IDCX) and monitored for oxygen consumption using BD oxygen plate.

### In vivo fatty-acid oxidation.

In vivo fatty-acid ([1-^14^C] oleic acid) oxidation was measured using a method similar to that described previously (84). Mice were fasted overnight, injected with [1-^14^C] oleic acid (Perkin Elmer NEC317, 1 μCi in 200 μL saline i.p.), and placed into a sealed chamber connected to an air pump (Thomas Scientific # 7893B05) and a 50 mL tube containing 3 M NaOH solution to trap expired ^14^CO_2_. One milliliter of aliquots was taken 30 and 240 minutes after injection of [1-^14^C] oleic acid to calculate the oxidation of [1-^14^C] oleic acid to ^14^CO_2_.

### Luciferase reporter assay.

2 × 10^5^ cells/well were transfected with 1 μg/well of PGC-1α reporter and E2F plasmids together with 0.1 μg/well of Renilla-luc plasmid using Fugene6 transfection reagent (Promega). Luciferase activity was measured using the Promega dual luciferase assay kit.

### ChIP assay.

ChIP assay was performed using the chip-IT kit (Active Motif), following the manufacturer’s instructions and as described elsewhere (81). E2F3 binding on the PGC-1α promoter was calculated by subtracting the intensity value of the band detected upon immunoprecipitation with the anti-E2F3 antibody from the intensity value detected from the input band, and the difference is presented as percentage arbitrary units.

### RNA-Seq library preparation, sequencing, and bioinformatics analysis.

Quadriceps and soleus muscles were used (*n* = 3 each group). mRNAs were purified from total RNA using the NEBNext Poly(A) mRNA Magnetic Isolation Module (NEB), and RNA integrity and quantitation were assessed using the RNA Nano 6000 Assay Kit of the Bioanalyzer 2100 system (Agilent Technologies). Sequencing libraries were generated using the NEBNext Ultra RNA Library Prep Kit for Illumina (NEB) and sequenced on the Illumina NovaSeq6000 with 150 bp paired-end reads. For bioinformatics analysis, paired-end clean reads were aligned to *Mus musculus* mm10 reference genome using the Spliced Transcripts Alignment to a Reference (STAR) software. FeatureCount was used to count the read numbers mapped of each gene, and then reads per kilobase of exon model per million mapped reads (RPKM) of each gene was calculated for each gene as a measure of expression level. Differential expression analysis was performed using DESeq2 R package. Genes with an adjusted *P* value of less than 0.05 found by were DESeq2 assigned as differentially expressed. Gene ontology analysis and Kyoto Encyclopedia of Genes and Genomes pathway analysis were conducted with the clusterProfile R package to identify differentially expressed genes at the biologically functional level.

### Human study.

Individuals with varying body composition consented to an National Institute of Diabetes and Digestive and Kidney Diseases (NIDDK) institutional review board–approved study for extensive metabolic phenotyping (Clinicaltrials.gov NCT00428987) that included a dual energy x-ray absorptiometry scan, a FSIVGTT, a maximal exercise stress test, magnetic resonance spectroscopy (MRS) of the vastus lateralis muscle, and a biopsy of the vastus lateralis muscle (85, 86). All individuals were older than 18 years (41.1 ± 12.1 years), and 51% of individuals were female. MRS was performed 3 hours after all individuals consumed a meal standardized for energy (30% of daily energy needs) and macronutrient composition (55% carbohydrate, 30% fat, 15% protein). Muscle biopsies were obtained after MRS under local anesthesia (1% lidocaine) using the Bergstrom technique and stored at –80°C until RNA was extracted.

Volunteers of American Indian ethnicity (men, 68%; age, 29.4 ± 7.4 yr; BMI, 33.3 ± 7.1 kg/m^2^; mean ± SD) were admitted to the Clinical Research Unit, NIDDK, to participate in a longitudinal study to determine risk factors of diabetes and obesity (Clinicaltrials.gov NCT00340132). Before participation, volunteers were fully informed of the nature and purpose of the study, and written informed consent was obtained. Volunteers were between 18 and 55 years and were determined to be healthy by physical examination, medical history, and laboratory tests. Upon admission, volunteers were placed on a daily weight-maintaining balanced diet (50% carbohydrate, 30% fat, and 20% protein) for at least 3 days prior to any metabolic assessment. Glucose tolerance was assessed by a 3-hour 75 g OGTT, and all volunteers were free from diabetes according to the ADA diagnostic criteria. After at least 3 days on the weight-maintaining diet and following an overnight fast, individuals underwent a needle biopsy of the vastus lateralis muscle under local anesthesia with 1% lidocaine. Total RNA was extracted, and the cDNA was analyzed using the Human Exon 1.0 ST Array microarray chips (Affymetrix) as previously described in detail (87). The primers used are listed in Supplemental Table 5.

### Statistics.

Statistical significance between groups was determined using 2-tailed Student’s *t* test or 1-way ANOVA. In addition, for human studies, gene expression data were standardized across batches and sexes, and intergene correlations were quantified using Pearson’s correlation index after adjustment for age and genetic admixture. Data from mouse studies are expressed as mean ± SEM, and data from human studies are expressed as mean ± SD. Values plotted are mean and SEM. *P* values of less than 0.05 were considered significant.

### Study approval.

All animal studies protocols were approved by the NIDDK/NIH Animal Care and Use Committee. All human clinical protocols were approved by the NIDDK/NIH Institutional Review Board.

### Data availability.

Data were deposited in the NCBI’s Gene Expression Omnibus database (GEO GSE101820). RNA-Seq data were deposited in GEO (GSE185154).

## Author contributions

YJB and HY conducted most of the experiments, analyzed results, and prepared figures. OG and DAS performed muscle metabolism and exercise-related experiments in mice. IP helped with confocal imaging for the CTX experiment. PMZ performed electron microscopy. BSA and MCS provided human-related data. ACM provided expertise on muscle phenotyping studies and helped with drafting the manuscript. PP provided human muscle biopsies data, correlations, and figures. SGR conceived the idea for the project and wrote the paper with input from YJB, HY, BSA, PP, OG, and ACM.

## Supplementary Material

Supplemental data

Supporting data values

## Version 1 07/03/2023

Electronic publication
