# Supplementary material for: CDK4-E2F3 signals enhance oxidative skeletal muscle fiber numbers and function to affect myogenesis and metabolism
Source: J Clin Invest. 2023 Jul 3;133(13):e162479. doi: 10.1172/JCI162479 (PMC10313363; doi:10.1172/JCI162479)
Supplement: Supplemental data [file jci-133-162479-s177.pdf]

Figure S1

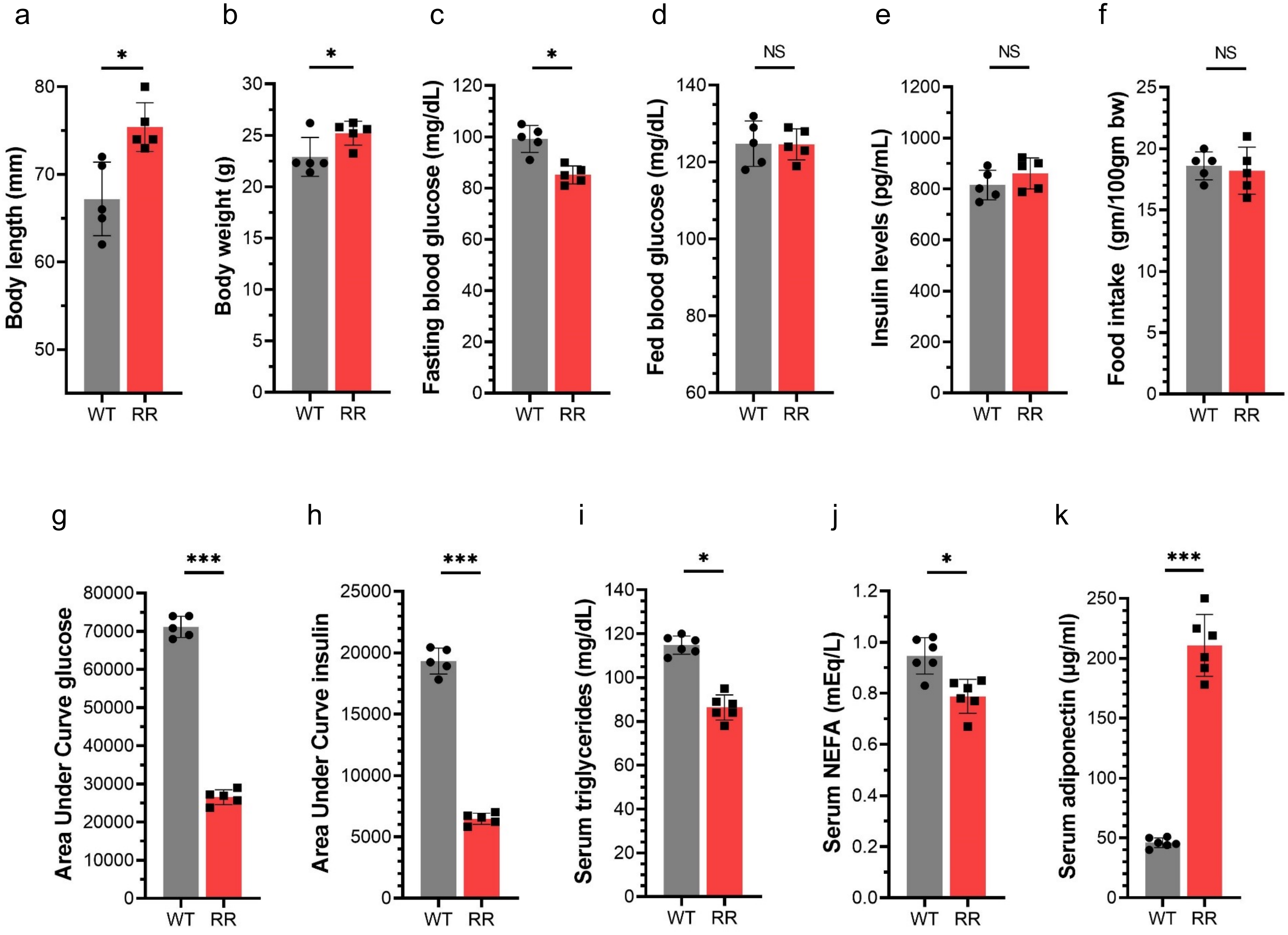
a
b
c
d
e
f
g
h
i
j
k
Body length (mm)
Body weight (g)
Fasting blood glucose (mg/dL)
Fed blood glucose (mg/dL)
Insulin levels (pg/mL)
Food intake (gm/100gm bw)
Area Under Curve glucose
Area Under Curve insulin
Serum triglycerides (mg/dL)
Serum NEFA (mEq/L)
Serum adiponectin (µg/ml)
WT
RR
*
***
NS

Figure S2

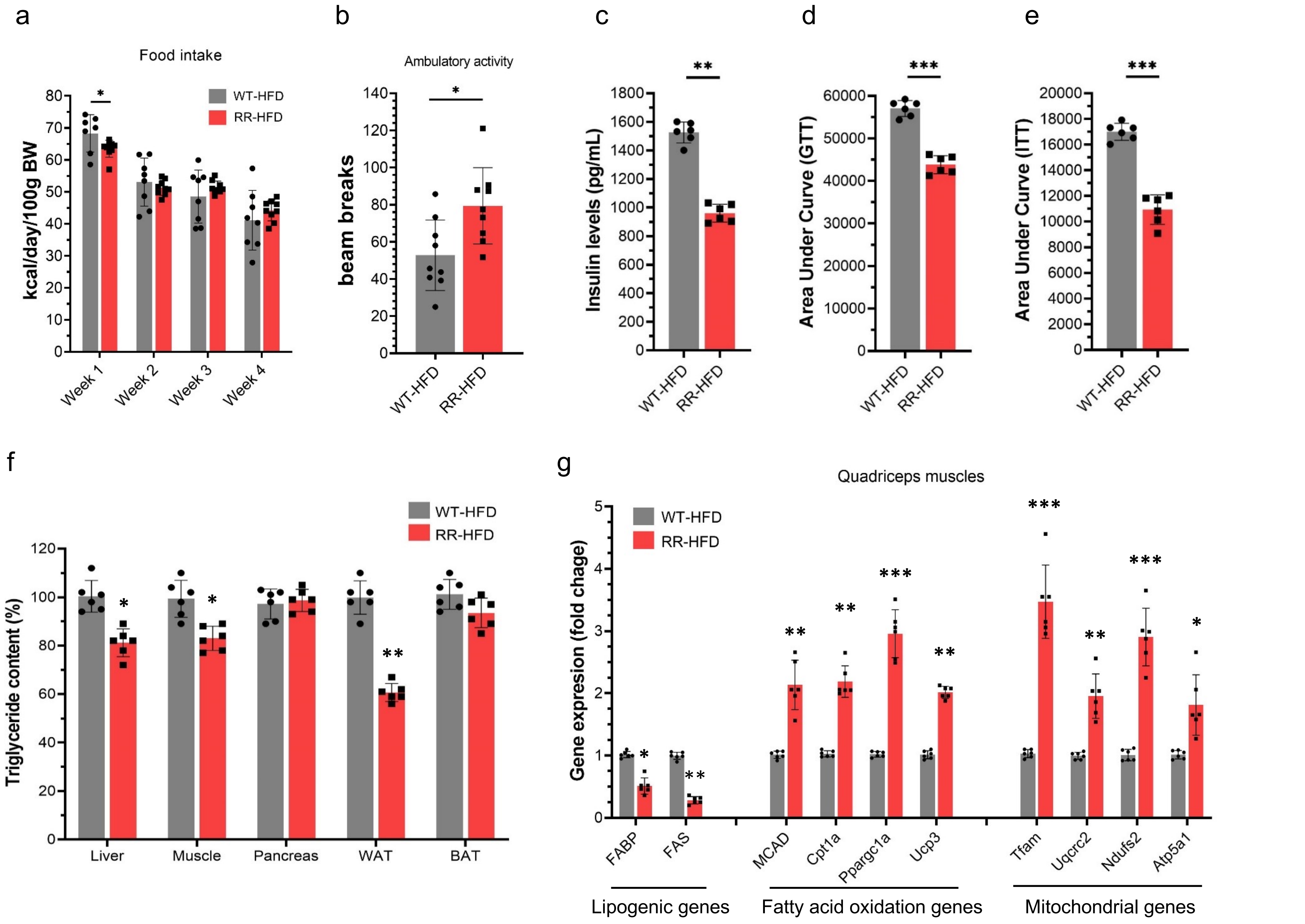
a
Food intake
kcal/day/100g BW
WT-HFD
RR-HFD
Week 1
Week 2
Week 3
Week 4
b
Ambulatory activity
beam breaks
WT-HFD
RR-HFD
c
Insulin levels (pg/mL)
WT-HFD
RR-HFD
d
Area Under Curve (GTT)
WT-HFD
RR-HFD
e
Area Under Curve (ITT)
WT-HFD
RR-HFD
f
Triglyceride content (%)
WT-HFD
RR-HFD
Liver
Muscle
Pancreas
WAT
BAT
g
Quadriceps muscles
Gene expression (fold chage)
WT-HFD
RR-HFD
FABP
FAS
MCAD
Cpt1a
Ppargc1a
Ucp3
Tfam
Uqcrc2
Ndufs2
Atp5a1
Lipogenic genes
Fatty acid oxidation genes
Mitochondrial genes

Figure S3

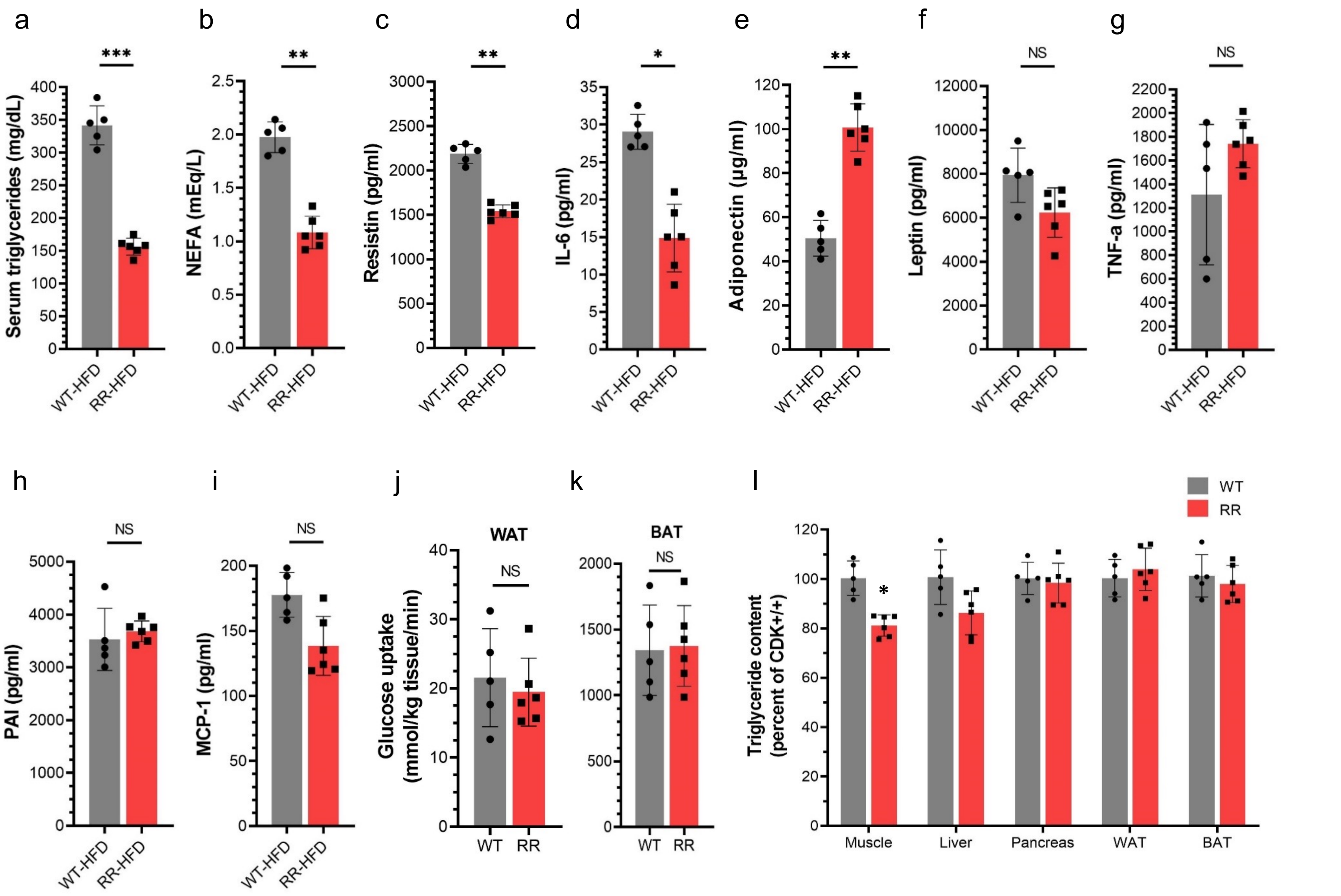

a
b
c
d
e
f
g
h
i
j
k
l
Serum triglycerides (mg/dL)
NEFA (mEq/L)
Resistin (pg/ml)
IL-6 (pg/ml)
Adiponectin (μg/ml)
Leptin (pg/ml)
TNF-a (pg/ml)
PAI (pg/ml)
MCP-1 (pg/ml)
Glucose uptake (mmol/kg tissue/min)
Triglyceride content (percent of CDK+/+)
WT-HFD
RR-HFD
***
**
*
NS
WAT
BAT
WT
RR
Muscle
Liver
Pancreas

Figure S4

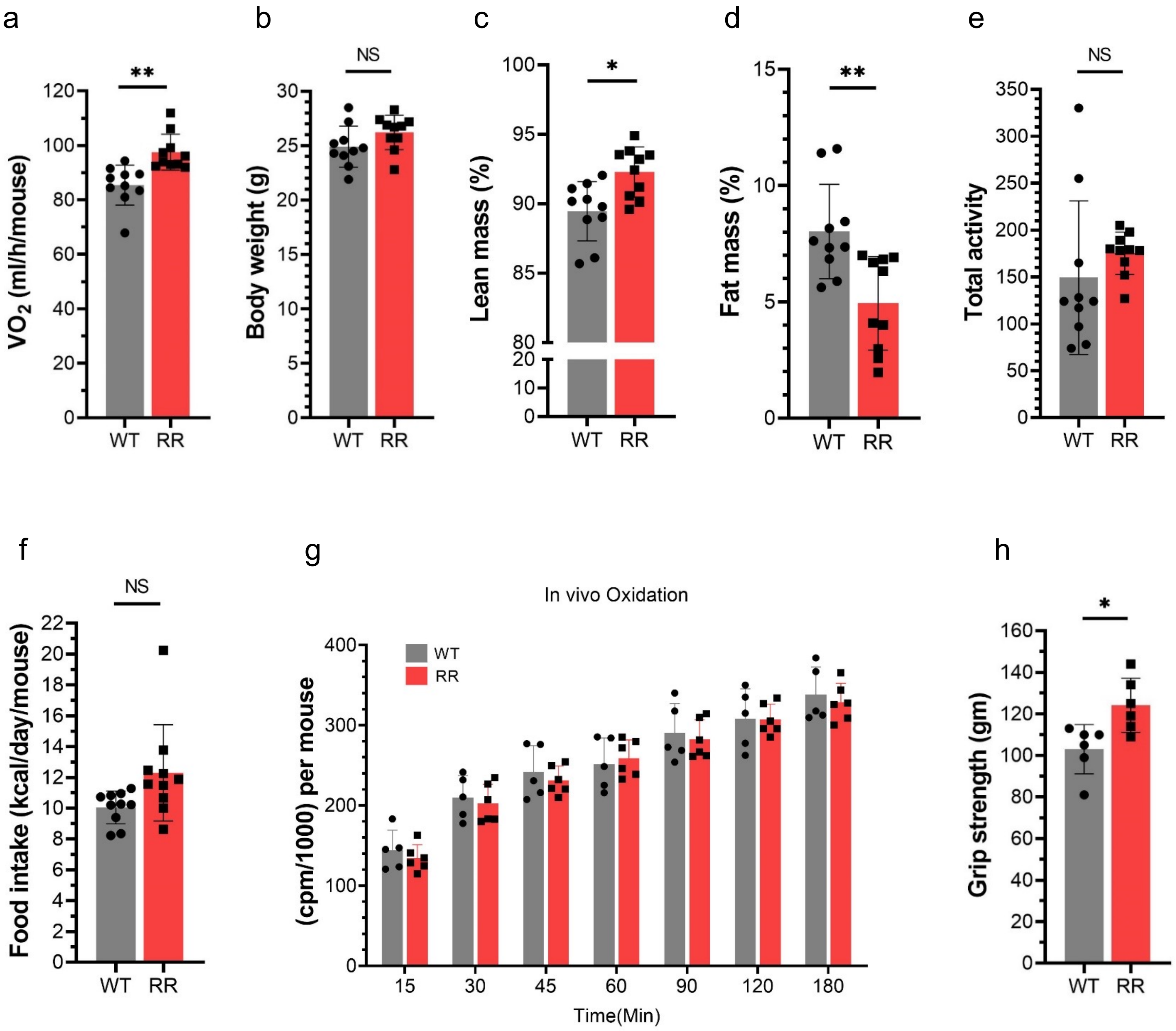
a
**
VO2 (ml/h/mouse)
120
100
80
60
40
20
0
WT
RR
b
NS
Body weight (g)
30
25
20
15
10
5
0
WT
RR
c
*
Lean mass (%)
100
95
90
85
80
20
10
0
WT
RR
d
**
Fat mass (%)
15
10
5
0
WT
RR
e
NS
Total activity
350
300
250
200
150
100
50
0
WT
RR
f
NS
Food intake (kcal/day/mouse)
22
20
18
16
14
12
10
8
6
4
2
0
WT
RR
g
In vivo Oxidation
WT
RR
(cpm/1000) per mouse
400
300
200
100
0
15
30
45
60
90
120
180
Time(Min)
h
*
Grip strength (gm)
160
140
120
100
80
60
40
20
0
WT
RR

Figure S5

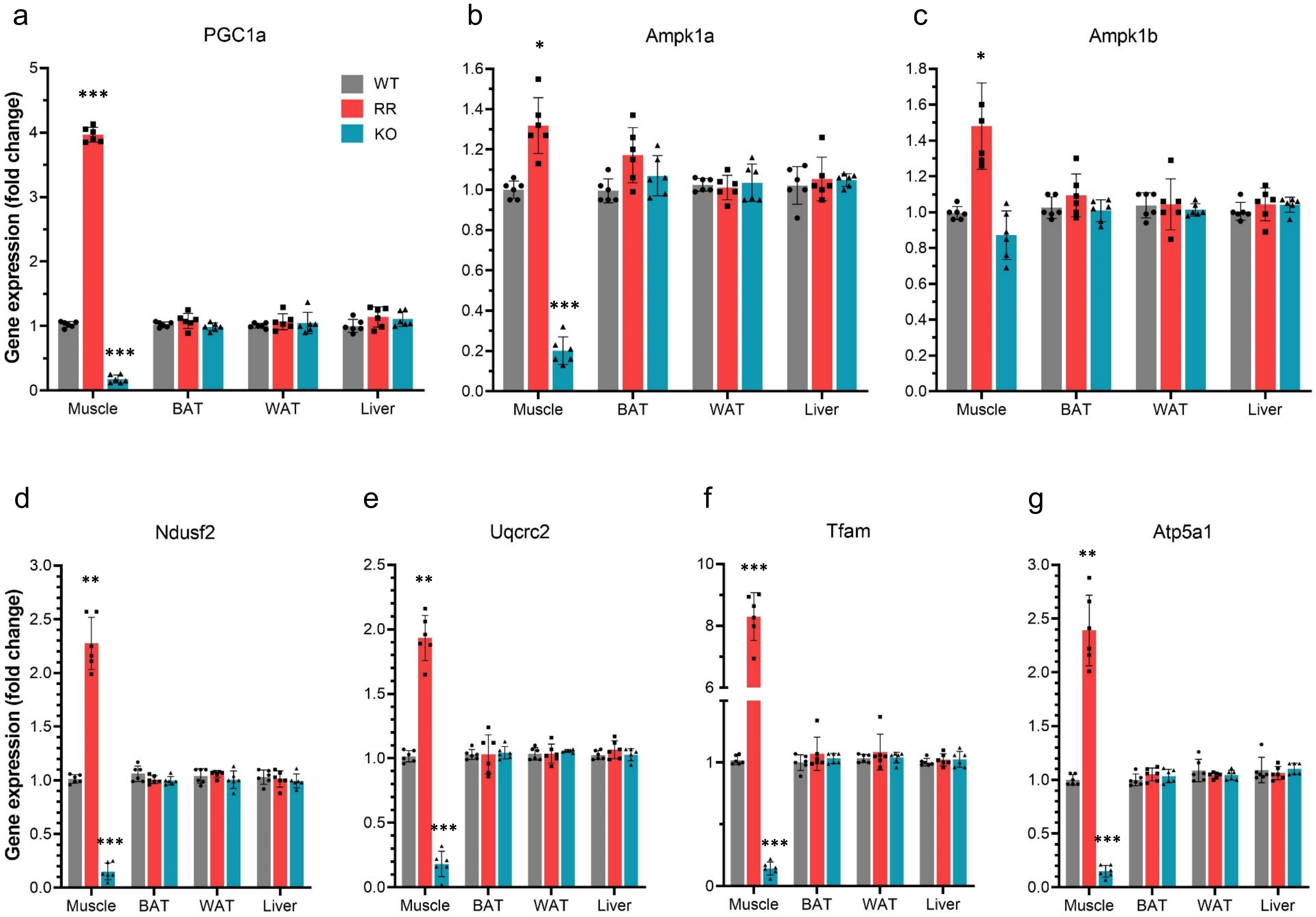
a
PGC1a
b
Ampk1a
c
Ampk1b
d
Ndusf2
e
Uqcrc2
f
Tfam
g
Atp5a1
Gene expression (fold change)
WT
RR
KO
Muscle
BAT
WAT
Liver
*
**
***

Figure S6

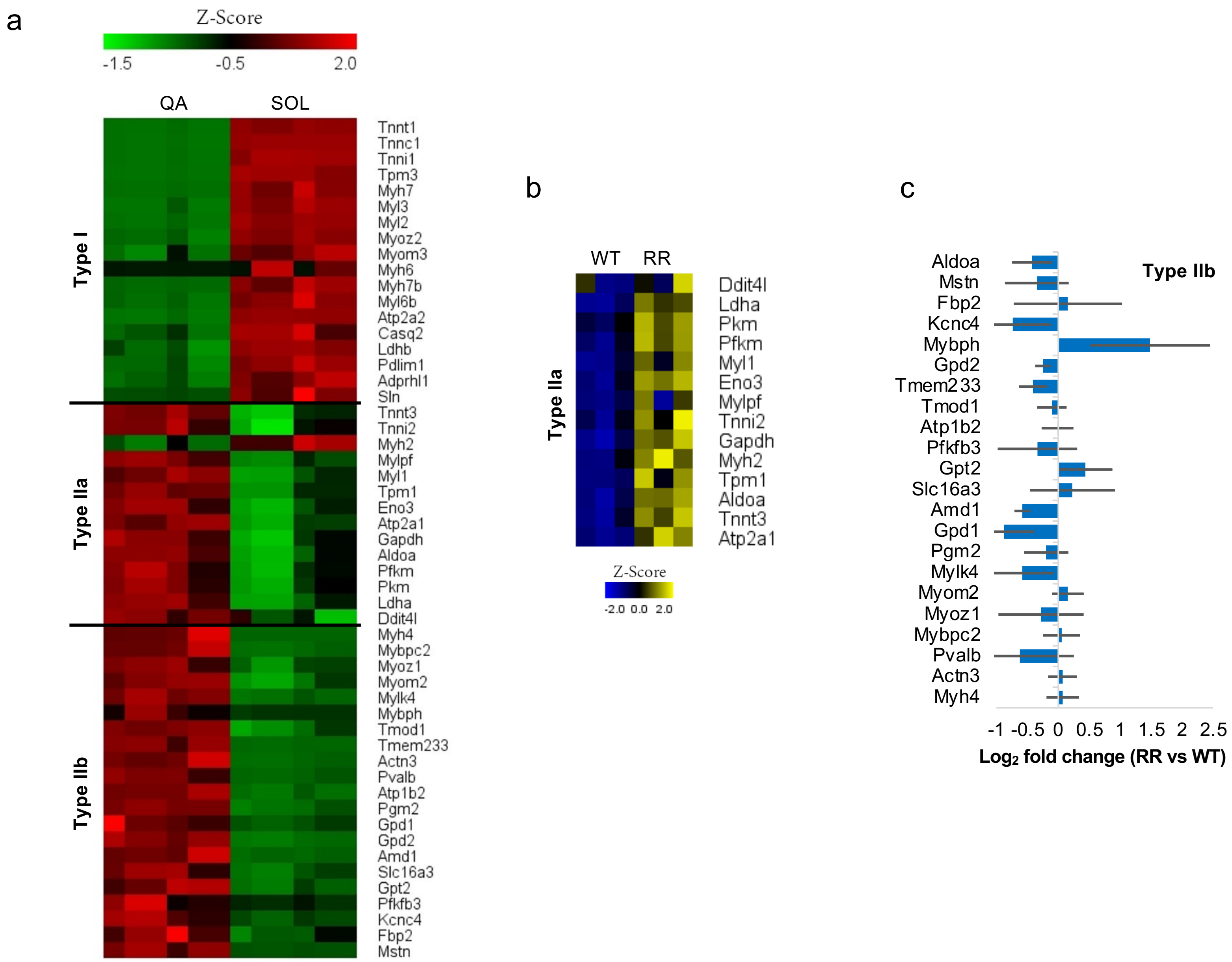

a
Z-Score
-1.5
-0.5
2.0
QA
SOL
Type I
Type IIa
Type IIb
Tnnt1
Tnnc1
Tnni1
Tpm3
Myh7
Myl3
Myl2
Myoz2
Myom3
Myh6
Myh7b
Myl6b
Atp2a2
Casq2
Ldhb
Pdlim1
Adprhl1
Sln
Tnnt3
Tnni2
Myh2
Mylpf
Myl1
Tpm1
Eno3
Atp2a1
Gapdh
Aldoa
Pfkm
Pkm
Ldha
Ddit4l
Myh4
Mybpc2
Myoz1
Myom2
Mylk4
Mybph
Tmod1
Tmem233
Actn3
Pvalb
Atp1b2
Pgm2
Gpd1
Gpd2
Amd1
Slc16a3
Gpt2
Pfkfb3
Kcnc4
Fbp2
Mstn
b
WT
RR
Type IIa
Ddit4l
Ldha
Pkm
Pfkm
Myl1
Eno3
Mylpf
Tnni2
Gapdh
Myh2
Tpm1
Aldoa
Tnnt3
Atp2a1
Z-Score
-2.0 0.0 2.0
c
Type IIb
Aldoa
Mstn
Fbp2
Kcnc4
Mybph
Gpd2
Tmem233
Tmod1
Atp1b2
Pfkfb3
Gpt2
Slc16a3
Amd1
Gpd1
Pgm2
Mylk4
Myom2
Myoz1
Mybpc2
Pvalb
Actn3
Myh4
-1 -0.5 0 0.5 1 1.5 2 2.5
Log2 fold change (RR vs WT)

Figure S7

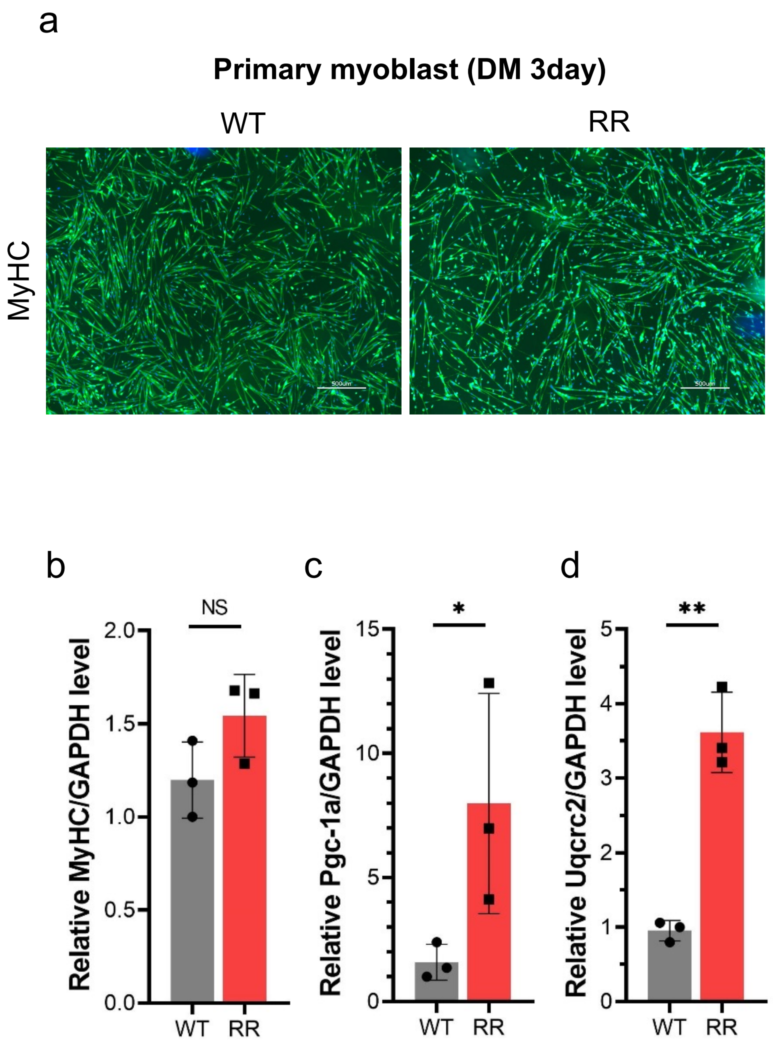
a
Primary myoblast (DM 3day)
WT
RR
MyHC
b
NS
Relative MyHC/GAPDH level
2.0
1.5
1.0
0.5
0.0
WT
RR
c
*
Relative Pgc-1a/GAPDH level
15
10
5
0
WT
RR
d
**
Relative Uqcrc2/GAPDH level
5
4
3
2
1
0
WT
RR

Figure S8

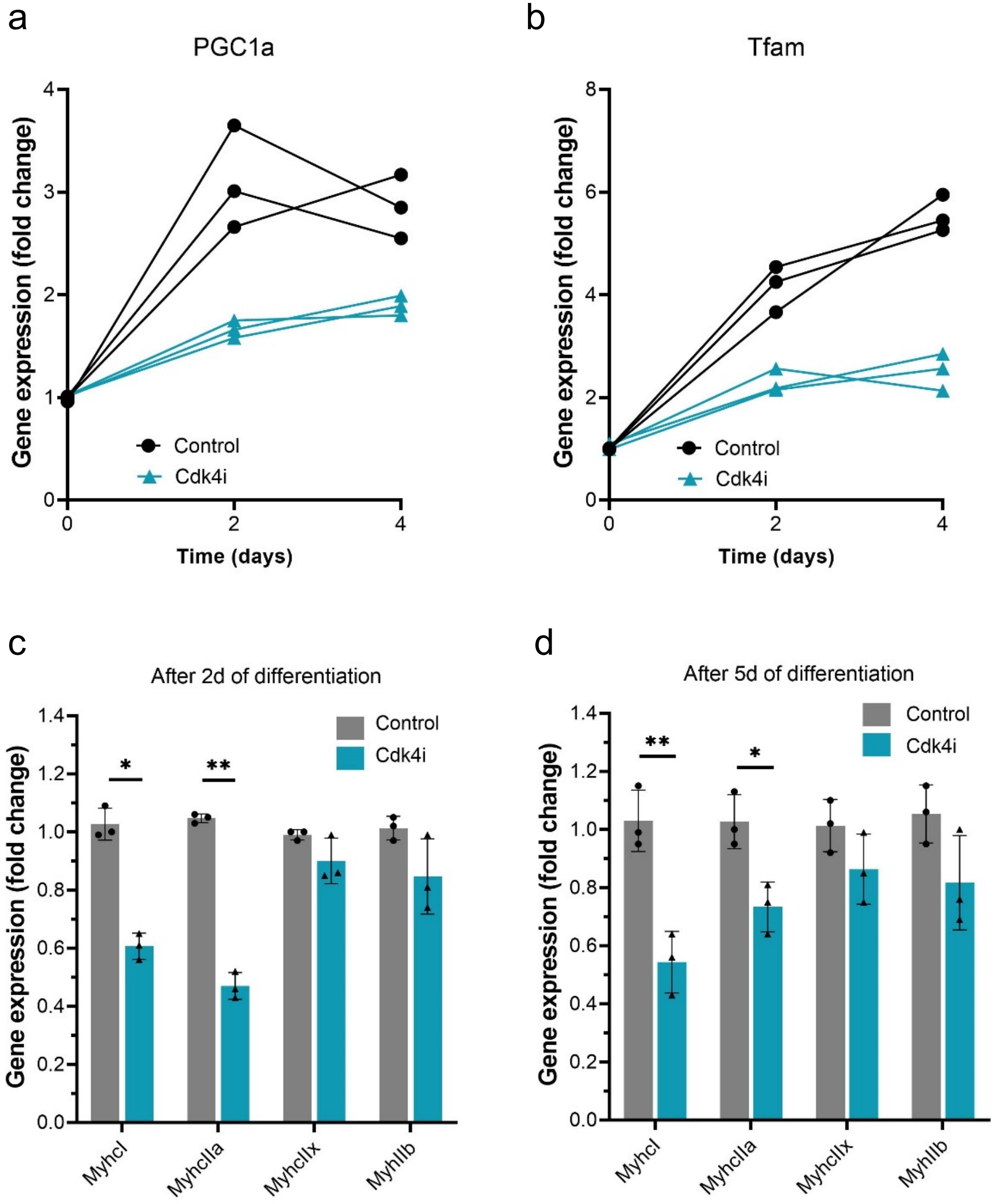
a
PGC1a
Gene expression (fold change)
Time (days)
Control
Cdk4i
b
Tfam
Gene expression (fold change)
Time (days)
Control
Cdk4i
c
After 2d of differentiation
Gene expression (fold change)
Control
Cdk4i
MyhcI
MyhcIIa
MyhcIIx
MyhIIb
d
After 5d of differentiation
Gene expression (fold change)
Control
Cdk4i
MyhcI
MyhcIIa
MyhcIIx
MyhIIb

Figure S9

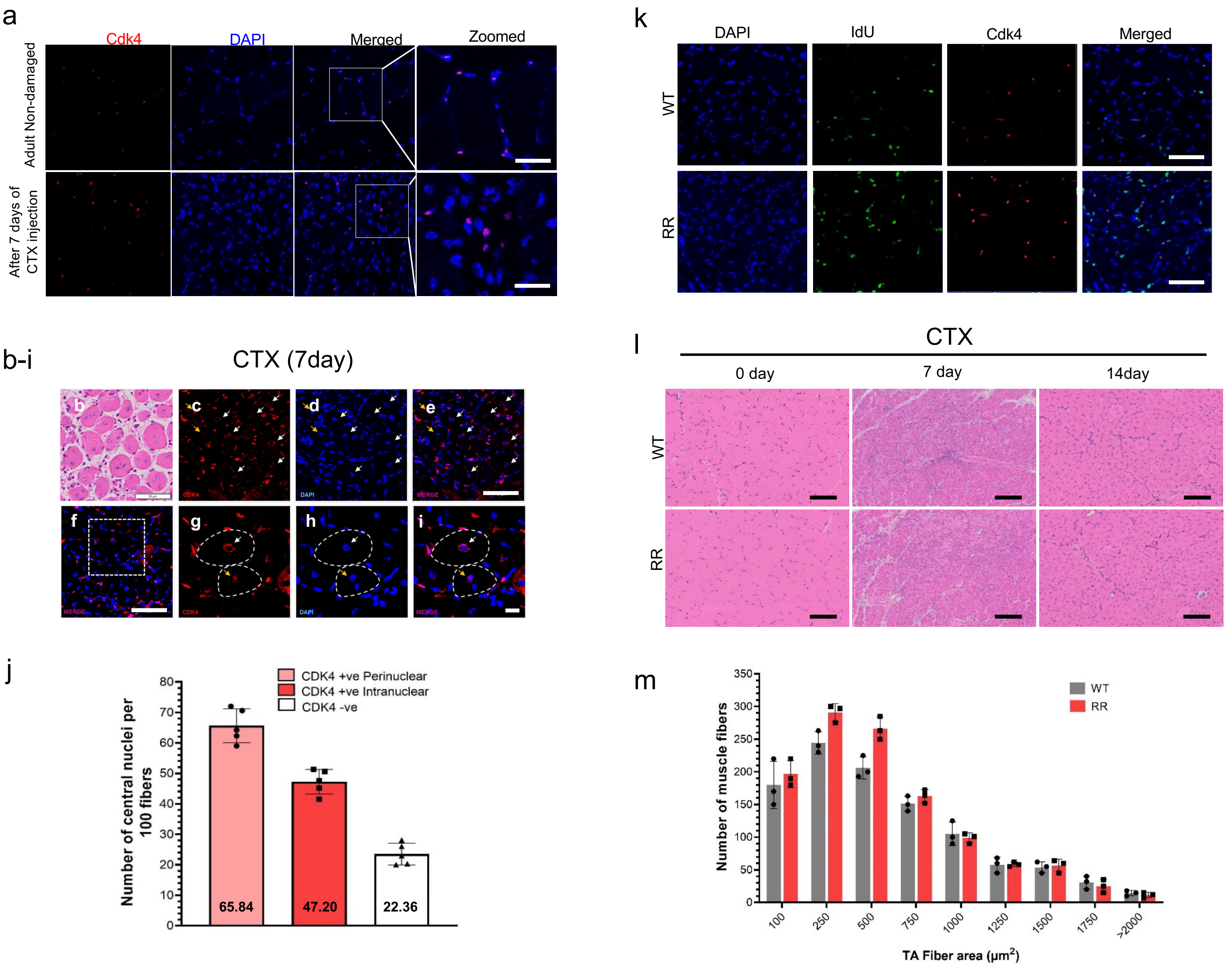

a
Cdk4
DAPI
Merged
Zoomed
Adult Non-damaged
After 7 days of CTX injection
b-i
CTX (7day)
b
c
d
e
f
g
h
i
CDK4
DAPI
MERGE
j
Number of central nuclei per 100 fibers
CDK4 +ve Perinuclear
CDK4 +ve Intranuclear
CDK4 -ve
65.84
47.20
22.36
k
DAPI
IdU
Cdk4
Merged
WT
RR
l
CTX
0 day
7 day
14day
WT
RR
m
Number of muscle fibers
WT
RR
TA Fiber area (μm2)
100
250
500
750
1000
1250
1500
1750
>2000

Figure S10

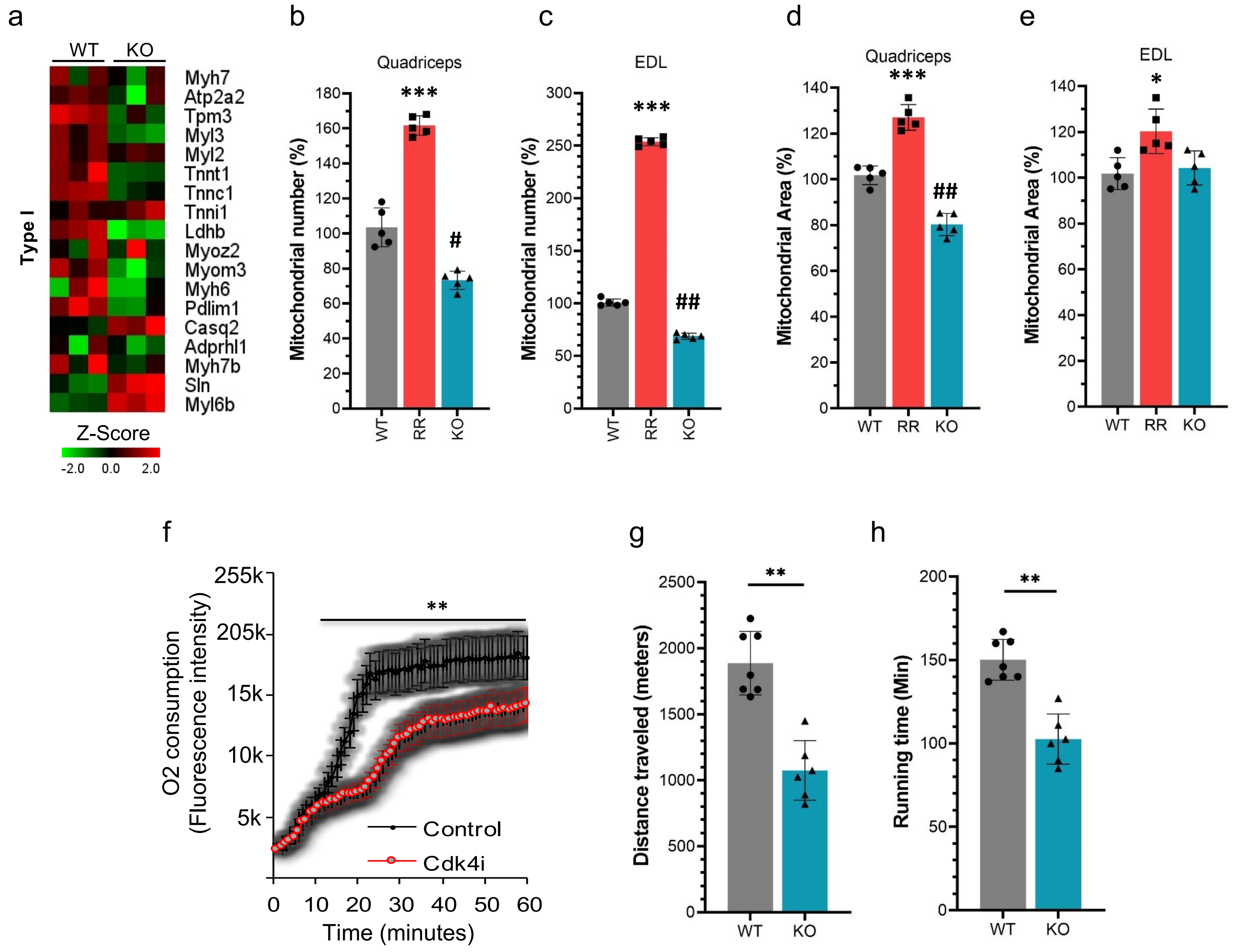
a
WT
KO
Type I
Myh7
Atp2a2
Tpm3
Myl3
Myl2
Tnnt1
Tnnc1
Tnni1
Ldhb
Myoz2
Myom3
Myh6
Pdlim1
Casq2
Adprhl1
Myh7b
Sln
Myl6b
Z-Score
-2.0 0.0 2.0
b
Quadriceps
Mitochondrial number (%)
WT RR KO
***
#
c
EDL
Mitochondrial number (%)
***
##
d
Quadriceps
Mitochondrial Area (%)
***
##
e
EDL
Mitochondrial Area (%)
*
f
O2 consumption (Fluorescence intensity)
255k
205k
15k
10k
5k
Time (minutes)
**
Control
Cdk4i
g
Distance traveled (meters)
**
WT KO
h
Running time (Min)
**
WT KO

Figure S11

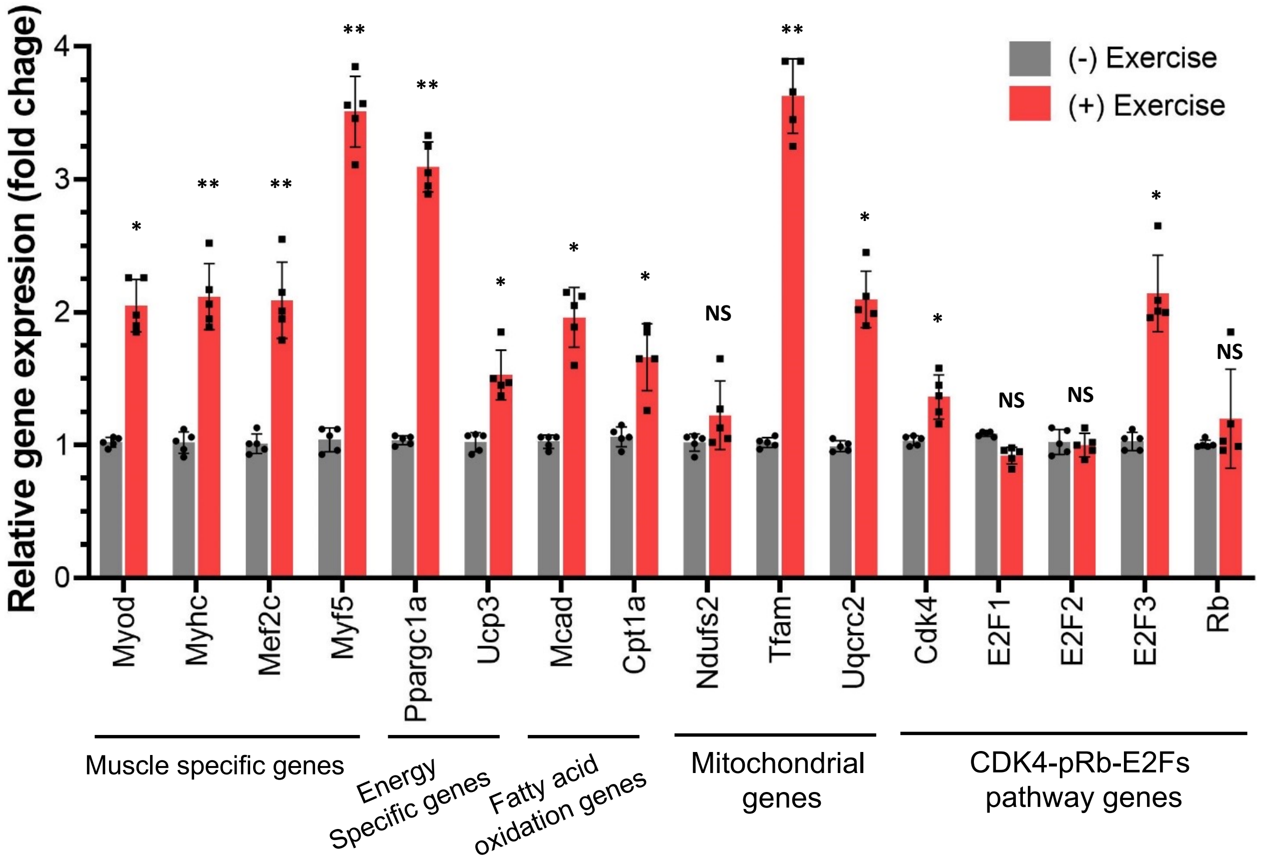

Relative gene expresion (fold chage)
0
1
2
3
4
(-) Exercise
(+) Exercise
*
**
**
**
**
*
*
*
NS
**
*
*
NS
NS
*
NS
Myod
Myhc
Mef2c
Myf5
Ppargc1a
Ucp3
Mcad
Cpt1a
Ndufs2
Tfam
Uqcrc2
Cdk4
E2F1
E2F2
E2F3
Rb
Muscle specific genes
Energy Specific genes
Fatty acid oxidation genes
Mitochondrial genes
CDK4-pRb-E2Fs pathway genes

Figure S12

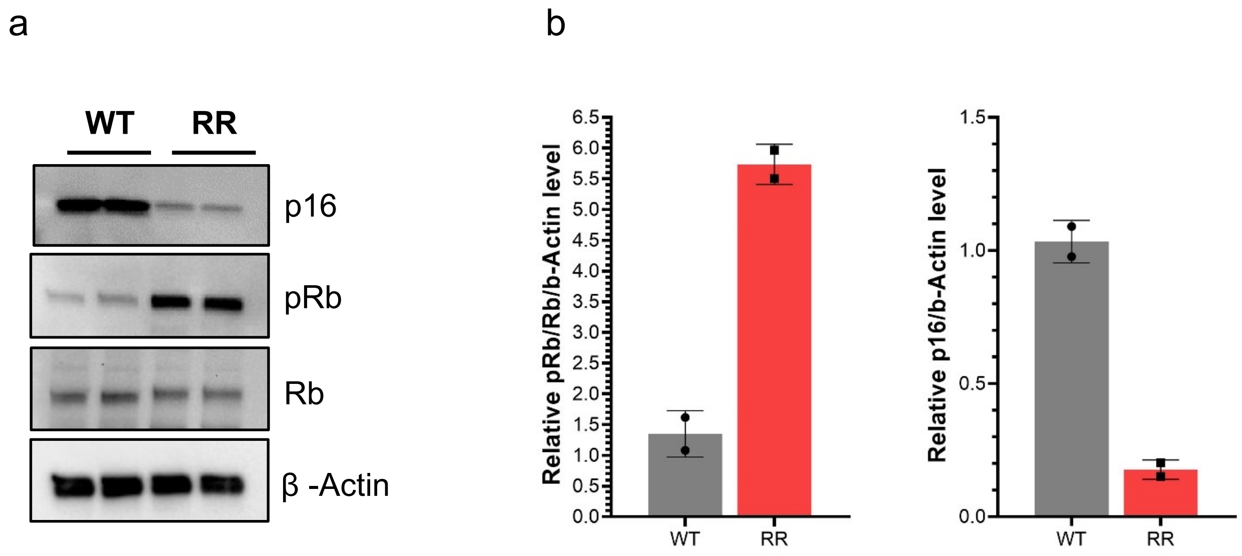
a
WT
RR
p16
pRb
Rb
β -Actin
b
Relative pRb/Rb/b-Actin level
6.5
6.0
5.5
5.0
4.5
4.0
3.5
3.0
2.5
2.0
1.5
1.0
0.5
0.0
WT
RR
Relative p16/b-Actin level
1.5
1.0
0.5
0.0
WT
RR

Figure S13

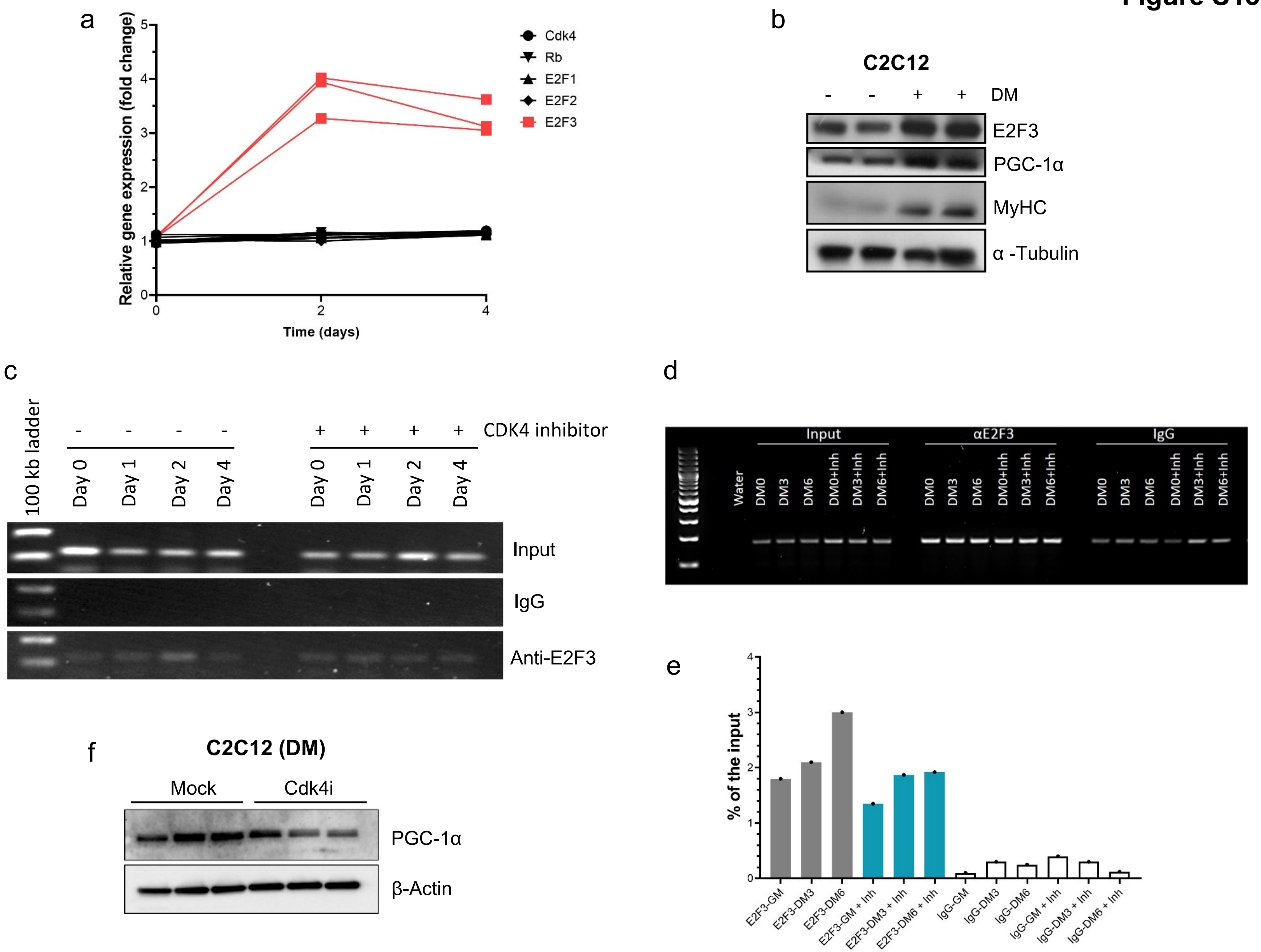
a
Relative gene expression (fold change)
Time (days)
Cdk4
Rb
E2F1
E2F2
E2F3
b
C2C12
- - + + DM
E2F3
PGC-1α
MyHC
α -Tubulin
c
100 kb ladder
Day 0
Day 1
Day 2
Day 4
CDK4 inhibitor
Input
IgG
Anti-E2F3
d
Input
αE2F3
IgG
Water
DM0
DM3
DM6
DM0+Inh
DM3+Inh
DM6+Inh
e
% of the input
E2F3-GM
E2F3-DM3
E2F3-DM6
E2F3-GM + Inh
E2F3-DM3 + Inh
E2F3-DM6 + Inh
IgG-GM
IgG-DM3
IgG-DM6
IgG-GM + Inh
IgG-DM3 + Inh
IgG-DM6 + Inh
f
C2C12 (DM)
Mock
Cdk4i
PGC-1α
β-Actin

Figure S14

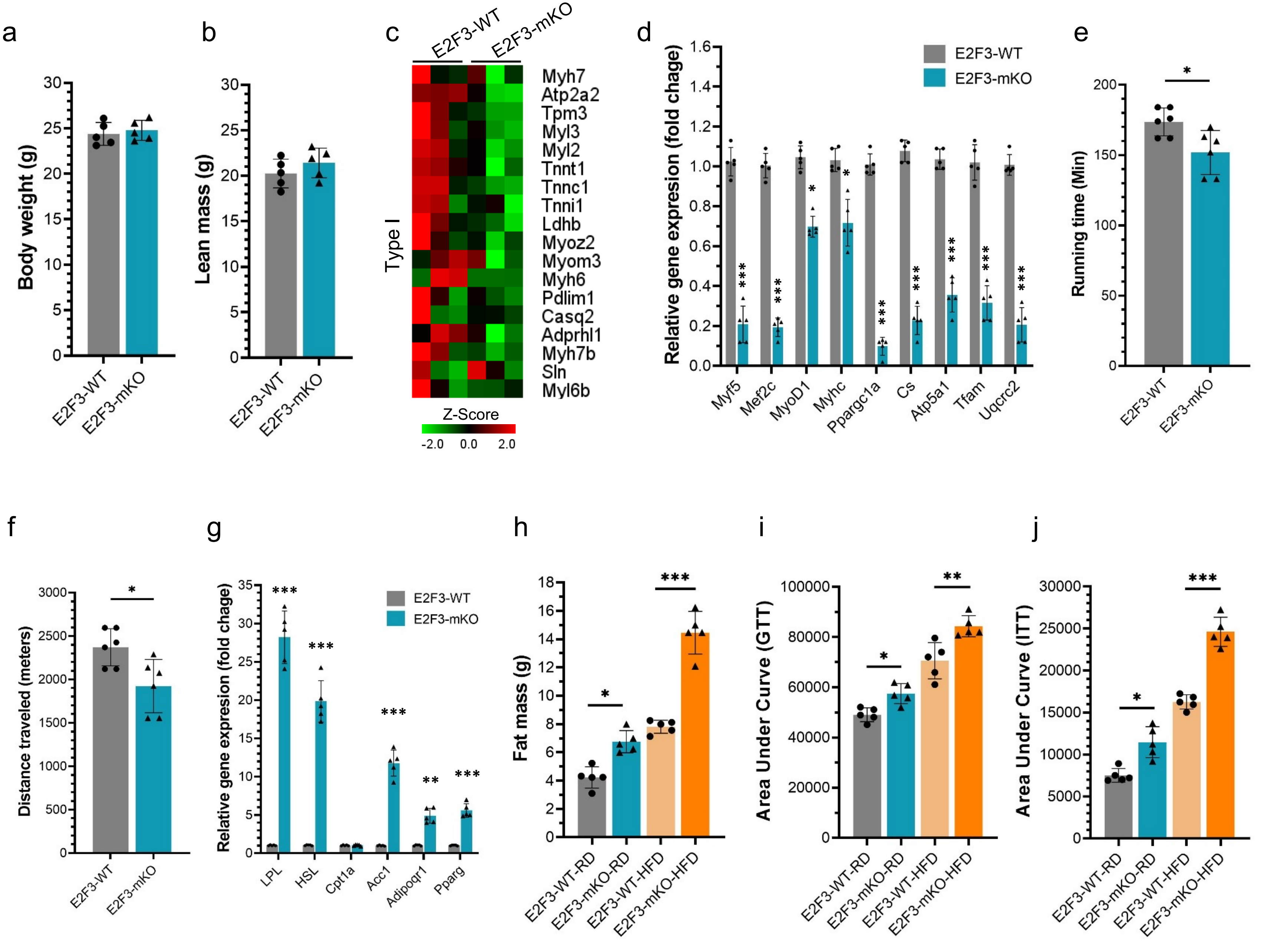
a
Body weight (g)
E2F3-WT
E2F3-mKO
b
Lean mass (g)
c
E2F3-WT
E2F3-mKO
Type I
Myh7
Atp2a2
Tpm3
Myl3
Myl2
Tnnt1
Tnnc1
Tnni1
Ldhb
Myoz2
Myom3
Myh6
Pdlim1
Casq2
Adprhl1
Myh7b
Sln
Myl6b
Z-Score
d
Relative gene expresion (fold chage)
Myf5
Mef2c
MyoD1
Myhc
Ppargc1a
Cs
Atp5a1
Tfam
Uqcrc2
e
Running time (Min)
f
Distance traveled (meters)
g
Relative gene expresion (fold chage)
LPL
HSL
Cpt1a
Acc1
Adipoqr1
Pparg
h
Fat mass (g)
E2F3-WT-RD
E2F3-mKO-RD
E2F3-WT-HFD
E2F3-mKO-HFD
i
Area Under Curve (GTT)
j
Area Under Curve (ITT)

Figure S15

a

58/483 genes

GO term	P-Value
Lipid metabolic process	5.3E-7
Fatty acid metabolic process	9.6E-7
Glucose metabolic process	1.6E-5
lipid storage	2.1E-5
Fatty acid beta-oxidation	5.0E-4
Response to fatty acid	1.4E-3
Fatty acid transport	4.4E-3

b

58 lipid-related genes

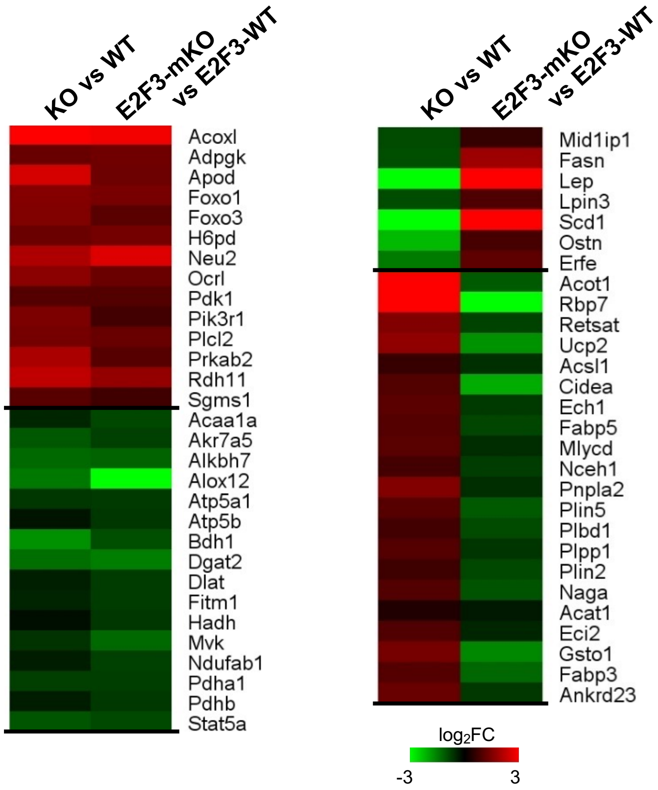

Figure S16

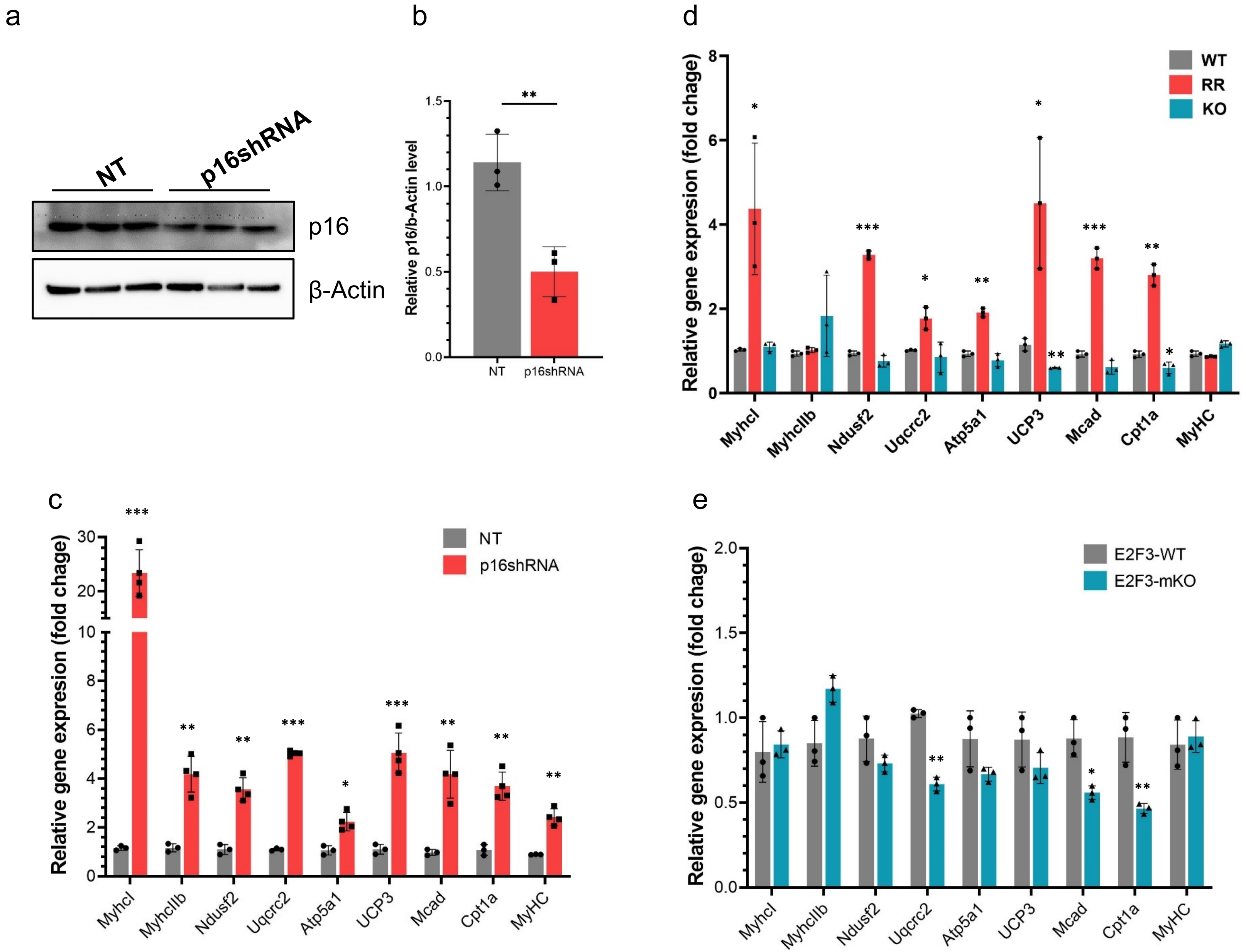
a
NT
p16shRNA
p16
β-Actin
b
**
Relative p16/b-Actin level
1.5
1.0
0.5
0.0
NT
p16shRNA
d
WT
RR
KO
Relative gene expression (fold chage)
8
6
4
2
0
*
***
*
**
*
**
***
**
*
Myhcl
Myhcllb
Ndusf2
Uqcrc2
Atp5a1
UCP3
Mcad
Cpt1a
MyHC
c
NT
p16shRNA
Relative gene expression (fold chage)
30
20
10
8
6
4
2
0
***
**
**
***
*
***
**
**
**
Myhcl
Myhcllb
Ndusf2
Uqcrc2
Atp5a1
UCP3
Mcad
Cpt1a
MyHC
e
E2F3-WT
E2F3-mKO
Relative gene expression (fold chage)
2.0
1.5
1.0
0.5
0.0
**
*
**
Myhcl
Myhcllb
Ndusf2
Uqcrc2
Atp5a1
UCP3
Mcad
Cpt1a
MyHC

Figure S17

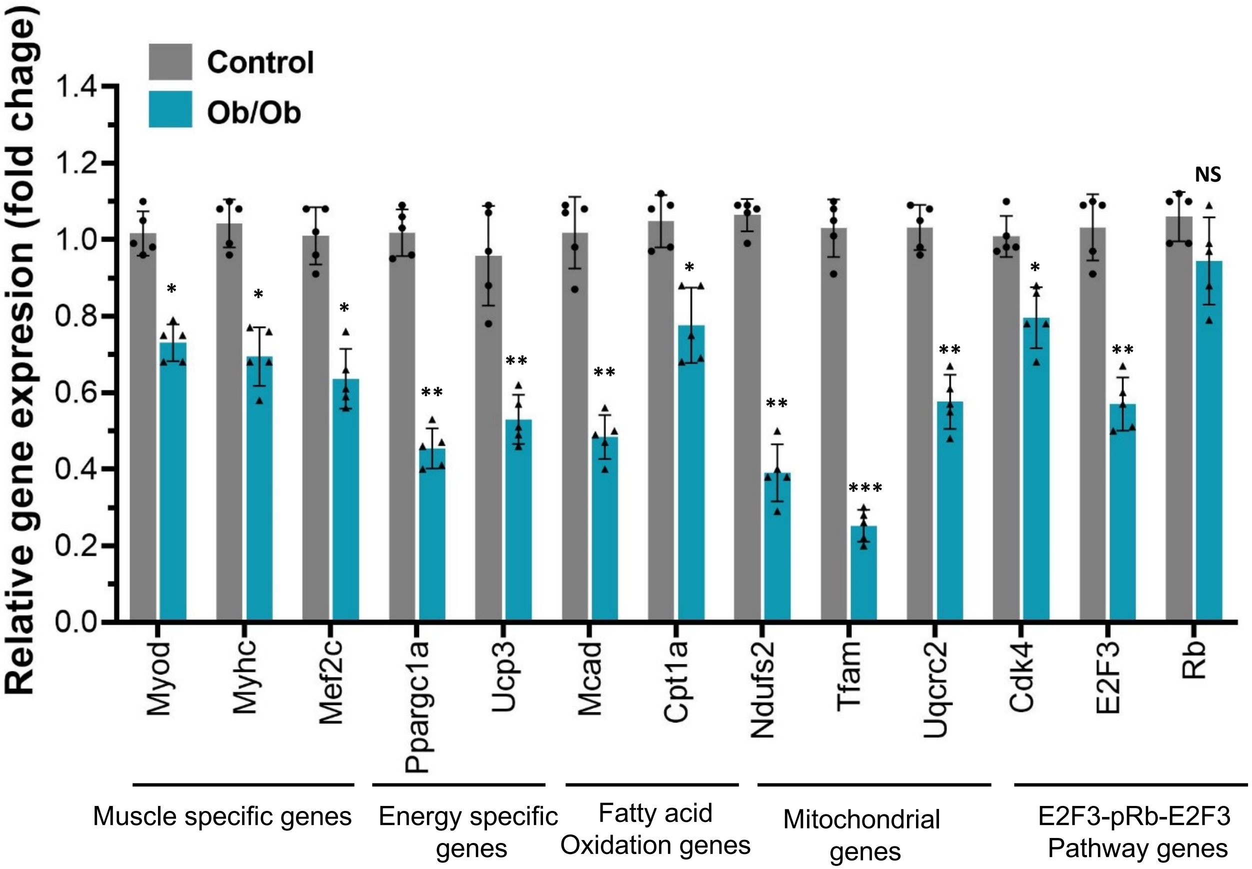

Control
Ob/Ob
Relative gene expresion (fold chage)
1.4
1.2
1.0
0.8
0.6
0.4
0.2
0.0
NS
*
**
***
Myod
Myhc
Mef2c
Ppargc1a
Ucp3
Mcad
Cpt1a
Ndufs2
Tfam
Uqcrc2
Cdk4
E2F3
Rb
Muscle specific genes
Energy specific genes
Fatty acid Oxidation genes
Mitochondrial genes
E2F3-pRb-E2F3 Pathway genes

Figure S18

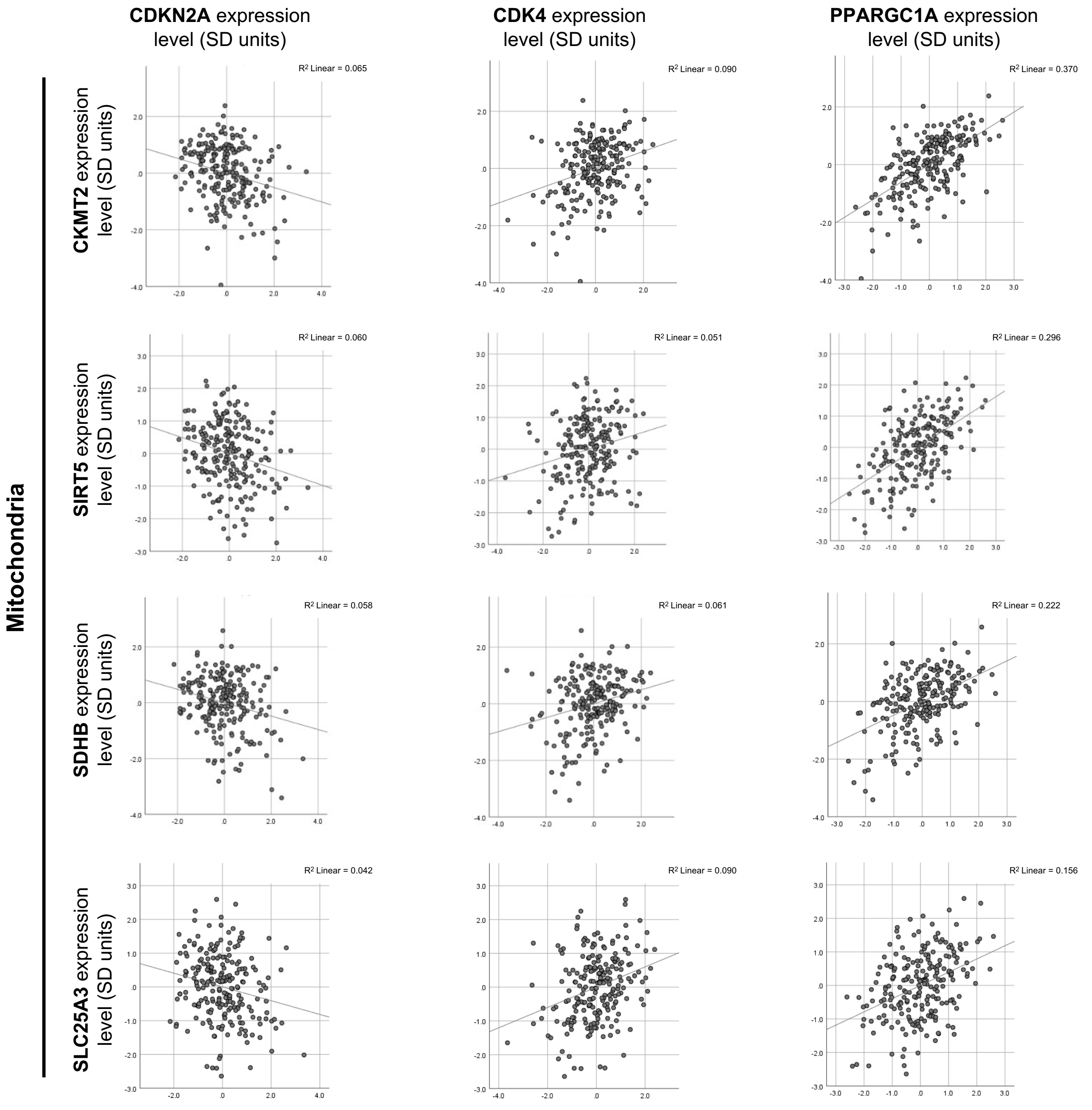

CDKN2A expression level (SD units)
CDK4 expression level (SD units)
PPARGC1A expression level (SD units)
Mitochondria
CKMT2 expression level (SD units)
SIRT5 expression level (SD units)
SDHB expression level (SD units)
SLC25A3 expression level (SD units)
R2 Linear = 0.065
R2 Linear = 0.090
R2 Linear = 0.370
R2 Linear = 0.060
R2 Linear = 0.051
R2 Linear = 0.296
R2 Linear = 0.058
R2 Linear = 0.061
R2 Linear = 0.222
R2 Linear = 0.042
R2 Linear = 0.090
R2 Linear = 0.156

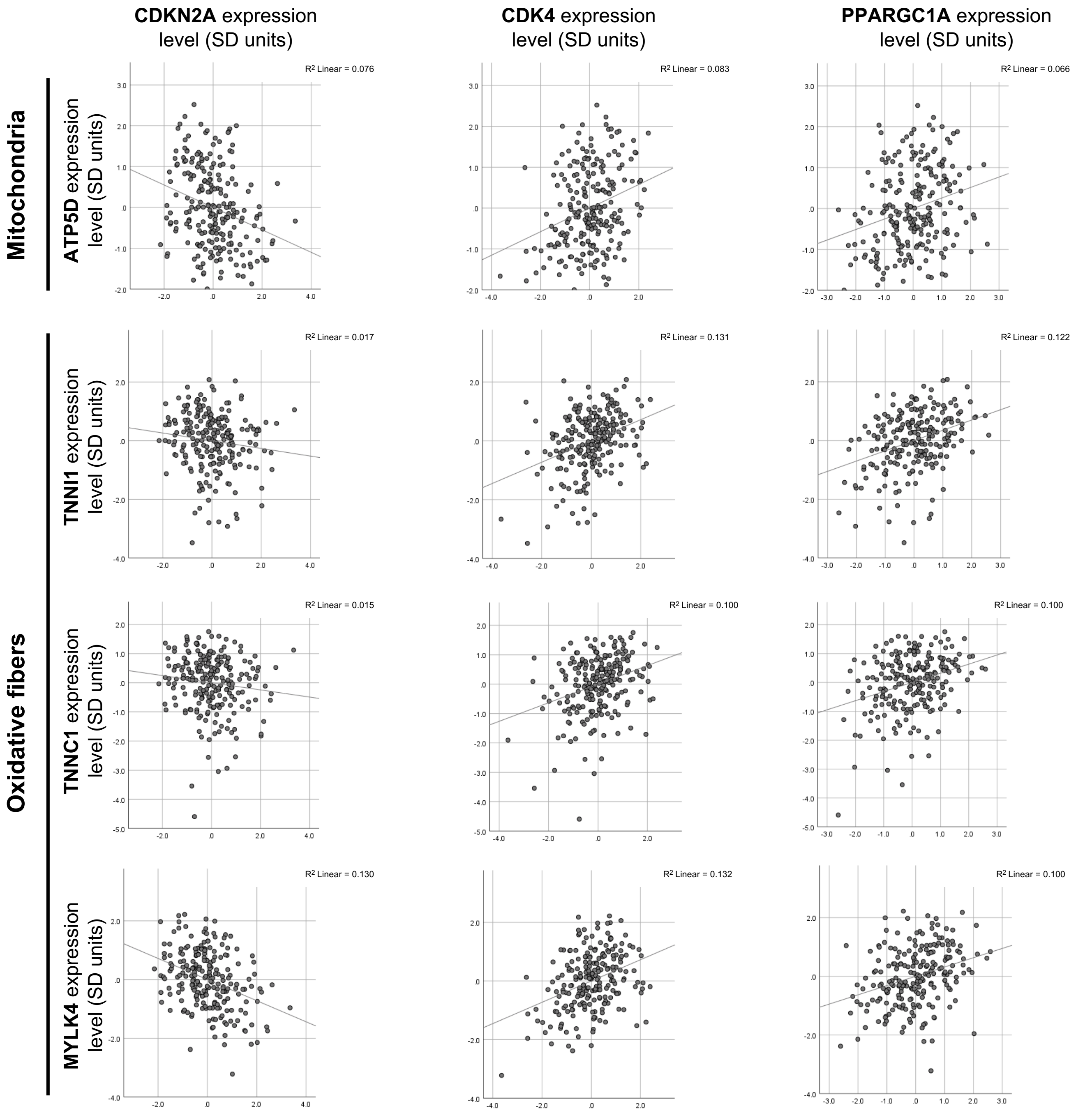
CDKN2A expression level (SD units)
CDK4 expression level (SD units)
PPARGC1A expression level (SD units)
Mitochondria
Oxidative fibers
ATP5D expression level (SD units)
TNNI1 expression level (SD units)
TNNC1 expression level (SD units)
MYLK4 expression level (SD units)
R2 Linear = 0.076
R2 Linear = 0.083
R2 Linear = 0.066
R2 Linear = 0.017
R2 Linear = 0.131
R2 Linear = 0.122
R2 Linear = 0.015
R2 Linear = 0.100
R2 Linear = 0.100
R2 Linear = 0.130
R2 Linear = 0.132
R2 Linear = 0.100

Figure S18

Figure S18

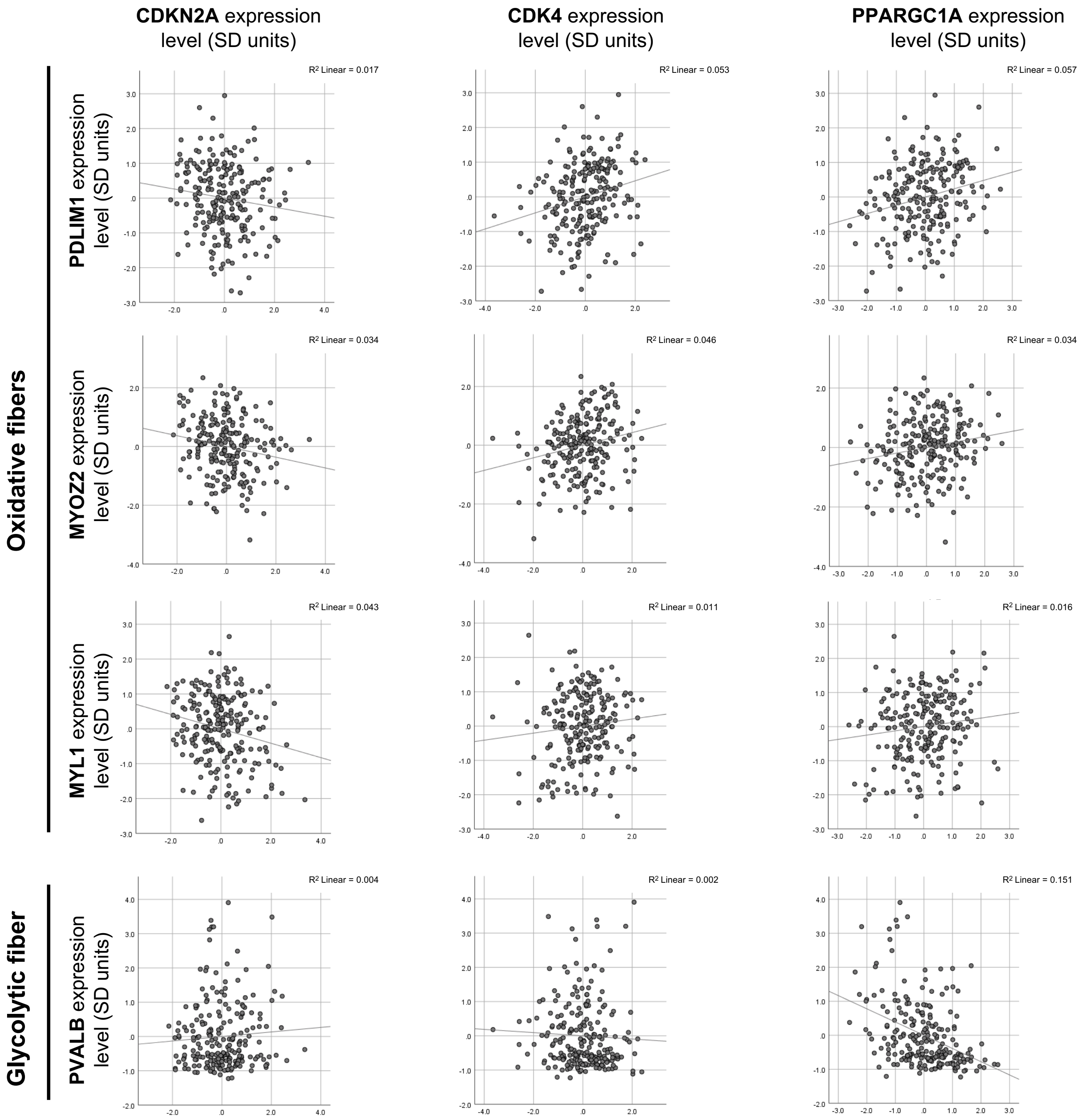

CDKN2A expression level (SD units)
CDK4 expression level (SD units)
PPARGC1A expression level (SD units)
Oxidative fibers
Glycolytic fiber
PDLIM1 expression level (SD units)
MYOZ2 expression level (SD units)
MYL1 expression level (SD units)
PVALB expression level (SD units)
R^2 Linear = 0.017
R^2 Linear = 0.053
R^2 Linear = 0.057
R^2 Linear = 0.034
R^2 Linear = 0.046
R^2 Linear = 0.034
R^2 Linear = 0.043
R^2 Linear = 0.011
R^2 Linear = 0.016
R^2 Linear = 0.004
R^2 Linear = 0.002
R^2 Linear = 0.151

Figure S18

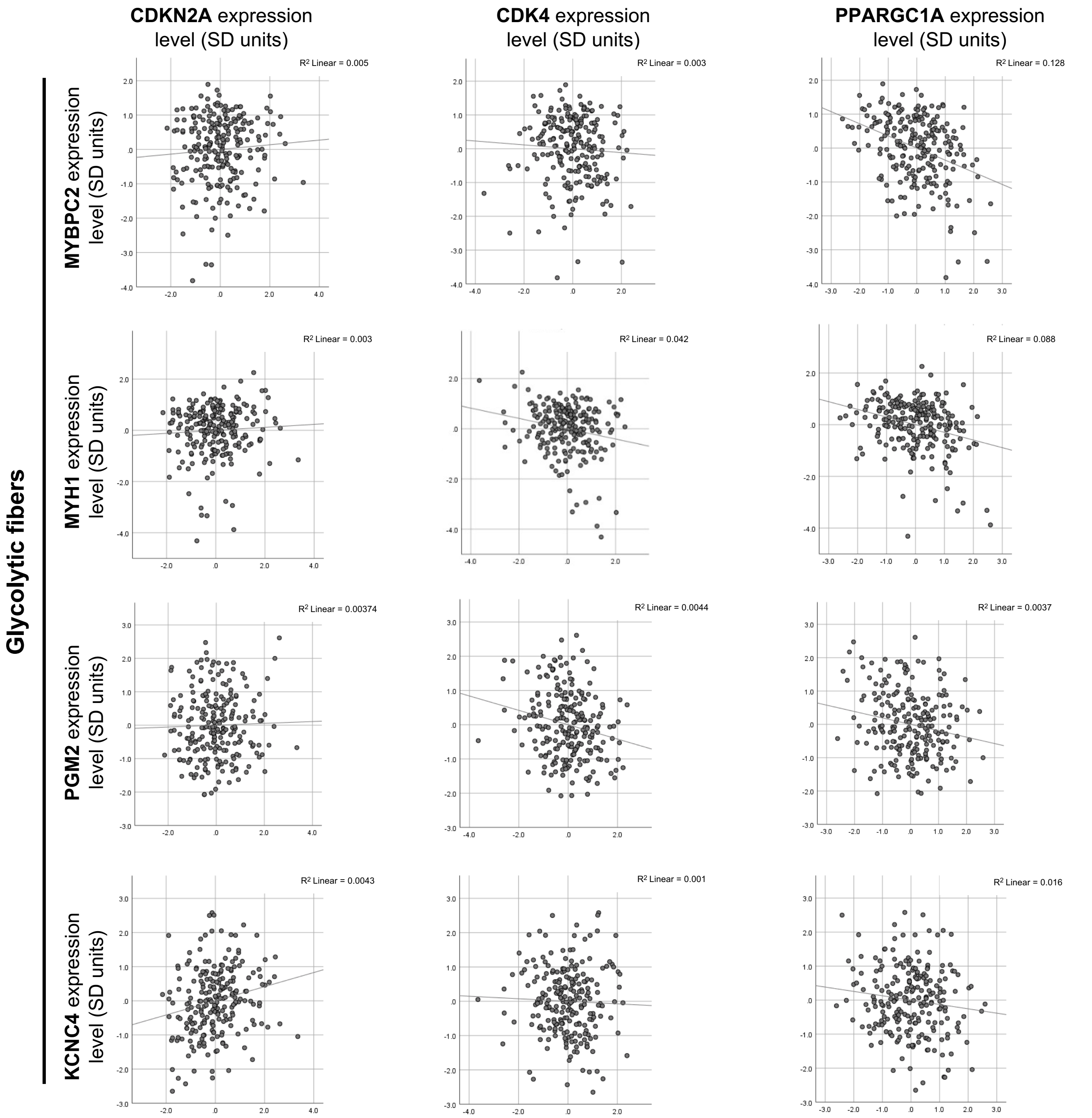
CDKN2A expression level (SD units)
CDK4 expression level (SD units)
PPARGC1A expression level (SD units)
Glycolytic fibers
MYBPC2 expression level (SD units)
MYH1 expression level (SD units)
PGM2 expression level (SD units)
KCNC4 expression level (SD units)
R2 Linear = 0.005
R2 Linear = 0.003
R2 Linear = 0.128
R2 Linear = 0.003
R2 Linear = 0.042
R2 Linear = 0.088
R2 Linear = 0.00374
R2 Linear = 0.0044
R2 Linear = 0.0037
R2 Linear = 0.0043
R2 Linear = 0.001
R2 Linear = 0.016

Supplementary Figure Legends

Supplemental Figure 1. Metabolic phenotype of *Cdk*4^{RR} mice.

(**A-H**) Body length (**A**), body weight (**B**), fasting and fed glucose levels (**C** and **D**), serum insulin level (**E**), food intake (**F**), areas under the curve (AUC) during glucose tolerance and insulin tolerance tests (**G** and **H**) in *Cdk4*^{RR} (RR) mice compared to wild-type (WT) littermates (n=5 mice per genotype). (**I-K**) Serum triglyceride (**I**), free fatty acid (**J**) and adiponectin levels (**K**) in RR mice as compared to WT mice (n=6 mice per genotype). Values plotted here are mean and standard error of means. P values are defined as * = <0.05, ** = <0.01, *** = <0.001. NS: Not significant.

Supplemental Figure 2. Improved metabolism in high-fat diet fed *Cdk*4^{RR} mice.

(**A-G**) Food intake (**A**), ambulatory activity (**B**), serum insulin levels (**C**), areas under curve during the GTT and ITT (**D and E**) in RR mice fed with HFD in comparison to that seen in similarly fed-WT mice (n=6-9 mice per genotype). (**F**) Triglyceride content in liver, muscles, pancreas, WAT and BAT in HFD-fed RR mice in comparison to that seen in HFD-fed WT mice (n=6 mice per genotype). (**G**) mRNA expression of lipogenic genes, fatty acid oxidation genes and mitochondrial genes in skeletal muscles (quadriceps) of HFD-fed RR mice as compared to that seen in similarly fed-WT mice (n=6 mice per genotype). Values plotted here are mean and standard error of means. p values, * = <0.05, ** = <0.01, *** = <0.001.

Supplemental Figure 3: Improved serum lipid and cytokine profile in *Cdk*4^{RR} mice.

(**A-I**) Levels of serum triglyceride (**A**), free fatty acids (**B**), resistin (**C**), IL-6 (**D**), adiponectin (**E**), leptin (**F**), TNF-α (**G**), PAI-1 (**H**) and MCP-1 (**I**) in HFD-fed WT and RR mice. (**J-K**) Levels of glucose uptake

in (**J**) white adipose tissue (WAT) and (**K**) brown adipose tissue (BAT) during hyperinsulinemic-euglycemic clamp assay in chow-fed RR and WT mice. (**L**) Triglyceride accumulation in skeletal muscles (quadriceps) of RR mice, compared to that seen in WT mice. (n=5-6 mice per genotype) Values plotted here are mean and standard error of means. p values, * =<0.05, ** = <0.01, *** = <0.001. NS: Not significant.

Supplemental Figure 4: Improved muscle metabolic function in *Cdk*4^{RR} mice.

(A-F) VO_2 levels (**A**), body weight (**B**), lean mass **(C)**, fat mass **(D)**, total activity **(E)**, food intake **(F)** in RR mice compared to WT mice (n=10 mice per genotype). (**G** and **H**) *In vivo* whole-body fatty acid oxidation (**G**), and grip strength **(H)** in RR mice compared to WT mice (n=5-6 mice per genotype). Spontaneous physical activity of WT and RR mice measured as beam breaks every 15 minutes in indirect calorimetry cages on four successive days (n=10 mice per genotype). All data are expressed as mean ±SEM. Values plotted here are mean and standard error of means. P values are defined as * = <0.05, ** = <0.01. NS: Not significant.

Supplemental Figure 5. Expression of metabolic genes in skeletal muscles of *Cdk*4 mutant mice.

(**A-G**) mRNA levels of (**A**) PGC-1α, (**B**) Ampk1α, (**C**) Ampk1β, (**D**) Ndufs2, (**E**) Uqcrc2, (**F**) Tfam, and (**G**) Atp5a1 in quadricep muscles, BAT, WAT and liver tissues from WT, RR and KO mice (n=6 mice per genotype). Values plotted here are mean and standard error of means. P values are defined as * = <0.05, ** = <0.01, *** = <0.001.

Supplemental Figure 6. RNA-seq analyses of quadriceps (QA) and soleus (SOL) muscles.

(**A**) Heatmap of muscle fiber-type transcripts in quadricep (QA) and soleus (SOL) muscles (n=6, wild-type mice). Color intensities indicate Z-score. (**B**) Heatmap of SOL muscle transcripts representing

fiber type IIa (n=3 mice per genotype). (**C**) Log2-fold change values determined RNAseq for Type IIb fiber markers between WT vs RR mice (n=3 mice per genotype). Values plotted here are mean and standard error of means.

Supplemental Figure 7. Myogenic differentiation capacity of primary myoblasts.

(**A**) Myogenic differentiation ability of primary myoblasts from RR and WT mice based on staining with antibody (green) against MyHC, a myogenic differentiation marker. Scale bar, 500µm. (**B-D**) Quantification of MyHC, PGC1α and Uqcrc2 protein levels during muscle differentiation of primary myoblasts from RR and WT mice. Values plotted here are mean and standard error of means. P values are defined as * = <0.05, ** = <0.01. NS: Not significant.

Supplemental Figure 8. Effects of pharmacological inhibition of Cdk4 on muscle differentiation of C2C12 cells.

(**A, B**) Effects of CDK4i treatment on expression levels of (**A**) PGC1a and (**B**) Tfam transcripts during C2C12 differentiation (n=3 each group). (**C, D**) Gene expression of slow/oxidative muscle fiber-specific myosin heavy chain isoform transcripts i.e. (MyhcI and MyhcIIa) and fast/glycolytic fiber transcripts (MyhcIIx and MyhcIIb) in Cdk4i-treated differentiating C2C12 myotubes after (**C**) 2 days and (**D**) 5 days of differentiation induction compared to expression levels in control vehicle-treated cells (n=3 treatments per group). Values plotted here are mean and standard error of means. P values are defined as * = <0.05 and ** = <0.01

Supplemental Figure 9: Cdk4 promotes muscle regeneration in response to CTX-induced injury.

(**A**) Cdk4 (red staining) expression in mature myotubes from undamaged muscle and regenerating

myotubes 7-days after Cardiotoxin (CTX) administration. DAPI stain (blue) shows nuclei. Scale bar in Zoomed image, 50µm. (**B**) Representative bright field image of H&E staining of newly formed fibers with central nuclei, 7 days post-CTX-induced muscle injury. Scale bar, 50µm. (**C-E**) Confocal (40x) images showing Cdk4 staining (red) in central nuclei (blue) of newly formed fibers. Cdk4 is localized either in the perinuclear area of central nuclei (white arrows) or inside the central nuclei (yellow arrows). Cdk4-negative central nuclei are also detected. Scale bar, 50µm. (**F-I**) Dashed square (in **F**) represents magnified images in panels (**G-I**) which are 80X confocal images showing perinuclear (white arrow) and intranuclear (yellow arrow) staining of Cdk4 in newly regenerated fibers. Dashed shapes indicate the perimeter of different fibers. Scale bar, 10µm. (**J**) Number of central nuclei with different types of Cdk4 localizations. Total number of fibers counted: 161. Data presented as mean ± SEM. (**K**) Proliferation activity in muscles after CTX-injury. Cdk4 (red) and IdU (green) antibodies with DAPI (blue) 7 days post-CTX injection. Scale bar, 50µm. (**L**) H&E staining of regenerated TA muscle from WT and RR mice 7 and 14 days post-CTX injection. Scale bar, 200µm. (**M**) Fiber area frequency distribution in cross-sectional area of TA muscle 14 days after CTX injection (n=3 per genotype). n=5-6 mice per genotype in all data shown in this figure.

Supplemental Figure 10: Improved mitochondrial phenotype in *Cdk*4^{RR} skeletal muscle.

(**A**) Heatmap of muscle type I fiber transcripts in *Cdk4*^{WT} and *Cdk4*^{KO} mice. Color intensities indicate Z-score (n=3 mice per genotype). (**B-E**) Mitochondrial numbers (**B, C**) and mitochondrial area (**D, E**) in quadriceps (**B, D**) and EDL (**C, E**) skeletal muscles from RR and KO mice, compared to WT mice (n = 5 mice per genotype). Values plotted are mean and standard error of means. (**F**) Oxygen consumption in Cdk4-inhibitor IDCX treated C2C12 cells differentiated towards muscle compared to C2C12 cells treated with control vehicle (n=3 treatments per condition). (**G, H**) Run distance **(G)** and

run time **(H)** on treadmill in KO mice, compared to WT mice (n = 6-7 mice per genotype). P values are defined as * = <0.05, ** = <0.01, *** = <0.001 WT vs RR, and # = <0.05, ## = <0.01 WT vs KO.

Supplemental Figure 11. Gene expression of selected markers in response to exercise.

Gene expression analyses of muscle, energy-specific, fatty acid oxidation, mitochondrial and cell cycle genes in quadriceps muscles of normal wild-type mice subjected to treadmill exercise (n=5 mice per group). Values plotted here are mean and standard error of means. p values, * = <0.05, ** = <0.01. NS: Not significant.

Supplementary Figure 12. Levels of p16^{Ink4a} and RB protein in primary myoblasts.

(**A)** Protein expression of p16^{Ink4a}, Ser780-phosphorylated RB (pRb), total RB and (**B**) quantification of pRb/Rb and p16 protein levels in primary myoblasts from TA muscles of RR mice as compared to WT mice. β-Actin is shown as loading control.

Supplemental Figure 13. Regulation of PGC-1α transcription by E2F3.

(**A)** mRNA expression of Cdk4, Rb, and E2F1, E2F2, and E2F3 and (**B**) protein expression of E2F3, PGC-1α, MyHC, and α-Tubulin in differentiating C2C12 myoblasts (n = 3 per condition). (**C**) Representative agarose gel images of ChIP-assay for E2F3 binding on the PGC-1α promoter during the 4-day muscle differentiation program in C2C12 cells. (**D** and **E**) Real-time qPCR detected binding of E2F3 to the PGC-1α promoter during muscle differentiation of C2C12 cells with or without the presence of a Cdk4 inhibitor (Inh). GM = Growth medium, DM3 and DM6 = 3 and 6 days in differentiation medium (DM), IgG = control immunoglobulin antibody precipitated sample. E2F3 =

E2F3 antibody precipitated sample. (**F**) Representative western blot of PGC-1α expression in differentiating C2C12 myoblasts after treatment with Cdk4 inhibitor (Cdk4i) in comparison to mock vehicle treatment (n=3 samples per condition). P values are defined as * = <0.05.

Supplemental Figure 14. Effects of skeletal muscle specific deletion of E2F3 on metabolism. Body weight (**A**) and lean mass (**B**) of E2F3WT and E2F3mKO mice (n=5 mice per genotype). (**C**) Heatmap of muscle fiber type I transcripts in E2F3WT and E2F3mKO mice. Color intensities indicate Z-score (n=3 mice per genotype). (**D**) mRNA expression levels of muscle specific genes (Myf5, Mef2c, MyoD1, Myh1), mitochondrial specific genes (citrate synthase (Cs), ATP synthase (Aat5b), Tfam and Uqcrc2), and PGC-1α in QA muscle of WT and E2F3mKO mice (n = 5 per genotype). Running time (**E**) and running distance (**F**) after treadmill exercise by E2F3mKO and E2F3WT mice (n=6 per genotype). (**G**) Expression of lipogenesis-specific gene transcripts (Lpl, Hsl, Acc1, Adipoqr1(Adiponectin receptors) and Ppar-γ) in QA skeletal muscles of E2F3mKO mice, compared to wild type counterparts (n=5 per genotype). (**H**) Total body fat mass in E2F3mKO mice on regular diet (RD) and on high fat diet (HFD). Area under the curve during the GTT (**I**) and ITT (**J**) in E2F3mKO mice, compared to E2F3WT mice (n=5 per genotype). Values plotted here are mean and standard error of means. P values are defined as * = <0.05, **= <0.01, *** = <0.001.

Supplemental Figure 15. Correlation of lipid-profile gene expression with Cdk4 and E2F3 status. (**A**) GO analysis values of 58 genes from 483 differentially expressed genes (DEGs, p <0.05) common in Cdk4KO and E2F3mKO muscle compared to their respective wild-type controls. (**B**) Heatmap of the 58 genes showing relative expression levels based on Cdk4 and E2F3 status. Left heatmap panel shows genes that are either co-upregulated or co-downregulated based on Cdk4 and E2F3 status. Right

heatmap shows genes that are oppositely upregulated or downregulated based on Cdk4 and E2F3 status. The black line in both panels distinguishes the co-upregulation vs co-downregulation (left heatmap panel) and the opposite upregulation vs downregulation. The color intensities indicate Log2 fold change (n=3 mice per genotype).

Supplemental Figure 16. Muscle and mitochondrial genes expression in deafferented primary myoblasts.

(**A and B**) Efficacy of p16ink4a knockdown in primary myoblasts shown via (**A**) western blot analyses and (**B**) its quantification (n=3 each group). β-Actin is shown as loading control. (**C-E**) Relative expression levels of transcripts representing muscle and mitochondrial genes in differentiated primary myoblasts from (**C**) p16 knock-downed cells, (**D**) $Cdk4^{WT}$, $Cdk4^{RR}$, and $Cdk4^{KO}$ cells, and (**E**) $E2F3^{WT}$, $E2F3^{mKO}$ cells (n=3-4 each group). Values plotted here are mean and standard error of means. p values, * = <0.05, ** = <0.01, *** =<0.001.

Supplemental Figure 17. Gene expression levels of select markers in muscle from $Lep^{Ob/Ob}$ mice.

Expression of transcripts representing muscle and energy specific, fatty acid oxidation, mitochondrial and cell cycle genes in QA muscles from $Lep^{Ob/Ob}$ (Ob/Ob) mice compared with age and sex-matched wild-type control mice (n= 5 mice per genotype). Values plotted here are mean and standard error of means. p values, * = <0.05, ** = <0.01, *** =<0.001. NS: Not significant.

Supplemental Figure 18. Correlation of mRNA expression levels in human muscle biopsies.

Scatter plots correlating mRNA expression levels of markers representing 5 mitochondria genes, 6 oxidative fibers genes and 5 glycolytic fibers genes in skeletal muscle biopsies from human subjects

(n=221) with levels of expression of *CDKN2A*, *CDK4* and *PPARGC1A*. Smaller circles in scatter plots denote genes expression level of all human subjects. The best fit line in scatter plots was calculated via linear regression analysis and is shown along with goodness of fit (R^2 statistic).

Supplementary Tables

Table S1: human subject characteristics (n=49)

**Age, years**	41.1	±	12.1
**Sex, % female**		51%	
**Body Composition**			
**BMI, kg/m^2**	32.4	±	8.8
**% body fat**			
**Females**	42.3	±	9.4
**Males**	32.2	±	10.5
**Insulin sensitivity**			
**Fasting glucose, mg/dL**	91.1	±	11.1
**Fasting insulin, mcU/mL**	9.3	±	7.5
**Hemoglobin A1C, %**	5.5	±	0.5
**HOMA-IR**	2.2	±	1.9
**Lipids**			
**Total cholesterol, mg/dL**	174	±	34
**LDL, mg/dL**	106	±	29
**HDL, mg/dL**	47	±	12
**Triglycerides, mg/dL**	122	±	74
**Free fatty acids, mcEq/L**	579	±	189
**Creatine kinase, ng/mL**	128	±	90

Data presented as mean ± standard deviation

Table S2: Association between muscle E2F3 and PGC-1α mRNA expression and metabolic parameters (n=49)

Parameter	E2F3		PGC-1α	
	r	p	r	p
**Body composition**				
**BMI**	-0.444	0.001	-0.601	<0.001
**% body fat**	-0.282	0.049	-0.556	<0.001
**Total body fat mass**	-0.408	0.004	-0.656	<0.001
**L. vastus lipid content**	-0.729	0.001	-0.707	0.003
**L. vastus IMCL***	-0.675	0.004	-0.755	0.001
**Insulin sensitivity****	0.302	0.044	0.512	<0.001
**VO_2-max**	0.256	0.106	0.465	0.003

Associations assessed by linear regression. *IMCL = intramyocellular lipid content **Insulin sensitivity determined by frequently-sampled intravenous glucose tolerance test (FSIVGTT)

Table S3: Antibodies used in this study

No.	Antibody name	Company
1	pIRS1	Cell Signaling
2	IRS1	Cell Signaling
3	pAkt	Cell Signaling
4	Akt	Cell Signaling
5	α-Tubulin	Sigma
6	MyhcI	Developmental Studies Hybridoma Bank(DSHB), The University of Iowa
7	MyhcIIa	Developmental Studies Hybridoma Bank(DSHB), The University of Iowa
8	MyhcIIb	Developmental Studies Hybridoma Bank(DSHB), The University of Iowa
9	Myosin (Fast)	Sigma
10	Myosin (Slow)	Sigma
11	Laminin	Sigma
12	Myogenin	Cell Signaling
13	Myf5	Cell Signaling
14	MyoD	Cell Signaling
15	MyHC	Santacruz Biotechnology
16	CDK4	Santacruz Biotechnology, Cell signaling
17	Cytochrome Oxidase	Molecular probe
18	PGC1α	Santacruz Biotechnology
19	E2F1	Santacruz Biotechnology
20	E2F2	Santacruz Biotechnology
21	E2F3	Santacruz Biotechnology
22	pRb	Millipore, Cell signaling
23	Total Rb	Cell Signaling
24	Cyclin D1	Cell Signaling
25	Cyclin D2	Cell Signaling
26	Cyclin D3	Cell Signaling
27	p16	Santacruz Biotechnology
28	UQCRC2	Santacruz Biotechnology
29	mtTFA	Santacruz Biotechnology
30	GAPDH	Santacruz Biotechnology
31	β-Actin	Santacruz Biotechnology

Table S4: Primers used in this study

Gene name	Gene Symbol	Primer Sequence	
		Forward (5'→3')	Reverse (5'→3')
Myosin Heavy Chain, Type I	Myh7	CCAGGGGCAAACA GGCATTCACT	CTTCCACTGGGCC ACTTCACTGTT
Myosin Heavy Chain, Type IIa	Myh2	ATGAGCTCCGACG CCGAG	TCTGTTAGCATGA ACTGGTAGGCG
Myosin Heavy Chain, Type IIb	Myh4	GTGATTTCTCCTGT CACCTCTC	GGAGGACCGCAAG AACGTGCTGA
Myosin Heavy Chain, Type IIx	Myh1	TGAAGGGCGGCAA GAAGCAGAT	GCGGAATTTGGCC AGGTTGACA
Mitochondrial NADH-ubiquinone oxidoreductase chain 1	ND1	CTCTTATCCACGCT TCCGTTACG	GATGGTGGTACTC CCGCTGTA
Mitochondrial Cytochrome b	Cytb	GTGAACGATTGCT AGGGCC	CGATTCTTCGCTTT CCACTTCAT
Mitochondrial H19	H19	GTACCCACCTGTC GTCC	GTCCACGAGACCA ATGACTG
NADH dehydrogenase (ubiquinone) Fe-S protein 2	Ndufs2	CAGCCAGATATTG AATGGGCA	TGTTGGTCACCGC TTTTTCCT
Ubiquinol cytochrome c reductase core protein 2	Uqcrc2	AAAGTTGCCCCGA AGGTTAAA	GAGCATAGTTTTC CAGAGAAGCA
ATP synthase, H+ transporting, mitochondrial F1 complex, alpha subunit 1	Atp5a1	TCTCCATGCCTCTA ACACTCG	CCAGGTCAACAGA CGTGTCAG
Transcription factor A, mitochondrial	Tfam	ATTCCGAAGTGTT TTTCCAGCA	TCTGAAAGTTTTG CATCTGGGT
Myogenic differentiation 1	MyoD1	CCACTCCGGGACA TAGACTTG	AAAAGCGCAGGTC TGGTGAG
Myocyte enhancer factor 2C	Mef2c	ATCCCGATGC AGACGATTCA G	AACAGCACACAAT CTTTGCCT
Myogenic factor 5	Myf5	AAGGCTCCTGTAT CCCCTCAC	TGACCTTCTTCAG GCGTCTAC
Peroxisome proliferator-activated receptor gamma, coactivator 1 alpha (Mouse)	Ppargc1a (Pgc1α)	TATGGAGTGACAT AGAGTGTGCT	CCACTTCAATCCA CCCAGAAAG
Peroxisome proliferator-activated receptor gamma, coactivator 1 alpha (Human)	PPARGC1a (PGC1α)	TGAAGACGGATTG CCCTCATT	GCTGGTGCCAGTA AGAGCTT
Uncoupling protein 3	Ucp3	CTGCACCGCCAGA TGAGTTT	ATCATGGCTTGAA ATCGGACC
Acyl-Coenzyme A dehydrogenase, medium chain	Mcad	AGGGTTTAGTTTT GAGTTGACGG	CCCCGCTTTTGTCA TATTCCG

Palmitoyltransferase 1a	Cpt1a	CTCCGCCTGAGCC ATGAAG	CACCAGTGATGAT GCCATTCT
E2F transcription factor 1	E2f1	CTCGACTCCTCGC AGATCG	GATCCAGCCTCCG TTTCACC
E2F transcription factor 2	E2f2	ACGGCGCAACCTA CAAAGAG	GTCTGCGTGTAAA GCGAAGT
E2F transcription factor 3 (Mouse)	E2f3	AAACGCGGTATGA TACGTCCC	CCATCAGGAGACT GGCTCAG
E2F transcription factor 3 (Human)	E2F3	AAAGCCCCTCCAG AAACAAGA	CCTTGGGTACTTG CCAAATGT
Fatty acid binding protein 3	Fabp3	ACCTGGAAGCTAG TGGACAG	TGATGGTAGTAGG CTTGGTCAT
Fatty acid synthase	Fasn	GGAGGTGGTGATA GCCGGTAT	TGGGTAATCCATA GAGCCCAG
Protein kinase, AMP-activated, alpha 1 catalytic subunit	Ampk1α	GTCAAAGCCGACC CAATGATA	CGTACACGCAAAT AATAGGGGTT
Protein kinase, AMP-activated, beta 1 non-catalytic subunit	Ampk1β	AGGCCCAAGATCC TCATGGA	GGGGGCTTTATCA TTCGCTTC
Cyclin-dependent kinase 4	Cdk4	ATGGCTGCCACTC GATATGAA	TCCTCCATTAGGA ACTCTCACAC
Retinoblastoma protein	Rb	TGCATCTTTATCGC AGCAGTT	GTTCACACGTCCG TTCTAATTTG
Troponin I1 (Human)	TNNI1	GCGATACGACATT GAGGCCAA	TTCCCACGGAGGT CCATCA
Myosin Light Chain 3 (Human)	MYL3	TCACACCTGAGCA GATTGAAGA	GCTGGAGCATAGG CAGGAAAG
Troponin C1 (Human)	TNNC1	TGGTTCGGTGCAT GAAGGAC	GTCGATGTAGCCA TCAGCATT
Myozenin 2 (Human)	MYOZ2	CCAGGCTATTTAA GATGCGTCA	CCTTCCAAGTTAC TTCCATCCAC
PDZ and LIM domain 1 (Human)	PDLIM1	GCTGGCCTCTACT CTTCTGAA	GCTGAGCATGGTC TAAGGGT
Myosin light chain 1 (Human)	MYL1	GTTGAGGGTCTGC GTGTCTTT	ACCCAGGGTGGCT AGAACA
Myosin light chain kinase family member 4 (Human)	MYLK4	GCCCGTCAAAAGC AAAAGGAC	CTTGGCTGTCACA ATACGATGA
Succinate dehydrogenase complex iron sulfur subunit B (Human)	SDHB	ACCTTCCGAAGAT CATGCAGA	GTGCAAGCTAGAG TGTTGCCT
Creatine kinase, mitochondrial 2 (Human)	CKMT2	CCAAGCGCAGACT ACCCAG	GGTGTCACCTTGT TGCGAAG
Sirtuin 5 (Human)	SIRT5	TGGAGGAGGTTGA CAGAGAGC	CTGCTGGGTACAC CACAGA

NADH:ubiquinone oxidoreductase core subunit S8 (Human)	NDUFS8	CCATCAACTACCC GTTCGAGA	CCGCAGTAGATGC ACTTGG
Solute carrier family 25 member 3 (Human)	SLC25A3	TATCTCTGGCGCA CATCACTA	CTTAGCAGCTTCC ATAGGAGC
ATP synthase F1 subunit delta (Human)	ATP5D	ACTCTTCGGTGCA GTTGTTGG	GCCTCGATTCGGA TCTGGAT
Myosin heavy chain 4 (Human)	MYH4	GCTGCTTATCTGA CAAGTCTGAA	GGCCTTTGGTTAC GAACTCATT
Myosin heavy chain 1 (Human)	MYH1	GGGAGACCTAAAA TTGGCTCAA	TTGCAGACCGCTC ATTTCAAA
Potassium voltage-gated channel subfamily C member 4 (Human)	KCNC4	ATATCGACCGCAA CGTGACAG	CAGGTTCTTGACG AAGTCCAG
Phosphoglucomutase 2 (Human)	PGM2	GAGGCAGTGAAAC GACTAATAGC	CTGTCCCAAACTC CATTCGGG
Myosin binding protein C2 (Human)	MYBPC2	AAGAGCGAGTACG AGAAAATCG	TTGACCTCGACCT TAGCCTTT
Parvalbumin (Human)	PVALB	AAGAGTGCGGATG ATGTGAAG	GCCTTTTAGGATG AATCCCAGC

Group	Gene
Oxidative (slow) Fiber	ADPRHL1, ATP2A2, CASQ2, MYH6, MYH7, MYH7B, MYL2, MYL3, MYL6B, MYOM3, MYOZ2, PDLIM1, SLN, TNNC1, TNNI1, TNNT1, TPM3
Oxidative (fast) Fiber	ATP2A1, DDIT4L, ENO3, MYH2, MYL1, MYLPF, PFKM, PKM, TNNT3, TPM1
Glycolytic Fiber	ACTN3, ALDOA, AMD1, ATP1B2, FBP2, GPD1, GPD2, GPT2, KCNC4, LDHA, MSTN, MYBPC2, MYBPH, MYH1, MYH4, MYLK4, MYOM2, MYOZ1, PFKFB3, PGM2, PVALB, SLC16A3, TMOD1, TSTD2
Mitochondria	ACAT2, AKR1B10, ALKBH7, AS3MT, ATP5A1, ATP5B, ATP5D, ATP5F1, ATP5G1, ATP5G3, ATP5O, BDH1, CARD9, CHCHD10, CIAPIN1, CKMT2, CLU, COA5, COQ10B, COX4I1, COX5A, COX7A1, CSNK1D, CYC1, DGAT2, DLAT, DMPK, DNAJA3, ETHE1, FASTK, HADH, HAGH, HINT1, HSPA9, IDH3B, IMMP2L, LDHB, LDHD, LYRM7, ME3, MGARP, MGST1, MMAA, MRPL37, MRPL55, MRPS11, MRPS36, NAA15, NDUFA10, NDUFA11. NDUFA12, NDUFA9, NDUFAB1, NDUFB10, NDUFB7, NDUFC1, NDUFS2, NDUFS3, NDUFS7, NDUFS8, NDUFV1, NDUFV3, PDHB, PDHX, PEBP1, PHYH, PIN1, PRDX2, PRDX5, RHOT2, SAMM50, SDHA, SDHB, SH3BP5, SIRT5, SLC25A3, SLC25A4, SOD2, TMEM70, TXN2, UNC50, UQCRC1
Cell Cycle	CDK1, CDK2, CDK4, CDK5, E2F1, E2F3, E2F4, E2F5, E2F6, E2F7, E2F8, LPAR6, RB1, RBL1

Table S5: Markers of oxidative and glycolytic muscle fibers, muscle mitochondria and cell cycle.
